# Comprehensive Characterization of Triterpene Saponins in Rhizoma Panacis Japonici by Offline Two-Dimensional Liquid Chromatography Coupled to Quadrupole Time-of-Flight Mass Spectrometry

**DOI:** 10.3390/molecules29061295

**Published:** 2024-03-14

**Authors:** Subinuer Yasen, Chengrui Li, Siyuan Wang, Yixin Dong, Hang Li, Jie Chen, Yifan Meng, Ping Yu, Haiyan Zou

**Affiliations:** School of Traditional Chinese Medicine, Capital Medical University, Beijing 100069, China

**Keywords:** *Panax japonicus*, triterpene saponin, offline two-dimensional liquid chromatography, quadrupole time-of-flight mass spectrometry, in-house database, structural characterization

## Abstract

Rhizoma Panacis Japonici (RPJ) is an ancient herbal medicine from China that has long been employed for its medicinal benefits in relieving arthritis physical debility and diverse afflictions. The primary bioactive constituents found in RPJ are triterpene saponins, which exhibit numerous pharmacological actions, including anti-inflammatory, antioxidant, and immunomodulating effects. The present study established a straightforward and effective approach for characterizing triterpene saponins in RPJ. An offline HILIC × RP LC/QTOF-MS method was developed, along with a self-constructed in-house database containing 612 saponins reported in the Panax genus and 228 predicted metabolites. The approach achieved good chromatographic performance in isolating triterpene saponins of RPJ, with the HILIC column as the first dimension (^1^D) and the BEH C18 column as the second dimension (^2^D). The developed two-dimensional liquid chromatography system exhibited an orthogonality of 0.61 and a peak capacity of 1249. Detection was performed using a QTOF mass spectrometer in a data-independent manner (MS^E^) in a negative ion mode. Using the in-house database, the collected MS data were processed by an automatic workflow on UNIFI 1.8.2 software, which included data correction, matching of precursor and product ions, and peak annotation. In this study, 307 saponins were characterized from RPJ and 76 saponins were identified for the first time in *Panax japonicus*. This research not only enhances our understanding of the chemical characteristics of RPJ but also offers a simple and efficient method for analyzing the complex composition of herbal medicine.

## 1. Introduction

Herbal medicines (HM) have been employed in traditional medical systems across the globe for several centuries and have garnered growing acknowledgment for their therapeutic properties. These medicines typically consist of complex chemical components [1]. Elucidating the compounds present in an herb is a fundamental question to ensure its efficacy and safety in traditional Chinese medicine research [2]. With advancements in analytical technology, particularly chromatography combined with high-resolution mass spectrometry (HRMS), significant progress has been made in characterizing the chemical composition of herbal medicine. However, it is imperative to recognize that the scientific community continues to face difficulties in effectively segregating and characterizing the intricate chemical components present in HM [3].

Liquid chromatography-HRMS (LC-HRMS) is the most widely used technique for characterizing metabolites in HM, especially small metabolites. Nevertheless, the significant variations in polarity, structures, and content of chemical constituents in HM present a growing challenge for one-dimensional (^1^D) LC-MS. The peak capacity attainable with a single column or separation mechanism was insufficient for separating complex samples, making it hard to obtain MS^n^ information on minor or trace components. Moreover, the interference from coeluting components increases the difficulty in structural identification or even leads to reproducible results [4]. Therefore, enhancing separation capabilities and reducing the coelution fraction are essential for improving the sensitivity and reproducibility of LC-MS. Recently, two-dimensional (2D) LC-HRMS has emerged as a powerful tool for profiling complex chemical systems, such as natural products, biosamples, environmental contaminants and food additives [5,6,7]. 2D LC significantly increases peak capacities to several thousand or even over 10,000 by connecting two columns with different separation mechanisms in series, such as the combination of normal-phase (NP) and reversed-phase (RP), ion exchange (IE) and RP, as well as HILIC and RP [5]. Shi Qiu et al. [8] developed an offline 2D LC-MS system to analyze ginsenosides in *P. ginseng* leaves. They used HILIC-HPLC as the first dimension (^1^D) to isolate the extract into multiple fractions. After concentrated, each fraction was analyzed with the second dimension (^2^D) using RP-UHPLC/LTQ-Orbitrap MS instrument. The system showed a practical peak capacity of 11,000, leading to the characterization of 646 ginsenosides, a five-fold increase compared to those identified using only RP-UHPLC/LTQ-Orbitrap-MS. 2D LC can also be operated in online mode using special instruments that facilitate the continuous transfer of fractions from the ^1^D column to the ^2^D column. Online mode offers increased automation and throughput compared to offline mode. However, in addition to equipment requirements, the chromatographic conditions in the online mode are usually difficult to optimize due to the compatibility of mobile phases between the ^1^D and ^2^D isolation [9]. On the other hand, offline 2D LC provides flexibility, ease of operation, and the potential to perform multidimensional LC separations [10].

HRMS provides extensive coverage of chemicals, a broad dynamic range, precise mass measurements, and distinguishable isotope distributions [11]. As a result, it has been effectively utilized for untargeted analysis in various research fields, including natural product [12], proteomics [13], foodomics [6], lipidomics [5], etc. Data-dependent (DDA) and data-independent (DIA) acquisition modes are the primary data acquisition modes for untargeted metabolite analysis. The DDA mode is valuable for linking MS^2^ spectra to the precursor ions and creating MS^1^-MS^2^ datasets, but compounds with low MS abundance cannot undergo fragmentation. On the other hand, DIA modes, such as SWATH and MS^E^, can continuously and impartially capture MS^2^ information for all compounds, resulting in notably greater spectral coverage compared to DDA modes [11,14]. So, some studies have combined the two modes for improving accuracy and coverage of the structural identification in profiling chemical constituents of complex sample [15,16].

Efficient management of massive MS/MS data of 2D LC-HRMS is essential for in-depth chemical characterization of complex sample. Computer-aided database searches are commonly used for untargeted analysis. Various software and algorithms are employed for automatic processing of MS data, such as UNIFI 1.8.2 software (Waters) [17], ACD/MS Structure ID Suite (ACD/Labs) [18], as well as MS-DIAL [19], XCMS [20], and Open MS 2.0 [21]. A comprehensive database specific to the samples being analyzed is important for the identification of metabolites. While online databases like MassBank and Metline are available, additional prediction strategies have been developed to enhance database coverage. In the case of saponins, structure predictions primarily focus on substitution patterns, such as acetylation, formylation, malonylation, and types of sugar substituents. By utilizing this approach, 945 saponins, including 662 potentially novel ginsenosides, were identified from the leaves of *P. notoginseng* using UNIFI software combined with an in-house database [22].

Rhizoma Panacis Japonici (RPJ) derives from *Panax japonicus* C. A. Mey., a species of the *Araliaceae* family [23]. It has been used as a folk medicine in China for over 200 years and has been recorded in the Chinese Pharmacopoeia since 1977. RPJ and its preparations are primarily utilized in the clinical treatment of rheumatoid arthritis [24]. RPJ contains triterpenoid saponins, polysaccharides, minerals, and amino acids. Of these components, triterpene saponins are considered the primary active ingredients in RPJ, display a diverse array of pharmacological properties, including anti-inflammatory, antioxidant, and anti-myocardial ischemia effects [25,26]. We were the first to report the therapeutic effects of the total saponin from RPJ on experimental autoimmune encephalomyelitis, a classical animal model of multiple sclerosis [27]. A Chinese national invention patent (No. zl201410041725.5) has been granted for this discovery [28]. Therefore, elucidating the saponin constituents in RPJ is essential for further research.

**Figure 1:**
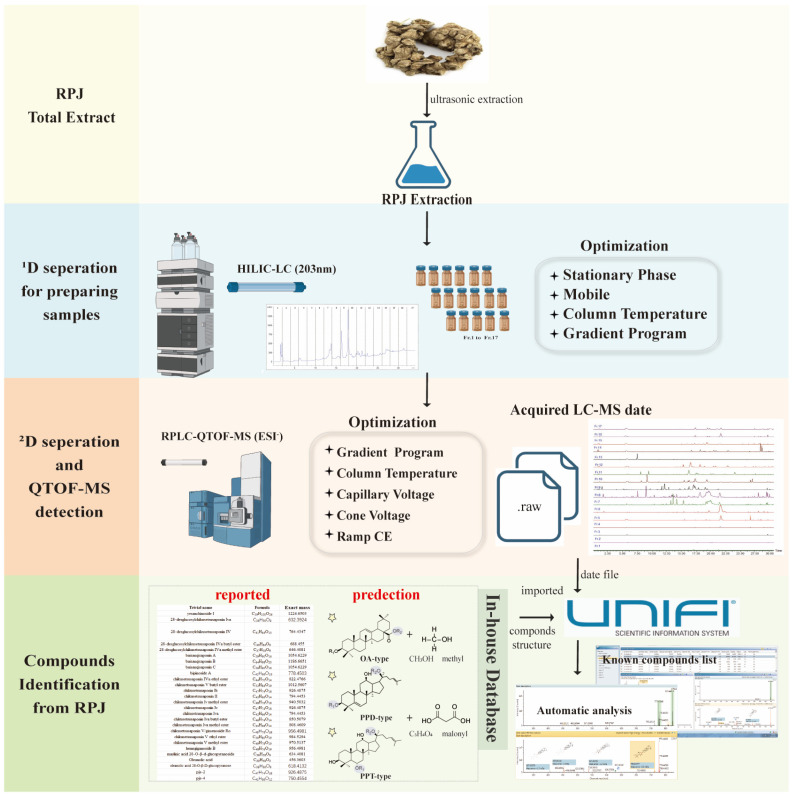
A comprehensive workflow for profiling triterpene saponins in Rhizoma Panacis Japonici.

**Figure 2:**
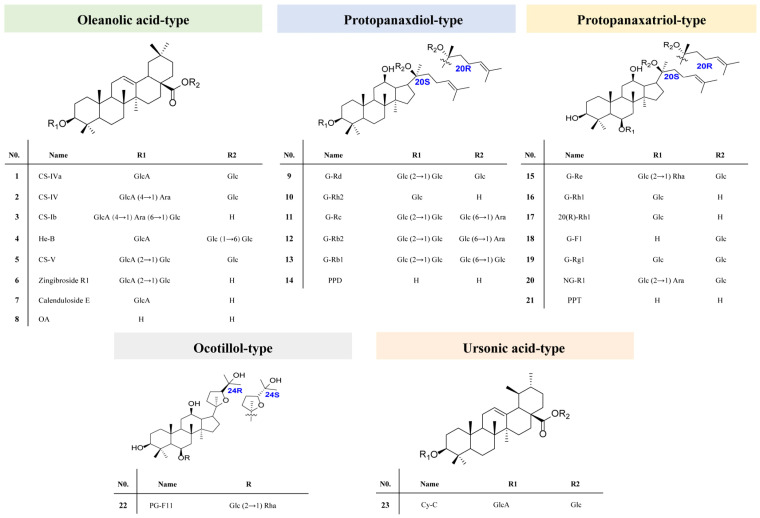
The structures of 23 reference saponins used in the study: G, Ginsenoside; NG, Notoginsenoside; CS, chikusetsusaponin; Cy-C, Cynarasaponin C; He-B, hemsgiganoside B; PG-F11, Pseudoginsenoside F11; Glc, glucose; Rha, rhamnose; Ara, arabinose; Xyl, xylose; GlcA, glucuronic acid.

To date, about 113 triterpene saponins have been isolated from RPJ, which can be categorized into the protopanaxdiol (PPD), protopanaxtriol (PPT), octillol (OT), oleanolic acid (OA), and ursonic acid (UA) types [29,30]. Unlike *P. ginseng* and *P. notoginseng*, which are well-known medicinal plants in the *P.* genus, RPJ is unique for its high content of OA-type saponins along with a small amount of dammarane-type saponins. In previous studies, RPJ has been analyzed using different methods, such as UHPLC-Q-Exactive Orbitrap HRMS and UFLC-Triple TOF-MS/MS, resulting in the characterization of 53 and 82 saponins, respectively [31,32]. Additionally, Chunxia Zhang et al. [15] expanded on this research by identifying 178 components in RPJ using RP LC/IM-QTOF-MS combined with data-dependent and data-independent acquisition strategies. However, there is still a gap in characterizing saponins of RPJ. To address this, we established an offline HILIC × RP LC/QTOF-MS system. Additionally, we developed a comprehensive in-house database that documents 612 saponins found in the *P.* genus and 228 predicted metabolites for characterizing saponins in RPJ. The workflow is illustrated in Figure 1. The RPJ was extracted and separated into multiple fractions using a HILIC column (^1^D HPLC). Each fraction was then further separated by a BEH-C18 column (^2^D UPLC) and detected using QTOF-MS/MS in negative mode. The mass data were efficiently managed, and the saponins present in RPJ were automatically identified with UNIFI software. Interpretations were made by studying the fragmentation behaviors of 23 reference saponins (Figure 2).

## 2. Results and Discussion

### 2.1. Optimization of the ^1^D and ^2^D-LC Conditions

To isolate saponins from RPJ, we selected combinations of HILIC × RP to isolate triterpene saponins of RPJ according to some reports [8,15]. The ^1^D (HILIC) and ^2^D (RP) LC conditions were optimized systematically. For ^1^D HPLC separations, we compared the separation performance of two stationary phases, including silica (Atlantis HILIC column) and amide (XBridge Amide column and BEH Amide column). As shown in Figure S1, columns packed with amide material performed better in retaining saponins of RPJ than the Atlantis HILIC column (silica). Compared with the BEH Amide column, more peaks with symmetrical peak shapes were observed when using the XBridge Amide column. Therefore, a XBridge Amide column was used for the ^1^D-LC isolation of RPJ extract. Given that RPJ primarily contains acidic saponins (OA type), we evaluated the impact of water and additive of 0.1% formic acid (FA), 0.1 M ammonium formate (AF), and 0.1% trifluoroacetic acid (TFA) on the separation of saponins in RPJ using acetonitrile (CH_3_CN) as the organic phase (Figure S2). Based on the number of peaks and peak symmetry, it was found that the addition of 0.1% formic acid yielded better results compared to 0.1 M AF and 0.1% TFA in separating the RPJ extract. Compared to using water alone, a greater number of peaks were observed within 19–23 min. Thus, 0.1% FA-CH_3_CN was used as the mobile phase. Furthermore, we compared the impact of varying column temperatures (25 °C–40 °C) on the separation of RPJ (Figure S3). The results showed that increasing the column temperature had a minimal impact on the resolution, so it was set at 30 °C.

For the ^2^D-LC isolation, the performance of five different RP columns was evaluated in isolating RPJ extract and 23 reference saponins using an UPLC-QTOF-MS instrument. Based on previous research on ginsenoside [22,32,33,34,35], Scepter C18-120, CORTECS C18, BEH Shield RP18, HSS T3, and BEH C18 columns were selected. As depicted in Figure S4, the RPJ extract exhibited the highest number of chromatography peaks with the Scepter C18-120 column (298 peaks), followed by the BEH C18 column (287 peaks) and CORTECS- C18 column (257 peaks). Compared with others, the BEH C18 column was able to completely separate the 23 reference saponins and exhibited the best resolution of neighboring peaks. Due to the specificity of saponins, the BEH C18 column was selected for the ^2^D UPLC separation. We then regulated the column temperatures and observed that enhancing the resolution of some minor peaks at 40 °C compared to 30 °C or 35 °C (Figure S5). Thus, the column temperature of the ^2^D UPLC isolation was set at 40 °C.

### 2.2. Optimization of QTOF-MS Parameters

**Figure 3:**
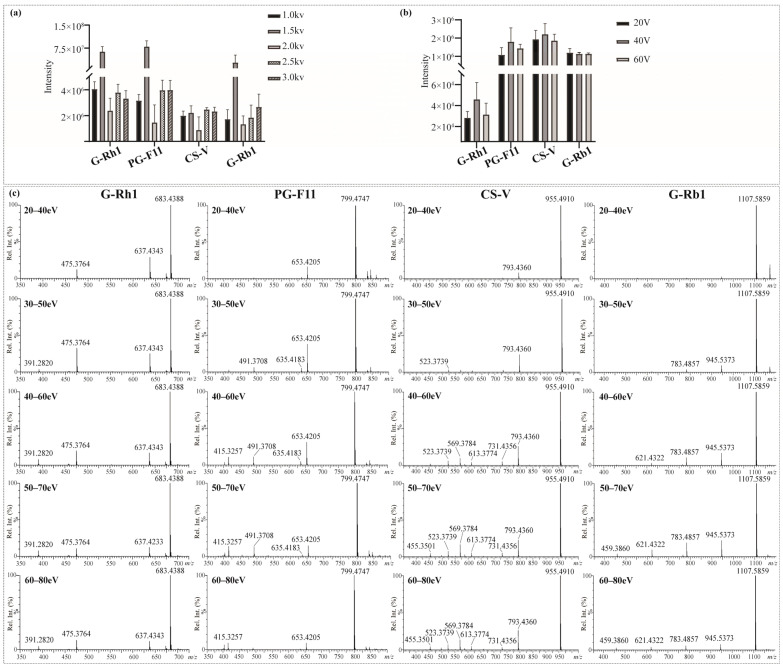
Influence of (**a**) capillary voltage, (**b**) cone voltage, and (**c**) collision energy on MS^2^ behaviors of the four representative saponins.

The key parameters of the Synapt^TM^ mass spectrometer (Waters, Milford, MA, USA) were fine-tuned to obtain maximum sensitivity and product ion information of saponins in RPJ. Firstly, both the positive and negative ion mode were utilized to analyze the reference saponins. Abundant fragments could be generated from the parent ion in negative ion mode as a result of consecutive neutral loss of the external sugar substituent. In contrast, adduct ions ([M + Na]^+^ and [M + NH_4_]^+^) were produced in positive ion mode, and fewer fragments were apparent in MS^2^ spectra. The results were consistent with the previously described [36]. Thus, we chose negative mode in this study. Next, the capillary voltage and cone voltage were tested by evaluating the intensity of four types of saponins, including chikusetsusaponin V (OA type), ginsenoside Rb1 (PPD type), ginsenoside Rh1 (PPT type) and Pseudoginsenoside F11 (OT type). For the capillary voltage (Figure 3a), the adduct ion intensity of target compounds was differently changed at 1.0–3.0 kV, but the ionization was relatively high at 1.5 kV. Thus, the capillary voltage was set at 1.5 kV. The cone voltage could induce in-source dissociation of saponins, and an optimal value would enable a higher response and detection sensitivity. All index saponins produced relatively high ionic intensity of [M − H]^−^ at 40 V, indicating that 40 V was the optimal cone voltage (Figure 3b). 

Collision energy (CE) is vital to induce dissociation of protonated or deprotonated parent ions [37]. In MS, deglycosylated fragments and sapogenin ions are the main diagnostic ions of saponins. Specifically, we selected G-Rh1, PG-F11, CS-V, and G-Rb1 as representative saponins with one to four sugar units, respectively. Optimization of CE was carried out within specific energy ranges: 20–40 eV, 30–50 eV, 40–60 eV, 50–70 eV, and 60–80 eV. As shown in Figure 3c. The energy required to generate sapogenin ions increased with the number of glycosidic bonds to some extent. G-Rh1, a monoglycoside, produced relatively high levels of *m*/*z* 475.3764 [sapogenin − H]^−^ and its fragments *m*/*z* 391.2820 at lower energy (CE 30–50 eV). The optimal CE for PG-F11 (diglycoside) and CS-V (triglycoside) was 40–60 eV and 50–70 eV, respectively. In contrast, G-Rb1 (tetraglycoside) generated *m*/*z* 475.3764 [sapogenin − H]^−^ at higher collision energies of 50–70 eV and 60–80 eV. Notably, at CE 50–70 eV, the mass spectra of the four saponins showed a variety of fragments and relatively high levels of aglycone ions. Therefore, the collision energy was set at 50–70 eV in the MS measurement of the sample.

### 2.3. Evaluation and Method Validation

**Figure 4:**
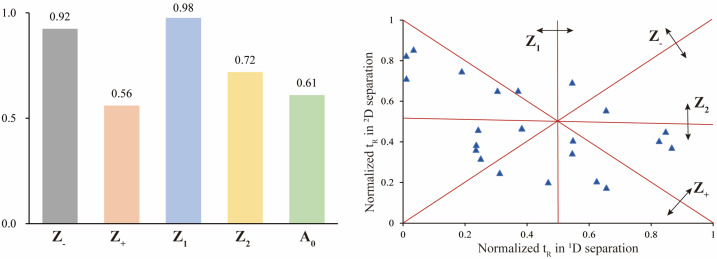
Orthogonality of the offline HILIC × RP LC system with asterisk equations using 23 reference saponins.

**Figure 5:**
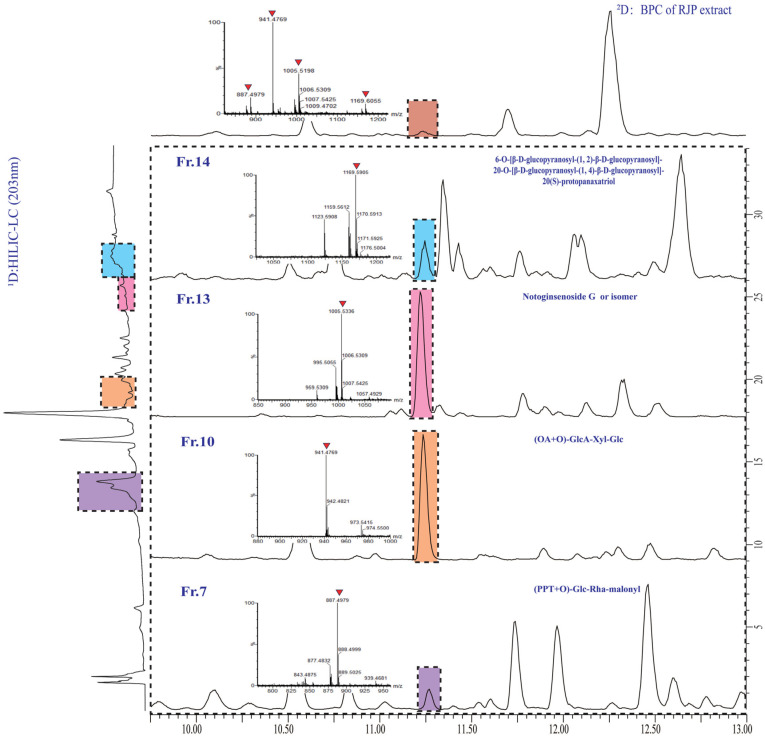
Enlarged ^1^D HPLC-UV (203 nm)-^2^D base peak ion (BPI) chromatograms of RPJ extract and selected fractions, showing the four coeluted saponins in RPJ extract were separated and detected in fractions 14, 13, 10, and 7.

The assessment of the offline 2D LC system’s separation performance involved orthogonality and peak capacity. By applying asterisk Equations [38], the spreading of 23 reference saponins was calculated, resulting in an orthogonality value of 0.61 (Figure 4). The parameters of the four crossing lines were calculated at 0.92 (Z_−_), 0.56 (Z_+_), 0.98 (Z_1_) and 0.72 (Z_2_), respectively. The ^1^D and ^2^D-LC exhibited a peak capacity of 97 (mean peak width 0.33 min) and 135 (mean peak width 0.27 min), respectively. Consequently, the 2D LC system exhibited a theoretical peak capacity of 13,175, with an effective peak capacity of 1249. These results indicated that the developed offline HILIC × RP LC system significantly enhanced the resolution of saponins in RPJ. For example (Figure 5), the chromatographic peak at t_R_ 11.23 min of RPJ extract detected by RP-LC/QTOF-MS potentially contained four coeluted compounds (*m*/*z* 1169.6055, *m*/*z* 1005.5336, *m*/*z* 941.4769, *m*/*z* 887.4979). Characterizing their structures based on MS^2^ data was challenging due to the mixed fragment ions of all the coeluted compounds. However, the developed 2D LC-MS system successfully distributed these saponins in Fr.14, 13, 10 and 7, and identified as 6-O-[β-d-glucopyranosyl-(1,2)-β-d-glucopyranosyl]-20-O-[β-d-glucopyranosyl-(1,4)-β-d-glucopyranosyl]-20(S)-protopanaxatriol or isomer, notoginsenoside G or isomer, (OA+O)-GlcA-Xyl-Glc and (PPT+O)-Glc-Rha-malonyl. This result highlights the significant advantage of the offline 2D LC-MS system in resolving coeluted and trace components in samples.

Additionally, simplified method validation was conducted for both ^1^D and ^2^D separations as the reports [33,39], including repeatability, inter-/intra-day precision and limit of detection (LOD). Five index saponins (including G-Rh1, G-Re, CS-V, CS-IVa and CS-IV) were used to evaluate precision and repeatability. The relative standard deviation (RSD, %) for inter-/intra-day precision of ^1^D and ^2^D separation ranged from 0.52% to 4.03% and from 0.70% to 6.43% (Tables S1–S4), respectively. For repeatability of the offline 2D LC-MS method, the RSD of the five saponins ranged from 1.11% to 3.87% (Table S5). The LOD of G-Rh1, G-Re, CS-V, CS-IVa and CS-IV were 1.19 ng, 1.50 ng, 1.23 ng, 2.40 ng, and 1.20 ng, respectively. The findings suggested that the devised method of HILIC × RPLC/QTOF-MS is stable, sensitive and repeatable.

### 2.4. Systematic Characterization of the Triterpene Saponins in RPJ

The triterpenoid saponins of RPJ mainly consist of OA and dammarane types, which can be categorized into PPD, PPT, OT type, and varied C17 side chains. Regarding the sugar constituents, GlcA (C_6_H_10_O_7_), Glc (C_6_H_12_O_6_), Rha (C_6_H_12_O_5_), Ara (C_5_H_10_O_5_) and Xyl (C_5_H_10_O_5_) have been reported in *P.* species [8,36], showing the neutral loss of 176.0319 Da, 162.0550 Da, 146.0542 Da, 132.0365 Da and 132.0365 Da, respectively. In the present study, Xyl was used to address pentose residue for the neutral loss of 132.0365 Da. Furthermore, esterified or acylated saponins were also characterized in RPJ (Table S8).

#### 2.4.1. OA-Type Saponins

**Figure 6:**
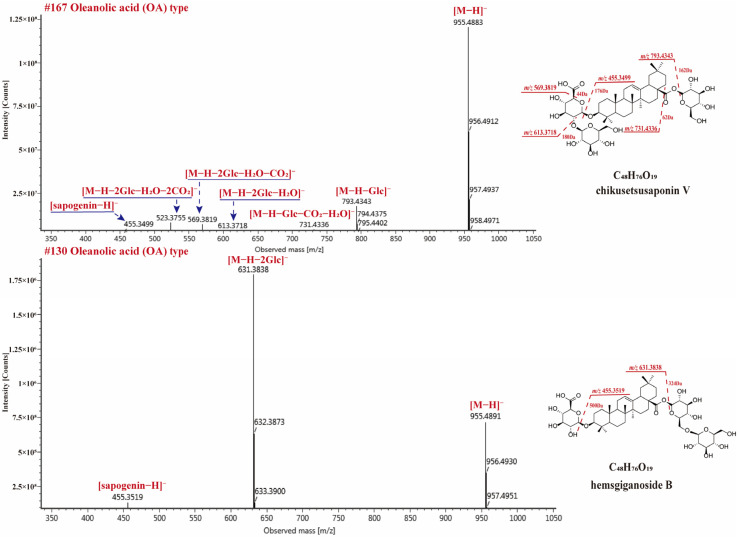
The MS^2^ spectra for chikusetsusaponin V and hemsgiganoside B in negative ion mode.

150 OA-type saponins in RPJ were identified in this study. The major diagnostic fragments for these saponins were the dehydrogenated aglycone ion at *m*/*z* 455.3501 and the neutral loss of 43.9990 Da (CO_2_). The sugar chains of OA-type saponins are typically attached at positions 28-COOH and/or 3-OH. It is observed that the glycoside linkage at C-28 is more susceptible to breakage compared to the one at the C-3 position in the negative ion mode. Thus, the substitution positions or isomers of various sugar chains in OA-type saponins can be determined by analyzing the relative abundance of deglycosylated fragments. For example, CS-V and He-B are isomers with two sugar chains. They both produced [M − H]^−^ ion at *m*/*z* 955.4883 (C_48_H_76_O_19_) and deprotonated sapogenin ion at *m*/*z* 455.3519 (Figure 6). CS-V (3-GlcA-Glc, 28-Glc) generated deglucose chain fragments at *m*/*z* 793.4343 ([M − H − Glc]^−^), along with ions at *m*/*z* 731.4336 ([M − H − Glc − H_2_O − CO_2_]^−^), 613.3718 ([M − H − H_2_O − 2Glc]^−^), and 569.3819 ([M − H − 2Glc − CO_2_ − H_2_O]^−^). On the other hand, He-B produced a high-intensity [M − H − 2Glc]^−^ ion at *m*/*z* 631.3838, indicating that two glucose molecules are linked to the C-28 position instead of C-3. Similar cleavage behaviors were also observed in compounds CS-IV and CS-Ib, which is consistent with a previous report [40]. 

It is worth noting that *P. japonicus* also contains UA-type saponins, which are aglycone isomers of the OA type, such as Cynarasaponin (Cy-C, UA-28-Glc-3-GlcA) and CS-IVa (OA-28-Glc-3-GlcA) [41]. They were distinguished by the retention time (CS-IVa, t_R_, 21.72 min; Cy-C, t_R_, 22.15 min) provided by the reference substances because of their highly similar MS^2^ spectra. So, the sapogenin ion at *m*/*z* 455.3534 observed in MS^2^ spectra was identified as the OA type. In MS/MS qualitative characterization, identifying high-level isomers has always been a challenging task especially in the absence of reference compounds. In recent years, techniques like energy-resolved (ER) MS and Ion Mobility (IM) MS have been utilized to distinguish isomers by detecting additional structural information, like optimal collision energy, half response collision energy, ion migration time, collision cross-section, and others. These techniques have been utilized for identifying isomers in HM without the need for reference compounds, such as lignan glycosides [39] and coumarins [42], among others. To our knowledge, the application of these techniques to distinguish UA and OA isomers has not yet been reported, which needs for further investigation.

#### 2.4.2. Dammarane Type Saponins

**Figure 7:**
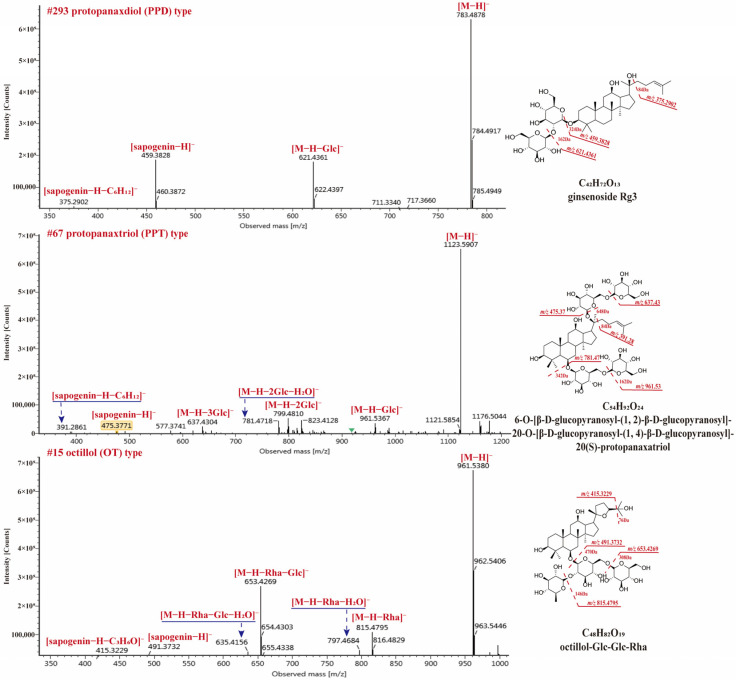
The MS^2^ spectra for three dammarane-type saponins in negative ion mode.

PPT and PPD types are the main dammarane-type saponins in *P.* species. 31 PPD-type and 48 PPT-type saponins have been identified from RPJ in this study and they commonly produced [M − H]^−^ and/or [M + HCOO]^−^ in negative ion mode. The major diagnostic fragments of PPT and PPD-type were observed at *m*/*z* 475.3764 (C_30_H_51_O_4_) → 391.2820 and 459.3860 (C_30_H_51_O_3_) → 375.2902, respectively. Compound **67** (t_R_, 11.26 min) gave [M + HCOO]^−^ and [M − H]^−^ ions at *m*/*z* 1169.5959 and 1123.5907, respectively, corresponding to the formula C_54_H_92_O_24_ (Figure 7). The [M − H]^−^ ion generated diagnostic fragments at *m*/*z* 475.3771 ([M − H − 4Glc]^−^) and 391.2861 ([M − H − 4Glc − C_6_H_12_]^−^), along with *m*/*z* 961.5367 ([M − H − Glc]^−^), 799.4810 [M − H − 2Glc]^−^, 781.4718 ([M − H − 2Glc − H_2_O]^−^) and 637.4304 ([M − H − 3Glc]^−^) ions. So, Compound **67** was characterized as 6-O-[β-d-glucopyranosyl-(1,2)-β-d-glucopyranosyl]-20-O-[β-d-glucopyranosyl-(1,4)-β-d-glucopyranosyl]-20(S)-protopanaxatriol. Compound **293** (t_R_, 28.44 min), the molecular formula C_42_H_72_O_13_ was confirmed by *m*/*z* 829.4951 ([M + HCOO]^−^) and 783.4878 ([M − H]^−^). The parent ion (*m*/*z* 783.4878) lost two Glc residues and gave *m*/*z* 459.3828 ([sapogenin − H]^−^) and 375.2902 ([sapogenin − H − C_6_H_12_]^−^) ions (Figure 7). Its molecular formula and cleavage behavior were consistent with ginsenoside Rg3 [43]. In addition, 16 OT-type saponins have been identified from RPJ and their characteristic ions were at *m*/*z* 491.3708 and 415.3257 in negative ion mode. Compound **15** (t_R_, 5.47 min) showed abundant [M + HCOO]^−^ and [M − H]^−^ ions at *m*/*z* 1007.5425and 961.5380, corresponding to the formula C_48_H_82_O_19_ (Figure 7). Fragment ions at *m*/*z* 815.4795 ([M − H − Rha]^−^), 797.4684 ([M − H − Rha − H_2_O]^−^), 653.4269 ([M − H − Rha − Glc]^−^), 635.4156 ([M − H − Rha − Glc − H_2_O]^−^), 491.3732 ([M − H − Rha − 2Glc]^−^), and 415.3229 ([M − H − Rha − 2Glc − C_3_H_6_O]^−^) were observed in MS^2^ spectra. Thus, compound **15** was identified as octillol-Glc-Glc-Rha.

PPD or PPT-type saponins with dehydrated on sapogenin and varied C17 side chains have been reported from *P.* genus [29,44]. In the present study, 19 and 38 compounds of these types were characterized, which usually yield [sapogenin − H]^−^ ion and specific fragments of the C17 side chain. For example, Compound **284** (t_R_, 28.04 min) gave abundant ions at *m*/*z* 827.4825 ([M + HCOO]^−^) and 781.4734 ([M − H]^−^), which are consistent with the formula C_42_H_70_O_13_. Its [M − H]^−^ ion yielded fragments of *m*/*z* 619.4333 ([M − H − Glc]^−^), 457.3685 ([M − H − 2Glc]^−^) and 373.2753 ([M − H − 2Glc − C_6_H_12_]^−^). The neutral losses of the sugar chain and C_6_H_12_ indicated that compound **284** was ginsenoside 5-ene-PPD-Glc-Glc. Compound **31** (t_R_, 7.78 min) was firstly identified in RPJ. It yielded *m*/*z* 861.4861 ([M + HCOO]^−^) and 815.4783 ([M − H]^−^) ions, indicating the formula C_42_H_72_O_15_. The fragments *m*/*z* 669.4212, 507.3681, and 491.3747 were assigned to [M − H − Rha]^−^, [M − H − Rha − Glc]^−^, and [M − H − Rha − Glc − H_2_O]^−^ ions, respectively. The ion at *m*/*z* 507.3681 suggested two methyl substitutions in the side-chain of the aglycone (PPT). As reported in the literature [45], compound **31** was identified as floralquinquenoside B. Similar fragmentation behavior was also observed in compound **10** (C_41_H_72_O_15_), which generated ions at *m*/*z* 803.4777 ([M − H]^−^), 671.4326 ([M − H − Xyl]^−^), 509.3869 ([M − H − Xyl − Glc]^−^), and 391.2929 ([M − H − Xyl − Glc − C_6_H_14_O_2_]^−^). The fragments at *m*/*z* 509.3869 and *m*/*z* 391.2929 indicated that its aglycone was 23,24-OH-PPT. Therefore, compound **10** was characterized as 23,24-OH-PPT-Glc-Xyl.

#### 2.4.3. Esterified and Acylated Type Saponins

**Figure 8:**
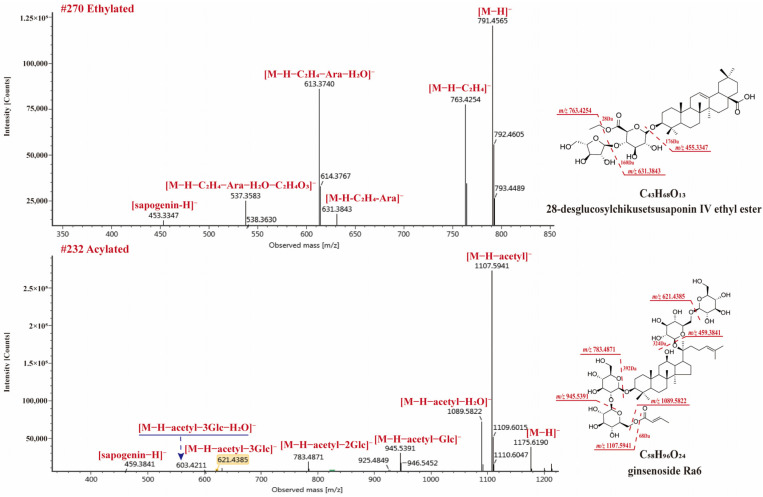
The MS^2^ spectra for ethylated and acetylated saponins in negative ion mode.

Esterified and acylated saponins were also observed in RPJ. In the case of OA-type saponins, esterification could occur due to the presence of free carboxyl in sapogenin or glucuronic acid [30]. Among the OA-type saponins, 21 formylated, 7 ethylated, 1 polyacetylene (compound **269**), and 1 acylated (compound **255**) were found. The neutral losses of 13.9753 Da (methyl), 28.0311 Da (ethyl), 42.0113 Da (acetyl), 44.0104 Da (malonyl) could be observed in negative ion mode, respectively. Compound **270** (t_R_, 27.36 min) gave [M + HCOO]^−^ and [M − H]^−^ ions at *m*/*z* 837.5613 and 791.4565, respectively, corresponding to the formula C_43_H_68_O_13_ (Figure 8). The ion at *m*/*z* 763.4254 ([M − H − C_2_H_4_]^−^), indicating the carboxyl was esterified. The precursor ion further generated *m*/*z* 631.3843 ([M − H − C_2_H_4_ − Ara]^−^), 613.3740 ([M − H − C_2_H_4_ − Ara − H_2_O]^−^), 537.3583 ([M − H − C_2_H_4_ − Ara − C_2_H_4_O_3_]^−^), and 455.3347 ([M − H − C_2_H_4_ − Ara − GlcA]^−^). Thus, compound **270** was identified as 28-desglucosylchikusetsusaponin IV ethyl ester.

On the other hand, dammarane-type saponins containing acetyl/malonyl substituents are also reported from *P.* species [8,36], and these substituents are usually at C20-sugar chain. We also found 2 malonylated and 16 acetylated saponins from RPJ in this study. In negative ion mode, these saponins exhibited [M − H − acetyl]^−^ and [M − H − acetyl − H_2_O]^−^ diagnostic fragments, consistent with previous reports [36]. Compound 232 (t_R_, 25.75 min, C_56_H_94_O_24_) showed [M + HCOO]^−^ and [M − H]^−^ ions at *m*/*z* 1221.6252 and 1175.6190, respectively. In MS^2^, the high intensity of ions at *m*/*z* 1107.5941 [M − H − acetyl]^−^ and 1089.5822 [M − H − acetyl − H_2_O]^−^ were observed, accompany with fragments at *m*/*z* 945.5391 [M − H − acetyl − Glc]^−^, 783.4871 [M − H − acetyl − 2Glc]^−^, 621.4385 [M − H − acetyl − 3Glc]^−^, 603.4211 [M − H − acetyl − 3Glc − H_2_O]^−^, and 459.3841 [M − H − acetyl − 4Glc]^−^. Compound 232 was identified as ginsenoside Ra6.

In this study, 307 saponins were identified from RPJ using the developed 2D LC-QTOF-MS method based on an in-house database of *P.* genus. Among these saponins, 150 were categorized as OA-type saponins and their derivatives, while one was classified as a UA-type saponin. Furthermore, 156 dammarane-type saponins were identified, including C17 side-chain varied compounds along with esterified and acylated derivatives. In the chemical characterization of HM using 1D or 2D LC-HRMS, a comprehensive and specific database is necessary to enhance the efficiency of compound identification and ensure consistent results. Nonetheless, relying solely on computer-aided database searches may limit the exploration of new compounds to some extent.

## 3. Materials and Methods

### 3.1. Chemicals and Reagents

A total of 23 reference saponins were used in this study (Figure 2). Reference chikusetsusaponin-IVa, -IV, -Ib, -V, ginsenoside -Rd, -Rh2, -Re, -Rc, -Rb1, -Rb2, -F1, -Rg1, 20 (S) ginsenoside-Rh1, 20 (R) ginsenoside-Rh1, notoginsenoside-R1, zingibroside R1, Calenduloside E, oleanolic acid, protopanaxadiol, and protopanaxatriol were, respectively, purchased from Herbest Bio-Tech Co., Ltd. (Baoji, China), DeSiTe Biological Technology Co., Ltd. (Chengdu, China), Biopurify Phytochemicals Ltd. (Chengdu, China) and Pufei De Biotech Co., Ltd. (Chengdu, China) Hemsgiganoside B and cynarasaponin C were isolated from RPJ in our lab [41]. LC-grade acetonitrile and methanol were provided by Fisher Co. Ltd. (Emerson, IA, USA). The analytical grade trifluoroacetic acid, formic acid, and ammonium formate were purchased from Macklin Biotech Co., Ltd. (Shanghai, China). For the preparation of ultrapure water, a Milli-Q Reagent Water System (Millipore, Bedford, MA, USA) was utilized. The Rhizoma of *Panacis Japonici* was obtained from a pharmacy in Chengdu. It was identified as the rhizome of Panax japonicus C. A. Mey. by Associate Professor Li Jia of the College of Traditional Chinese Medicine at Capital Medical University. Voucher specimens with Batch No. PJ201901 have been deposited at the authors’ lab in Capital Medical University (Beijing, China).

The columns used were as follows: XBridge Amide column (4.6 × 150 mm, 3.5 μm, Waters, USA) and (2.1 × 150 mm, 3.5 μm, Waters, USA), Atlantis HILIC silica column (2.1 × 150 mm, 3 μm, Waters, USA), BEH Amide column (2.1 × 100 mm, 1.7 μm, Waters), BEH C18 column (2.1 × 100 mm, 1.7 μm, Waters, USA), BEH Shield RP18 column (2.1 × 100 mm, 1.7 μm, Waters, USA), ACQUITY UPLC HSS T3 column (2.1 × 100 mm, 1.8 μm, Waters, USA), CORTECS UPLC C18 column (2.1 × 100 mm, 1.6 μm, Waters, USA) and Shim-pack Scepter C18-120 (2.1 × 150 mm, 1.9 μm, Shimadzu, Kyoto, Japan).

### 3.2. Sample Preparation

Ten g of fine powder of RPJ was extracted with the assistance of ultrasound (100 W, 50 Hz) for one hour at 30 °C using 200 mL of 70% methanol as solvent. The mixture was then centrifuged at 3000 rpm for 15 min. The supernatant liquid was filtered through a 0.22 μm PTFE filter membrane and stored at 4 °C for analysis.

### 3.3. Offline HILIC×RP LC/QTOF-MS Conditions

The first-dimensional (^1^D) separation was performed on an Agilent 1200 HPLC system (Agilent Technologies, Santa Clara, CA, USA) using a Waters XBridge Amide column (4.6 × 150 mm, 3.5 μm). The mobile phase consisted of 0.1% FA (*v*/*v*) in water (A) and acetonitrile (CH_3_CN, B), with the liner elution gradient: 0–3 min, 95% B; 3–5 min, 95–90% B; 5–14 min, 90–85% B; 14–17 min, 85% B; 17–22 min, 85–83% B; 22–27 min, 83–60% B; 27–32 min, 60% B; 32–35 min, 60–95% B. The column temperature was set at 30 °C and the wavelength was at 203 nm. The flow rate was 1.0 mL/min and the injection volume were 20 μL. Seventeen fractions (Fr.1–Fr.17) were collected every 2 min from 1 to 34 min with six replications. The fractions were dried with a steady flow of N_2_ at room temperature. Each residue was then redissolved in 100 μL of 70% methanol followed by centrifugation at 14,000 rpm for 10 min, and the supernatant was retained for the second-dimensional (^2^D)-RPLC separation.

The ^2^D-RPLC separation was conducted on an UPLC Acquity™ system (Waters, USA) utilizing a Waters Acquity UPLC BEH C18 column (2.1 × 100mm, 1.7 μm). The mobile phase was composed of 0.1% formic acid (*v*/*v*) in water (A) and CH_3_CN (B), with the column temperature at 40 °C. The elution gradient was as follows: 0–4 min, 90–80% A; 4–9 min, 80–77% A; 9–10 min, 77–71% A; 10–12 min, 71–70% A; 12–22 min, 70–68% A; 22–24 min, 68–66% A; 24–24.5 min, 66–58% A; 24.5–36.5 min, 58–15% A; 36.5–37.5 min, 15–5% A; 37.5–40.5 min, 5% A. Five μL were injected and the flow rate was 0.3 mL/min.

Acquisition was performed in MS^E^ mode with a Synapt™ QTOF high-resolution mass spectrometer (Waters, USA) under negative ion mode. The optimized parameters for mass detection were as follows: high-purity nitrogen (N_2_) was used as desolvation gas (800 L/h) and nebulizer gas (40 psi); the desolvation temperature was 450 °C. The cone gas flow was set at 50 L/h, capillary voltage at 1.5 kV, cone voltage at 40 V, supplemental ion source voltage at 80V, ion source temperature at 120 °C, low collision energy at 6 eV, and high collision energy ranging from 50 to 70 eV. The mass scan range was *m*/*z* 350–1500. Real-time calibration was performed using leucine enkephalin (400 ng/mL) at a flow rate of 10 μL/min. 

### 3.4. Evaluation of Orthogonality and Peak Capacity

Orthogonality and peak capacity of the developed 2D LC system were calculated with a set of asterisk formula [38,46] (Supplementary Formula (S1)). The normalized retention time (t_I_) of each reference component to the relative retention time (t’_R_, norm(i)) based on equation (Equation (S1)) (t_D_: dead volume time; t_G_: effective elution time of the chromatography system). The peak distribution around the four lines Sz_−_, Sz_+_, Sz_1_, and Sz_2_ were according to Equations (S2)–(S5) (σ: standard deviation of the values of all 23 index components). The Z parameters were calculated according to Equations (S6)–(S10), which yields the orthogonality result A_0_. The peak capacity of theoretical (n_c,2D_) and effective (n’_c,2D_) were determined based on Equations (S11)–(S13), in which W_b_ represents the average peak widths of three well-separated chromatographic peaks at the beginning, middle, and end of the elution gradient.

### 3.5. Development of an In-House Database of P. genus

To comprehensively characterize triterpene saponins in RPJ, an in-house database of *P.* genus was established, including 612 saponins reported in the genus and 228 predicted metabolites (Tables S6 and S7). The database includes an Excel file with records of 840 saponins’ names, formulas, theoretical molecular weights, and MS/MS characteristics, as well as a .mol file for each compound. The information on these saponins was primarily obtained through literature research. Chemical structures with incomplete information were obtained by retrieved from online databases such as PubChem (https://pubchem.ncbi.nlm.nih.gov/, accessed on 29 January 2024), ChemSpider (https://www.chemspider.com/, accessed on 29 January 2024) and Chemicalbook (https://www.chemicalbook.com/ProductIndex.aspx, accessed on 29 January 2024), or drawn using KingDraw 3.0 software. MS/MS fragments were acquired by searching online databases, including Massbank (https://massbank.jp, accessed on 6 March 2024), HMDB (https://hmdb.ca, accessed on 6 March 2024), etc.

Previous studies have demonstrated that OA-type saponins in the *P.* genus can undergo esterification with methyl, ethyl, and butyl groups [30], whereas dammarane-type ginsenosides may exhibit substitutions of malonyl and acetyl [22,36]. Consequently, potential structures of the saponins reported in PRJ were predicted, encompassing 47 OA-type saponin derivatives and 181 dammarane-type saponin derivatives. For predicted compounds, molecular weights were calculated using MassLynx 4.1 workstation and MS/MS fragments were inferred based on similar saponins.

### 3.6. Method Validation

Validation of the established HILIC × RP LC/QTOF-MS method was conducted for inter-/intraday precision, reproducibility and LOD using five reference saponins (G-Rh1, G-Re, CS-V, IVa and CS-IV) as index compounds. To evaluate the inter-/intraday precision of ^1^D and ^2^D separation, six repeated injections were performed on the first day, followed by three consecutive injections on the second and third days. Precision and reproducibility were assessed using the relative standard deviation (RSD, %). The LOD of the four reference saponins (G-Rh1, G-Re, CS-V, IVa and CS-IV) were determined at a signal-to-noise(S/N) ratio of about 3.

### 3.7. Automated Peak Annotation with UNIFI

MS^E^ data were recorded using Masslynx and then processed with UNIFI 1.8.2 software, which employed a three-dimensional peak apex track integration algorithm to detect the full-scan data and provide clear low and high-energy spectra [17]. UNIFI facilitated data correction, matching of precursor and product ions, and peak annotation based on an in-house database imported into the software. The parameters of automatic annotation were as follows: low-energy and high-energy ion intensity thresholds were set at 300 and 40 counts, respectively, and target match tolerance and fragment were set at 10.0 ppm. The adduct ions [M − H]^−^ and [M + HCOO]^−^ were used to automatically screen for target components. Following processing, the software generated a list of ‘Identified Components’. To ensure accurate identification, a filter was applied with Detector Counts of ≥5000 and an error range of ppm ≤10.0 to reduce errors and false positives. The compounds listed under ‘Unknown Components’ were analyzed manually.

## 4. Conclusions

In this study, a sensitive and reliable offline HILIC × RP LC/QTOF-MS method was developed, along with an in-house database and structure prediction strategy. The method was successfully applied to characterize triterpene saponins from RPJ, demonstrating high orthogonality and peak capacity. A total of 307 saponins were identified from RPJ, with 76 of these saponins being identified for the first time in *P. japonicus*. These findings not only provide a deeper understanding of the chemical constituents of RPJ, but also offer a simple and effective approach for analyzing the complex composition of herbal medicine.

## Data Availability

The data presented in this study are available in article and Supplementary Materials.

## Supplementary Materials

The following supporting information can be downloaded at: https://www.mdpi.com/article/10.3390/molecules29061295/s1, Figure S1. ^1^D HILIC chromatograms (203 nm) of RPJ extract with different columns: (a) Atlantis HILIC silica column (2.1 × 150 mm, 3 μm), (b) BEH Amide column (2.1 × 100 mm, 1.7 μm), and (c) Xbridge Amide column (2.1 × 150 mm, 3.5 μm); Figure S2. ^1^D HILIC chromatograms (203 nm) of RPJ extract with different additives in the mobile phase: (a) Water; (b) 0.1% trifluoroacetic acid (TFA); (c) 0.1 M ammonium formate (AF); (d) 0.1% formic acid (FA); Figure S3. ^1^D HILIC chromatograms (203 nm) of RPJ extract with XBridge Amide column at different column temperature: (a) 25 °C, (b) 30 °C, (c) 35 °C and (d) 40 °C; Figure S4. Base peak ion (BPI) chromatograms of 23 reference saponins (left) and RPJ extract (right) with different RP-UPLC columns at 40 °C. (a) Scepter C18-120 (2.1 × 150 mm, 1.9 μm); (b) CORTECS C18 column (2.1 × 100 mm, 1.6 μm); (c) BEH C18 column (2.1 × 100 mm, 1.7 μm); (d) BEH Shield RP18 column (2.1 × 100 mm, 1.7 μm); (e) HSS T3 column (2.1 × 100 mm, 1.8 μm); Figure S5. Base peak ion (BPI) chromatograms of 23 reference saponins (left) and RPJ (right) extract with BEH C18 column at different column temperature: (a) 30 °C, (b) 35 °C and (c) 40 °C; Formula (S1). Asterisk equations for calculating orthogonality and peak capacity of 2D LC system; Table S1. Intra-day precision of the ^1^D separation (*n* = 6); Table S2. Inter-day precision of the ^1^D separation (*n* = 3); Table S3. Intra-day precision of the ^2^D separation (*n* = 6); Table S4. Inter-day precision of the ^2^D separation (*n* = 3); Table S5. Repeatability of the offline 2D LC-MS system (*n* = 6); Table S6. Information of the 612 saponins reported from the *Panax* genus as of 2023; Table S7. 228 predicted saponin metabolites of *Panacis Japonici*; Table S8. Detailed structural information of the 307 saponins characterized by 2D LC-MS.
