# Supplementary material for: Comprehensive Characterization of Triterpene Saponins in Rhizoma Panacis Japonici by Offline Two-Dimensional Liquid Chromatography Coupled to Quadrupole Time-of-Flight Mass Spectrometry
Source: Molecules. 2024 Mar 14;29(6):1295. doi: 10.3390/molecules29061295 (PMC10974181; doi:10.3390/molecules29061295)
Supplement: Supplementary file 1 [file molecules-29-01295-s001.zip › molecules-2872196-supplementary.pdf]

Figure legend

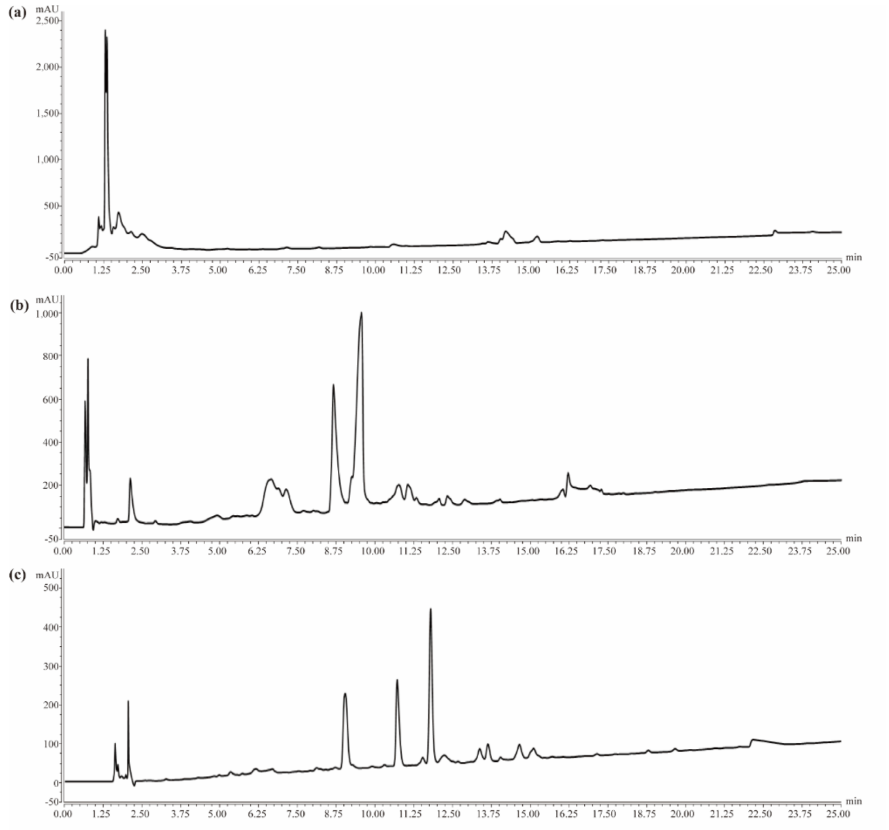

Figure S1. 1D HILIC chromatograms (203 nm) of RPJ extract with different columns: (a) Atlantis HILIC silica column (2.1 × 150 mm, 3 μm), (b) BEH Amide column (2.1 × 100 mm, 1.7 μm), and (c) Xbridge Amide column (2.1 × 150 mm, 3.5 μm).

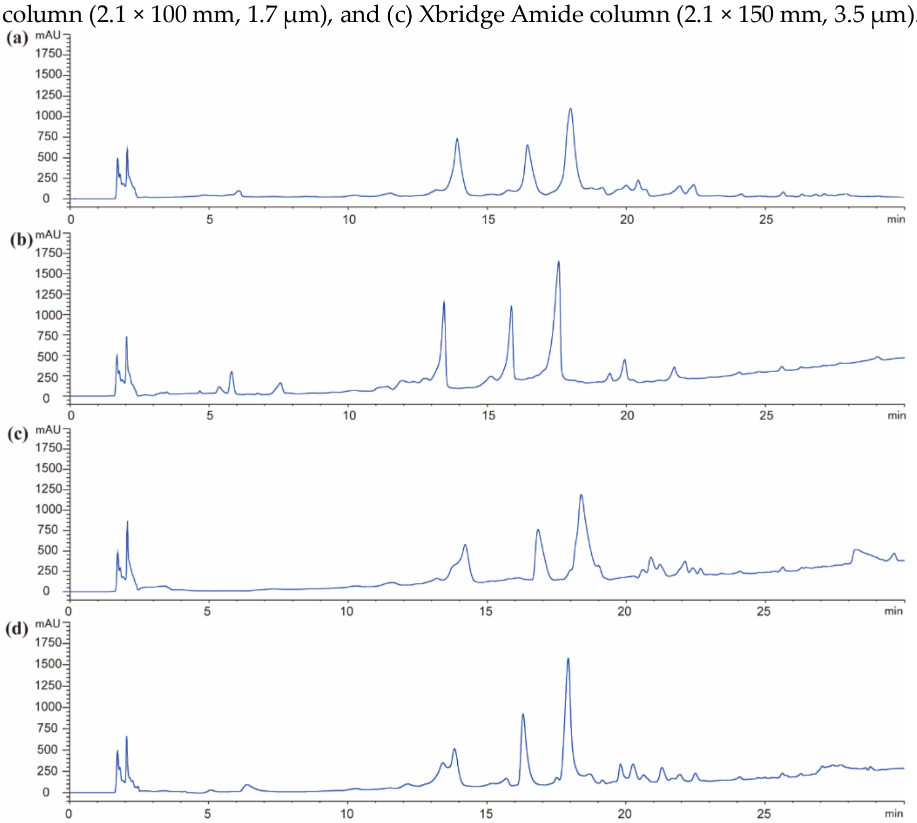

Figure S2. 1D HILIC chromatograms (203 nm) of RPJ extract with different additives in the mobile phase: (a) Water; (b) 0.1% trifluoroacetic acid (TFA); (c) 0.1 M ammonium formate (AF); (d) 0.1% formic acid (FA).

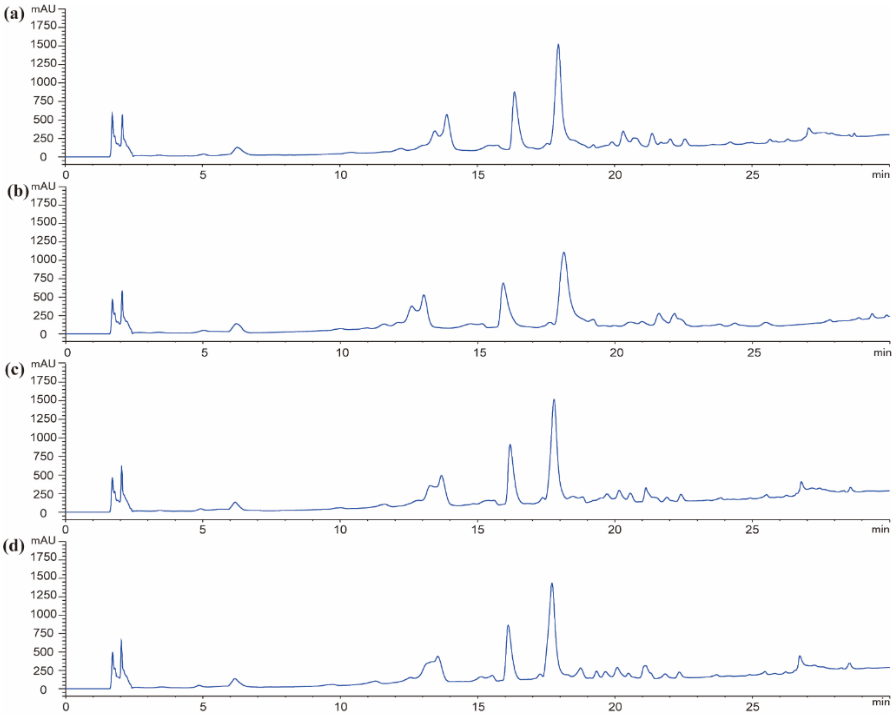

Figure S3. 1D HILIC chromatograms (203 nm) of RPJ extract with XBridge Amide column at different column temperature: (a) 25 °C, (b) 30 °C, (c) 35 °C and (d) 40 °C.

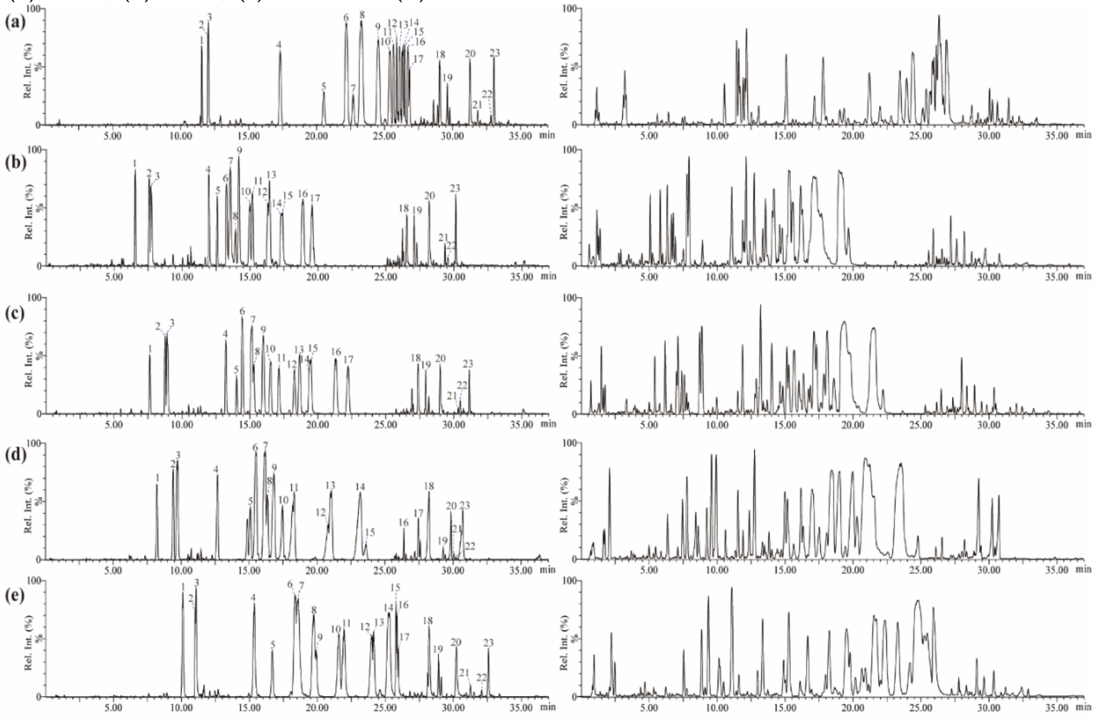

Figure S4. Base peak ion (BPI) chromatograms of 23 reference saponins (left) and RPJ extract (right) with different RP-UPLC columns at 40 °C. (a) Scepter C18-120 (2.1 × 150 mm, 1.9 µm); (b) COR-TECS C18 column (2.1 × 100 mm, 1.6 µm); (c) BEH C18

column (2.1 × 100 mm, 1.7 μm); (d) BEH Shield RP18 column (2.1 × 100 mm, 1.7 μm); (e) HSS T3 column (2.1 × 100 mm, 1.8 μm).

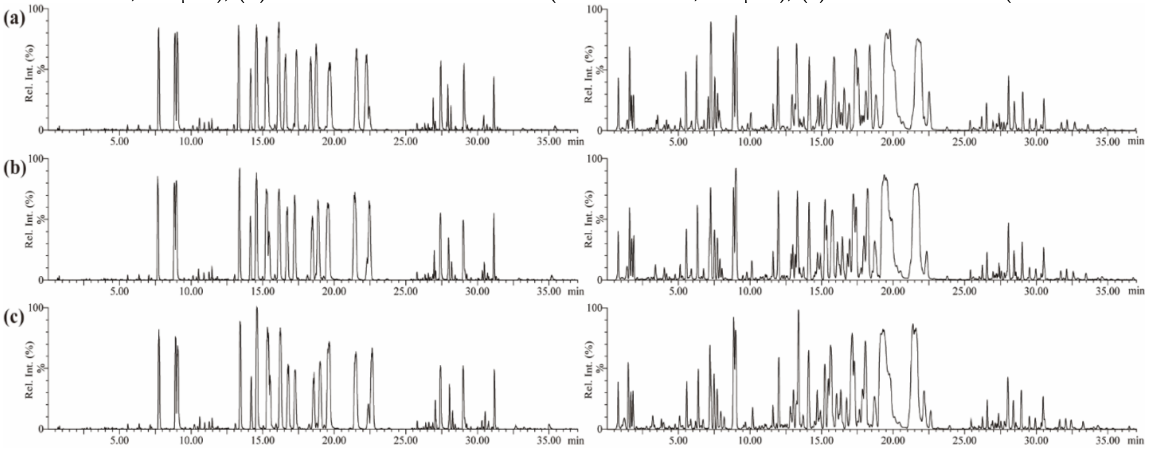

Figure S5. Base peak ion (BPI) chromatograms of 23 reference saponins (left) and RPJ (right) extract with BEH C18 column at different column temperature: (a) 30 °C, (b) 35 °C and (c) 40 °C.

$$t'_{R,norm(i)} = \frac{t_I - t_D}{t_G - t_D} \tag{S1}$$

$$S_{Z_-} = \sigma\{1_{t'_{R,norm(i)}} - 2_{t'_{R,norm(i)}}\} \tag{S2}$$

$$S_{Z_+} = \sigma\{2_{t'_{R,norm(i)}} - (1 - 1_{t'_{R,norm(i)}})\} \tag{S3}$$

$$S_{Z_1} = \sigma\{1_{t'_{R,norm(i)}} - 0.5\} \tag{S4}$$

$$S_{Z_2} = \sigma\{2_{t'_{R,norm(i)}} - 0.5\} \tag{S5}$$

$$Z_- = \left|1 - 2.5\left|S_{Z_-} - 1\right|\right| \tag{S6}$$

$$Z_+ = \left|1 - 2.5\left|S_{Z_+} - 1\right|\right| \tag{S7}$$

$$Z_1 = 1 - \left|2.5 \times S_{Z_1} \times \sqrt{2} - 1\right| \tag{S8}$$

$$Z_2 = 1 - \left|2.5 \times S_{Z_2} \times \sqrt{2} - 1\right| \tag{S9}$$

$$A_O = \sqrt{z \cdot z_+ \cdot z_1 \cdot z_+} \tag{S10}$$

$$n_{grd} = t_G / w_b \tag{S11}$$

$$n_{c,2D} = 1_{n_c} \times 2_{n_c} \tag{S12}$$

$$n'_{c,2D} = \frac{1_{n_c} \times 2_{n_c}}{\sqrt{1 + 3.35\left(\frac{2_{t_c} 1_{n_c}}{1_{t_g}}\right)^2}} \tag{S13}$$

Formula (S1). Asterisk equations for calculating orthogonality and peak capacity of 2D LC system.

Table S1. Intra-day precision of the 1D separation (n = 6).

t_R (min)	1	2	3	4	5	6	RSD (%)
8.44	774.9	761.6	766.2	751.1	755.3	785.3	1.66
10.79	1455.6	1470.5	1465.4	1459.3	1496.1	1460.7	1.00
12.88	1734.8	1728.3	1656.6	1737.5	1659.6	1646	2.59
18.48	1125.9	1125.9	1142.3	1121.9	1104.2	1134.1	1.14
20.04	5125.8	5196.6	5136.7	5174	5180.2	5164	0.52

Table S2. Inter-day precision of the 1D separation (n = 3).

t_R (min)	Day1			Day2			Day3			RSD (%)
	1	2	3	1	2	3	1	2	3	
8.44	755.3	761.6	751.1	773.1	788.6	777.6	741.9	741.8	744	2.24
10.79	1496.1	1465.4	1470.5	1495.1	1472.2	1585.7	1508.8	1516.4	1535.5	2.53
12.88	1656.6	1646	1659.6	1632	1659.2	1643.2	1644.7	1629.4	1623.9	0.80
18.48	1125.9	1134.1	1142.3	1169.8	1170.1	1165	1171.9	1188.6	1171.7	1.79
20.04	5180.2	5164	5174	5165.1	5190.1	5174	5101.1	5106.9	5105.6	0.70

Table S3. Intra-day precision of the 2D separation (n = 6).

Compound	t_R (min)	*m*/*z*	1	2	3	4	5	6	RSD (%)
G-Rh1	15.44	637.4316	2.14E+06	2.06E+06	2.02E+06	2.08E+06	2.01E+06	1.94E+06	3.34
CS-Ⅳa	21.33	793.4374	1.94E+07	2.00E+07	1.89E+07	1.93E+07	1.93E+07	1.98E+07	2.02
CS-Ⅳ	19.44	925.4797	1.53E+07	1.53E+07	1.59E+07	1.61E+07	1.48E+07	1.45E+07	4.01
CS-Ⅴ	17.14	955.4903	1.24E+07	1.18E+07	1.16E+07	1.22E+07	1.22E+07	1.24E+07	2.72
G-Re	9.18	945.5423	1.59E+07	1.72E+07	1.56E+07	1.70E+07	1.66E+07	1.59E+07	4.03

Table S4. Inter-day precision of the 2D separation (n = 3).

Compound	t_R (min)	*m/z*	Day1			Day2			Day3			RSD (%)
			1	2	3	1	2	3	1	2	3	
G-Rh1	15.44	637.4316	2.01E+06	1.94E+06	1.81E+06	2.02E+06	2.01E+06	1.94E+06	1.99E+06	2.15E+06	2.02E+06	4.55
CS-Ⅳa	21.33	793.4374	1.90E+07	1.80E+07	1.75E+07	1.89E+07	1.93E+07	1.93E+07	2.02E+07	1.92E+07	1.93E+07	4.17
CS-Ⅳ	19.44	925.4797	1.61E+07	1.79E+07	1.51E+07	1.53E+07	1.53E+07	1.59E+07	1.63E+07	1.65E+07	1.67E+07	5.42
CS-Ⅴ	17.14	955.4903	1.19E+07	1.22E+07	1.20E+07	1.22E+07	1.22E+07	1.24E+07	1.31E+07	1.37E+07	1.31E+07	4.92
G-Re	9.18	945.5423	1.86E+07	1.90E+07	1.81E+07	1.72E+07	1.70E+07	1.66E+07	1.99E+07	1.97E+07	1.87E+07	6.43

Table S5. Repeatability of the offline 2D LC-MS system (n = 6).

Compound	t_R (min)	*m/z*	1	2	3	4	5	6	RSD (%)
G-Rh1	15.44	637.4316	1.48E+06	1.53E+06	1.42E+06	1.49E+06	1.52E+06	1.50E+06	2.62
CS-Ⅳa	21.33	793.4374	1.85E+07	1.71E+07	1.79E+07	1.88E+07	1.78E+07	1.78E+07	3.33
CS-Ⅳ	19.44	925.4797	1.48E+07	1.62E+07	1.49E+07	1.50E+07	1.60E+07	1.55E+07	3.87
CS-Ⅴ	17.14	955.4903	1.10E+07	1.13E+07	1.10E+07	1.10E+07	1.10E+07	1.10E+07	1.11
G-Re	9.18	945.5423	1.76E+07	1.85E+07	1.79E+07	1.81E+07	1.87E+07	1.86E+07	2.39

Table S6. Information of the 612 saponins reported from the *Panax* genus as of 2023;

No.	Saponins	Formula	Exact Mass	Subtype
1	12β-acetoxy-3β,6α,25-trihydroxy-24,25-dihydro_x0002_dammar-(E)-20(22)-ene	$C_{32}H_{54}O_5$	518.3971	PPT
2	peudoginsenoside Rh2	$C_{36}H_{62}O_8$	622.4445	PPD
3	notoginsenoside SFt2	$C_{36}H_{64}O_{10}$	656.4499	PPD
4	notoginsenoside SFt3	$C_{47}H_{78}O_{16}$	898.5290	PPD
5	notoginsenoside SFt4	$C_{47}H_{78}O_{16}$	898.5290	PPD
6	ginsenoside Ra_{1a}	$C_{52}H_{78}O_{21}$	1038.5036	PPD
7	hexanordammaran	$C_{24}H_{40}O_4$	392.2927	Others
8	27-demethyl-(E, E)-20(22),23-dien-3β,6α,12β-trihydr-oxydammar-25-one	$C_{29}H_{46}O_4$	458.3396	PPT
9	oleanolic acid*	$C_{30}H_{48}O_3$	456.3603	OA
10	panaxadione	$C_{30}H_{48}O_5$	488.3502	Miscellaneous
11	3-keto-20(*S*)-protopanaxtriol	$C_{30}H_{50}O_4$	474.3709	PPT
12	3β,6α,12β-trihydroxy-20S,24R-epoxydammar-25-ene	$C_{30}H_{50}O_4$	474.3709	PPT
13	3β,6α,12β-trihydroxy-20S,24S-epoxydammar-25-ene	$C_{30}H_{50}O_4$	474.3709	PPT
14	dammara-(20E)22,25-diene-3β,6α,12β,24S-tetrol	$C_{30}H_{50}O_4$	474.3709	PPT
15	12β,23(R)-epoxydammara-24-ene-3β,6α,20(S)-triol	$C_{30}H_{50}O_4$	474.3709	PPT
16	20(S)-dammar-3-oxo-23-ene-25-hydroperoxyl-12β,20-diol	$C_{30}H_{50}O_5$	490.3658	PPT
17	pseudoginsenoside R1	$C_{30}H_{50}O_5$	490.3658	Others
18	20(S)-dammar-12-oxo-23-ene-25-hydroperoxyl-3β,6α,20-triol	$C_{30}H_{50}O_6$	506.3607	PPT
19	20(S)-dammar-3-oxo-23-ene-25-hydroperoxyl-6α,12β,20-triol	$C_{30}H_{50}O_6$	506.3607	PPT
20	notoginsenoside R10	$C_{30}H_{50}O_9$	554.3455	PPT
21	Protopanoxadiol*	$C_{30}H_{52}O_3$	460.3916	PPD
22	(20S,22S)-dammar-22,25-epoxy-3β,12β,20-triol	$C_{30}H_{52}O_4$	476.3866	PPD
23	24-hydroxy-panaxdiol	$C_{30}H_{52}O_4$	476.3866	PPD
24	dammar-20S,25S-epoxy-3β,12β,26-triol	$C_{30}H_{52}O_4$	476.3866	PPD
25	ginsengenin S_1	$C_{30}H_{52}O_4$	476.3866	PPT
26	ginsengenin S_2	$C_{30}H_{52}O_4$	476.3866	PPT
27	Protopanaxatriol*	$C_{30}H_{52}O_4$	476.3866	PPT
28	24,26-dihydroxy-panaxdiol	$C_{30}H_{52}O_5$	492.3815	PPD
29	26-hydroxyl-24(E)-20(S)-protopanaxtriol	$C_{30}H_{52}O_5$	492.3815	PPT
30	ginsenotransmetin C	$C_{30}H_{52}O_5$	492.3815	PPT
31	20(S)-dammar-25-ene-24(S)-hydroperoxyl-3β,6α,12β,20-tetrol	$C_{30}H_{52}O_6$	508.3764	PPT
32	25-OH-protopanaxadiol	$C_{30}H_{54}O_4$	478.4022	PPD
33	25-OH-protopanaxtriol	$C_{30}H_{54}O_5$	494.3971	PPT
34	20(R)-20-methoxyl-dammarane-3β,12β,25-triol	$C_{31}H_{56}O_4$	492.4179	PPD
35	20(R)-25-methoxyl-dammarane-3β,12β,20-triol	$C_{31}H_{56}O_4$	492.4179	PPD
36	12β-acetoxy-3β,6α,25-trihydroxy-24,25-dihydro-dammar-(E)-20(22)-ene	$C_{32}H_{54}O_5$	518.3971	PPT
37	notoginsenoside ST8	$C_{32}H_{52}O_9$	580.3611	PPT
38	notoginsenoside ST9	$C_{32}H_{52}O_9$	580.3611	PPT
39	3β-acetoxy-6α,12β,25-trihydroxy-24,25-dihydrodammar-(E)-20(22)-ene	$C_{32}H_{54}O_5$	518.3971	PPT
40	6α-acetoxy-3β,12β,20R-trihydroxydammar-24-ene	$C_{32}H_{54}O_5$	518.3971	PPT

41	6α-acetoxy-3β,12β,20R-trihydroxydammar-25-ene	$C_{32}H_{54}O_5$	518.3971	PPT
42	pn-1	$C_{32}H_{54}O_8$	566.3819	PPD
43	ginsenotransmetin B	$C_{32}H_{56}O_4$	504.4179	PPT
44	notoginsenoside ST6	$C_{35}H_{56}O_9$	620.3924	PPT
45	notoginsenoside ST7	$C_{35}H_{56}O_9$	620.3924	PPT
46	saringosteryl glucoside	$C_{35}H_{58}O_7$	590.4183	Miscellaneous
47	floralginsenoside T_b	$C_{35}H_{62}O_{11}$	658.4292	PPT
48	24(R) pseudoginsenoside Rt4	$C_{36}H_{62}O_{10}$	654.4343	OT
49	24(S) pseudoginsenoside Rt4*	$C_{36}H_{62}O_{10}$	654.4343	OT
50	yesanchinoside R1*	$C_{36}H_{62}O_{10}$	654.4343	PPT
51	28-desglucosylchikusetsusaponin Ⅳa*	$C_{36}H_{56}O_9$	632.3924	OA
52	notoginsenoside SP18	$C_{36}H_{58}O_{10}$	650.4030	PPT
53	oleanolic acid 28-O-β-D-glucopyranose*	$C_{36}H_{58}O_8$	618.4132	OA
54	prosaikogenin H*	$C_{36}H_{58}O_8$	618.4132	Others
55	maslinic acid 28-O-β-d-glucopyranoside*	$C_{36}H_{58}O_9$	634.4081	OA
56	floralginsenoside T_a	$C_{36}H_{60}O_{10}$	652.4186	PPT
57	ginsenoside Rh_{11}	$C_{36}H_{60}O_{10}$	652.4186	C17 side-chain varied
58	ginsenoside ST1	$C_{36}H_{60}O_{10}$	652.4186	PPT
59	notoginsenoside Ab2	$C_{36}H_{60}O_{10}$	652.4186	C17 side-chain varied
60	notoginsenoside SP12	$C_{36}H_{60}O_{10}$	652.4186	PPT
61	notoginsenoside SP13	$C_{36}H_{60}O_{10}$	652.4186	PPT
62	notoginsenoside SP15	$C_{36}H_{60}O_{10}$	652.4186	PPT
63	notoginsenoside T1	$C_{36}H_{60}O_{10}$	652.4186	PPT
64	pseudoginsenoside RT6	$C_{36}H_{60}O_{10}$	652.4186	Others
65	ginsenoside Rh_3	$C_{36}H_{60}O_7$	604.4339	PPD
66	ginsenoside Rk_2*	$C_{36}H_{60}O_7$	604.4339	C17 side-chain varied
67	ginsenoside Rh_{16}	$C_{36}H_{60}O_8$	620.4288	PPT
68	ginsenoside Rh_4*	$C_{36}H_{60}O_8$	620.4288	PPT
69	ginsenoside Rk_3*	$C_{36}H_{60}O_8$	620.4288	C17 side-chain varied
70	ginsenoslaloside I	$C_{36}H_{60}O_8$	620.4288	C17 side-chain varied
71	notoginsenoside SFt6	$C_{36}H_{60}O_8$	620.4288	C17 side-chain varied
72	notoginsenoside SFt8	$C_{36}H_{60}O_8$	620.4288	C17 side-chain varied
73	3β,20(S)-dihydroxydammar-24-en-12β,23β-epoxy-20-O-β-D-glucopyranoside	$C_{36}H_{60}O_8$	620.4288	PPD
74	20(S) sanchirhinoside A8	$C_{36}H_{60}O_9$	636.4237	PPT
75	3-oxo-20(*R*)-ginsenoside Rh1	$C_{36}H_{60}O_9$	636.4237	PPT
76	ginsenoside LS_1	$C_{36}H_{60}O_9$	636.4237	PPT
77	ginsenoside Rh_{22}	$C_{36}H_{60}O_9$	636.4237	Others
78	ginsenoside Rh_5	$C_{36}H_{60}O_9$	636.4237	PPT
79	ginsenoside Rh_7	$C_{36}H_{60}O_9$	636.4237	Miscellaneous
80	ginsenoside Rh_8	$C_{36}H_{60}O_9$	636.4237	Miscellaneous
81	ginsenoside Rh_9	$C_{36}H_{60}O_9$	636.4237	PPD
82	ginsenoside Rk_6	$C_{36}H_{60}O_9$	636.4237	C17 side-chain varied
83	notoginsenoside Ab1	$C_{36}H_{60}O_9$	636.4237	C17 side-chain varied

84	notoginsenoside Ab3	$C_{36}H_{60}O_9$	636.4237	C17 side-chain varied
85	notoginsenoside ST14	$C_{36}H_{60}O_9$	636.4237	PPT
86	chikusetsusaponin L_{9a}*	$C_{36}H_{62}O_{10}$	654.4343	PPT
87	ginsenoside Ki	$C_{36}H_{62}O_{10}$	654.4343	PPT
88	ginsenoside Km	$C_{36}H_{62}O_{10}$	654.4343	PPT
89	ginsenoside M7cd	$C_{36}H_{62}O_{10}$	654.4343	PPT
90	ginsenoside SG_1	$C_{36}H_{62}O_{10}$	654.4343	Others
91	ginsenoside ST_2	$C_{36}H_{62}O_{10}$	654.4343	PPT
92	notoginsenoside R8	$C_{36}H_{62}O_{10}$	654.4343	PPT
93	notoginsenoside R9	$C_{36}H_{62}O_{10}$	654.4343	PPT
94	notoginsenoside SP14	$C_{36}H_{62}O_{10}$	654.4343	PPT
95	notoginsenoside SP21	$C_{36}H_{62}O_{10}$	654.4343	PPT
96	notoginsenoside ST1	$C_{36}H_{62}O_{10}$	654.4343	PPT
97	notoginsenoside ST6	$C_{36}H_{62}O_{10}$	654.4343	PPT
98	notoginsenoside ST7	$C_{36}H_{62}O_{10}$	654.4343	PPT
99	notopanaxoside A	$C_{36}H_{62}O_{10}$	654.4343	PPT
100	pseudoginsenoside RT4*	$C_{36}H_{62}O_{10}$	654.4343	OT
101	pseudoginsenoside RT5	$C_{36}H_{62}O_{10}$	654.4343	OT
102	vinaginsenoside R10	$C_{36}H_{62}O_{10}$	654.4343	PPT
103	yesanchinoside R3*	$C_{36}H_{62}O_{10}$	654.4343	PPT
104	20(*R*) ginsenoside ST2	$C_{36}H_{62}O_{10}$	654.4343	PPT
105	20(*R*) ginsenoside-SF	$C_{36}H_{62}O_{10}$	654.4343	PPT
106	floralginsenoside Ka	$C_{36}H_{62}O_{11}$	670.4292	PPT
107	floralquinquenoside A	$C_{36}H_{62}O_{11}$	670.4292	PPT
108	ginsenoside Rh_6	$C_{36}H_{62}O_{11}$	670.4292	PPD
109	ginsenoside SF	$C_{36}H_{62}O_{11}$	670.4292	PPT
110	ginsenoside SG_2	$C_{36}H_{62}O_{11}$	670.4292	Others
111	ginsenoside SL_1	$C_{36}H_{62}O_{11}$	670.4292	PPT
112	notoginsenoside SP4	$C_{36}H_{62}O_{11}$	670.4292	PPT
113	notoginsenoside SP5	$C_{36}H_{62}O_{11}$	670.4292	PPT
114	notoginsenoside SP6	$C_{36}H_{62}O_{11}$	670.4292	PPT
115	notoginsenoside SP7	$C_{36}H_{62}O_{11}$	670.4292	PPT
116	notoginsenoside SP8	$C_{36}H_{62}O_{11}$	670.4292	PPT
117	notoginsenoside T4	$C_{36}H_{62}O_{11}$	670.4292	PPT
118	20(*S*) ginsenoside SG2	$C_{36}H_{62}O_{11}$	670.4292	others
119	compound K	$C_{36}H_{62}O_8$	622.4445	PPD
120	ginsenoside Rh_2*	$C_{36}H_{62}O_8$	622.4445	PPD
121	ginsenosides Rh_{10}	$C_{36}H_{62}O_8$	622.4445	PPD
122	notoginsenoside R7	$C_{36}H_{62}O_8$	622.4445	PPD
123	notoginsenoside SFt7	$C_{36}H_{62}O_8$	622.4445	C17 side-chain varied
124	ginsenoside M_1	$C_{36}H_{62}O_8$	622.4445	PPD
125	20(R) ginsenoside Rh1*	$C_{36}H_{62}O_9$	638.4394	PPT
126	chikusetsusaponin L_{10}*	$C_{36}H_{62}O_9$	638.4394	PPT
127	ginsenoside F_1*	$C_{36}H_{62}O_9$	638.4394	PPT
128	ginsenoside Rh_{13}	$C_{36}H_{62}O_9$	638.4394	PPD
129	ginsenoside Rh_{19}	$C_{36}H_{62}O_9$	638.4394	PPT
130	notoginsenoside SFt1	$C_{36}H_{62}O_9$	638.4394	PPD
131	notoginsenoside SFt5	$C_{36}H_{62}O_9$	638.4394	C17 side-chain varied
132	notoginsenoside ST13	$C_{36}H_{62}O_9$	638.4394	PPT
133	sanchinoside B1	$C_{36}H_{62}O_9$	638.4394	PPT
134	20(R) ginsenoside Rh19	$C_{36}H_{62}O_9$	638.4394	PPT

135	20(S) ginsenoside Rh1*	$C_{36}H_{62}O_9$	638.4394	PPT
136	20(S) ginsenoside Rh19	$C_{36}H_{62}O_9$	638.4394	PPT
137	25-hydroxyginsenoside Rk3	$C_{36}H_{62}O_9$	638.4394	C17 side-chain varied
138	3-O-β-D-glucopyranosyl-20(S)-protopanaxtriol	$C_{36}H_{62}O_9$	638.4394	PPT
139	ginsenoside Rh12	$C_{36}H_{64}O_{10}$	656.4499	PPD
140	20(*R*)-25-hydroxyginsenoside Rh1	$C_{36}H_{64}O_{10}$	656.4499	PPT
141	vinaginsenoside R12	$C_{36}H_{64}O_{11}$	672.4449	PPT
142	28-desglucosylchikusetsusaponin Ⅳa methyl ester*bai	$C_{37}H_{58}O_9$	646.4081	OA
143	3-formyloxy-20-O-β-D-glucopyranosyl-20(S)-protopanaxtriol	$C_{37}H_{62}O_{10}$	666.4343	PPT
144	ginsenoside Rh23	$C_{37}H_{64}O_{10}$	666.4343	PPT
145	notoginsenoside SP10	$C_{37}H_{64}O_{11}$	682.4292	PPT
146	notoginsenoside SP20	$C_{37}H_{64}O_{11}$	682.4292	PPT
147	notoginsenoside SP9	$C_{37}H_{64}O_{11}$	682.4292	PPT
148	ginsenoside Rs6	$C_{38}H_{60}O_8$	644.4288	C17 side-chain varied
149	28-desglucosylchikusetsusaponin Ⅳa ethyl ester	$C_{38}H_{60}O_9$	660.4237	OA
150	notoginsenoside ST10	$C_{38}H_{62}O_{13}$	726.419	PPD
151	ginsenoside Rs7	$C_{38}H_{62}O_9$	662.4394	PPT
152	20(R) ginsenoside Rh1 6′-acetate*	$C_{38}H_{64}O_{10}$	680.4499	PPT
153	6'-acetyl ginsenoside-F1	$C_{38}H_{64}O_{10}$	680.4499	PPT
154	20(S) ginsenoside-Rh1-6′-acetate	$C_{38}H_{64}O_{10}$	680.4499	PPT
155	3β-acetoxyl ginsenoside F1*	$C_{38}H_{64}O_{10}$	680.4499	PPT
156	notoginsenoside T3	$C_{38}H_{66}O_9$	666.4707	PPT
157	malonylginsenoside Rk2	$C_{39}H_{62}O_{10}$	678.4343	C17 side-chain varied
158	malonylginsenoside Rk3	$C_{39}H_{62}O_{11}$	694.4292	C17 side-chain varied
159	28-desglucosylchikusetsusaponin Ⅳa butyl ester*	$C_{40}H_{64}O_9$	688.4550	OA
160	20(S) sanchirhinoside A9	$C_{40}H_{66}O_{10}$	706.4656	PPT
161	20(*S*) sanchirhinoside A1	$C_{40}H_{66}O_{10}$	706.4656	PPT
162	dammar-12,24-dien-3α,6β,15α-triol-3α-D-arabinopyra-nosyl-6β-L-arabinopyranoside	$C_{40}H_{66}O_{11}$	722.4605	Miscellaneous
163	6′-malonyl methyl ester ginsenoside F1	$C_{40}H_{66}O_{12}$	738.4554	PPT
164	dammar-24-en-3α,6β,16α,20β-tetraol-3α-D-arabinopy-ranosyl-6β-D-arabinopyranoside	$C_{40}H_{66}O_{12}$	738.4554	Miscellaneous
165	20(*R*) protopanaxadiol	$C_{40}H_{70}O_{12}$	742.4867	PPD
166	chikusetsusaponin Ia*	$C_{41}H_{70}O_{12}$	754.4867	PPD
167	24(S) majoroside R2*	$C_{41}H_{70}O_{14}$	786.4766	OT
168	yesanchinoside R2*	$C_{41}H_{70}O_{14}$	786.4766	PPT
169	28-desglucosylchikusetsusaponin Ⅳ*	$C_{41}H_{64}O_{13}$	764.4347	OA
170	pseudoginsenoside Rp1*	$C_{41}H_{64}O_{13}$	764.4347	OA
171	pjs-4*	$C_{41}H_{66}O_{12}$	750.4554	OA
172	notoginsenoside ST11	$C_{41}H_{68}O_{11}$	736.4762	PPD
173	notoginsenoside ST12	$C_{41}H_{68}O_{11}$	736.4762	PPD
174	notoginsenoside-LY	$C_{41}H_{68}O_{12}$	752.4711	PPD
175	notoginsenoside T5*	$C_{41}H_{68}O_{12}$	752.4711	PPT
176	chikusetsusaponin FH1*	$C_{41}H_{68}O_{13}$	768.4660	Others
177	chikusetsusaponin FT1*	$C_{41}H_{68}O_{13}$	768.4660	Others
178	notoginsenoside SY3	$C_{41}H_{68}O_{14}$	784.4609	PPD
179	notoginsenoside SY1	$C_{41}H_{68}O_{17}$	832.4457	PPD

180	ginsenoside Mc	$C_{41}H_{70}O_{12}$	754.4867	PPD
181	20(R) notoginsenoside R2	$C_{41}H_{70}O_{13}$	770.4816	PPT
182	20(R) sanchirhinoside A3*	$C_{41}H_{70}O_{13}$	770.4816	PPT
183	20(R) sanchirhinoside A4*	$C_{41}H_{70}O_{13}$	770.4816	PPT
184	20(S) notoginsenoside R2	$C_{41}H_{70}O_{13}$	770.4816	PPT
185	20(S) sanchirhinoside A3	$C_{41}H_{70}O_{13}$	770.4816	PPT
186	6-O-(β-D-glucopyranosyl)-20-O-(β-D-xylopyranosyl)-3β,6α,12β,20(S)-tetrahydroxydammar-ene	$C_{41}H_{70}O_{13}$	770.4816	PPT
187	chikusetsusaponin L_8	$C_{41}H_{70}O_{13}$	770.4816	PPT
188	chikusetsusaponin LM_1*	$C_{41}H_{70}O_{13}$	770.4816	PPT
189	ginsenoside F_3*	$C_{41}H_{70}O_{13}$	770.4816	PPT
190	ginsenoside F_5*	$C_{41}H_{70}O_{13}$	770.4816	PPT
191	notoginsenoside R2*	$C_{41}H_{70}O_{13}$	770.4816	PPT
192	peudoginsenoside RT3*	$C_{41}H_{70}O_{13}$	770.4816	PPT
193	pseudoginsenoside RT2*	$C_{41}H_{70}O_{13}$	770.4816	OT
194	24(R) majoroside R2*	$C_{41}H_{70}O_{14}$	786.4766	OT
195	majonoside R2*	$C_{41}H_{70}O_{14}$	786.4766	OT
196	notoginsenoside Rw_2	$C_{41}H_{70}O_{14}$	786.4766	PPT
197	vinaginsenoside R11	$C_{41}H_{70}O_{14}$	786.4766	PPT
198	floralginsenoside C	$C_{41}H_{70}O_{15}$	802.4715	PPT
199	floralginsenoside D	$C_{41}H_{70}O_{15}$	802.4715	PPT
200	vinaginsenoside R14	$C_{41}H_{70}O_{15}$	802.4715	PPT
201	chikusetsusaponin II*	$C_{42}H_{66}O_{14}$	794.4453	OA
202	24(S) Pseudoginsenoside F11*	$C_{42}H_{72}O_{14}$	800.4922	OT
203	vinaginsenoside R15*	$C_{42}H_{72}O_{15}$	816.4871	C17 side-chain varied
204	notoginsenoside J*	$C_{42}H_{74}O_{16}$	834.4977	PPT
205	28-desglucosylchikusetsusaponin Ⅳ methyl ester	$C_{42}H_{66}O_{13}$	778.4503	OA
206	bipinoside A*	$C_{42}H_{66}O_{13}$	778.4503	OA
207	chikusetsusaponin IVa*	$C_{42}H_{66}O_{14}$	794.4453	OA
208	zingibroside R1*	$C_{42}H_{66}O_{14}$	794.4453	OA
209	cynarasaponin C*	$C_{42}H_{66}O_{15}$	810.4402	UA
210	ginsenoside F_4*	$C_{42}H_{70}O_{12}$	766.4867	PPT
211	ginsenoside Rg_4	$C_{42}H_{70}O_{12}$	766.4867	PPT
212	ginsenoside Rg_5*	$C_{42}H_{70}O_{12}$	766.4867	PPD
213	ginsenoside Rg_6*	$C_{42}H_{70}O_{12}$	766.4867	PPD
214	ginsenoside Rk_1*	$C_{42}H_{70}O_{12}$	766.4867	C17 side-chain varied
215	ginsenoside Rz_1	$C_{42}H_{70}O_{12}$	766.4867	PPD
216	sanchirhinoside D	$C_{42}H_{70}O_{12}$	766.4867	C17 side-chain varied
217	20(E) ginsenoside Rg9*	$C_{42}H_{70}O_{13}$	782.4816	PPT
218	12-O-glucoginsenoside Rh4	$C_{42}H_{70}O_{13}$	782.4816	PPT
219	20(Z) ginsenoside Rg9	$C_{42}H_{70}O_{13}$	782.4816	PPT
220	ginsenoside La	$C_{42}H_{70}O_{13}$	782.4816	PPT
221	ginsenoside Rh_{14}	$C_{42}H_{70}O_{13}$	782.4816	PPT
222	ginsenoside Rh_{15}	$C_{42}H_{70}O_{13}$	782.4816	PPT
223	ginsenoside Rh_{17}	$C_{42}H_{70}O_{13}$	782.4816	Miscellaneous
224	sanchirhinoside B	$C_{42}H_{70}O_{13}$	782.4816	PPT
225	12,23-epoxyginsenoside-Rg1	$C_{42}H_{70}O_{14}$	798.4766	PPT
226	12-one-pseudoginsenoside F11	$C_{42}H_{70}O_{14}$	798.4766	Miscellaneous
227	ginsenoside Rg_{10}	$C_{42}H_{70}O_{14}$	798.4766	PPT

228	ginsenoside Rg_8	$C_{42}H_{70}O_{14}$	798.4766	PPT
229	ginsenoside SL_2	$C_{42}H_{70}O_{14}$	798.4766	PPT
230	ginsenoside SL_3	$C_{42}H_{70}O_{14}$	798.4766	PPT
231	pseudoginsenoside G1	$C_{42}H_{70}O_{14}$	798.4766	Others
232	pseudoginsenoside G2	$C_{42}H_{70}O_{14}$	798.4766	Others
233	ginsenoside Rg_{11}	$C_{42}H_{70}O_{14}$	798.4766	PPD
234	pseudoginsenoside RT8	$C_{42}H_{70}O_{15}$	814.4715	Others
235	vinaginsenoside R25	$C_{42}H_{70}O_{15}$	814.4715	PPT
236	20(*S*) sanchirhinoside A7	$C_{42}H_{72}O_{13}$	784.4973	PPT
237	25-hydroxyl-(E)-20(22)-ene-ginsenoside Rg3	$C_{42}H_{72}O_{13}$	784.4973	PPD
238	ginsenoside F_2*	$C_{42}H_{72}O_{13}$	784.4973	PPD
239	ginsenoside Rg_2*	$C_{42}H_{72}O_{13}$	784.4973	PPT
240	25-hydroxylginsenoside Rk1	$C_{42}H_{72}O_{13}$	784.4973	C17 side-chain varied
241	ginsenoside Rg_3*	$C_{42}H_{72}O_{13}$	784.4973	PPD
242	20(S) ginsenoside-Rf-1a	$C_{42}H_{72}O_{14}$	800.4922	PPT
243	20(S) sanchirhinoside A4	$C_{42}H_{72}O_{14}$	800.4922	PPT
244	24(R) pseudoginsenoside F11*	$C_{42}H_{72}O_{14}$	800.4922	OT
245	ginsenoside Ia	$C_{42}H_{72}O_{14}$	800.4922	PPT
246	ginsenoside Ib	$C_{42}H_{72}O_{14}$	800.4922	Others
247	ginsenoside Rf*	$C_{42}H_{72}O_{14}$	800.4922	PPT
248	ginsenoside Rg_1*	$C_{42}H_{72}O_{14}$	800.4922	PPT
249	ginsenoside Rg_7	$C_{42}H_{72}O_{14}$	800.4922	PPD
250	ginsenosides Rh_{20}	$C_{42}H_{72}O_{14}$	800.4922	PPT
251	majoroside F2*	$C_{42}H_{72}O_{14}$	800.4922	C17 side-chain varied
252	majoroside F3*	$C_{42}H_{72}O_{14}$	800.4922	C17 side-chain varied
253	majoroside F4*	$C_{42}H_{72}O_{14}$	800.4922	PPD
254	notoginsenoside ST-8	$C_{42}H_{72}O_{14}$	800.4922	PPT
255	notoginsenoside U	$C_{42}H_{72}O_{14}$	800.4922	PPT
256	20(S)-6-O-[β-D-glucopyranosyl-(1→2)-β-D-glucopyranosyl]-dammar-20,25-epoxy-3β,6*α*,12β,24*α*-tetriol	$C_{42}H_{72}O_{15}$	816.4871	PPT
257	24(R) majoroside R1*	$C_{42}H_{72}O_{15}$	816.4871	OT
258	6-O-[β-D-glucopyranosyl-(1→2)-β-D-glucopyranosyl]-dammar-25(26)-ene-3β,6*α*,12β,20S,24R-pentaol	$C_{42}H_{72}O_{15}$	816.4871	PPT
259	floralginsenoside E*	$C_{42}H_{72}O_{15}$	816.4871	PPD
260	floralginsenoside F	$C_{42}H_{72}O_{15}$	816.4871	PPD
261	floralquinquenoside B*	$C_{42}H_{72}O_{15}$	816.4871	PPT
262	floralquinquenoside C	$C_{42}H_{72}O_{15}$	816.4871	PPT
263	floralquinquenoside D*	$C_{42}H_{72}O_{15}$	816.4871	PPD
264	ginsenoside Re_5	$C_{42}H_{72}O_{15}$	816.4871	PPT
265	ginsenoside Rg_{12}	$C_{42}H_{72}O_{15}$	816.4871	PPD
266	ginsenoside Rh_{21}	$C_{42}H_{72}O_{15}$	816.4871	PPT
267	majonoside R1	$C_{42}H_{72}O_{15}$	816.4871	OT
268	notoginsenoside SP1	$C_{42}H_{72}O_{15}$	816.4871	PPD
269	notoginsenoside SP11	$C_{42}H_{72}O_{15}$	816.4871	PPD
270	notoginsenoside SP2	$C_{42}H_{72}O_{15}$	816.4871	PPD
271	notoginsenoside SP3	$C_{42}H_{72}O_{15}$	816.4871	PPD
272	panajaponol A	$C_{42}H_{72}O_{15}$	816.4871	PPT

273	(20S,24S,25R)-6-O-[β-D-glucopyranosyl-(1→2)-β-D-glucopyranosyl]-dammar-20,24-epoxy-3β,6α,12β,25,26-pentaol	$C_{42}H_{72}O_{16}$	832.4820	OT
274	floralginsenoside A	$C_{42}H_{72}O_{16}$	832.4820	PPT
275	floralginsenoside B*	$C_{42}H_{72}O_{16}$	832.4820	PPT
276	ursan-3β,19α,22β-triol-3-O-β-D-glucopyranosyl-(2′→1″)-β-D-glucopyranoside	$C_{42}H_{73}O_{13}$	785.5051	Miscellaneous
277	20(S) ginsenoside Rf2	$C_{42}H_{74}O_{14}$	802.5079	PPT
278	quinquenoside L9	$C_{42}H_{74}O_{15}$	818.5028	PPT
279	24(S) vinaginsenoside R21	$C_{42}H_{74}O_{16}$	834.4977	PPT
280	24(R) vinaginsenoside R22	$C_{42}H_{74}O_{16}$	834.4977	PPT
281	6-O-[β-D-glucopyranosyl-(1→2)-β-D-glucopyranosyl]-dammar-3β,6α,12β,20S,24R,25-hexaol	$C_{42}H_{74}O_{16}$	834.4977	PPT
282	28-desglucosylchikusetsusaponin Ⅳethyl ester	$C_{43}H_{68}O_{13}$	792.4660	OA
283	chikusetsusaponin IVa methyl ester*	$C_{43}H_{68}O_{14}$	808.4609	OA
284	spinasaponin A methyl ester*	$C_{43}H_{68}O_{14}$	808.4609	OA
285	zingibroside R1 methyl ester*	$C_{43}H_{68}O_{14}$	808.4609	OA
286	23-*O*-methylginsenoside-Rg11	$C_{43}H_{72}O_{14}$	812.4922	PPT
287	notoginsenoside SP17	$C_{43}H_{72}O_{14}$	812.4922	PPD
288	sanchirhinoside A2	$C_{43}H_{72}O_{14}$	812.4922	PPT
289	24(R) vinaginsenoside R2	$C_{43}H_{72}O_{15}$	828.4871	OT
290	24(S) vinaginsenoside R2*	$C_{43}H_{72}O_{15}$	828.4871	OT
291	20(R) methoxyl-ginsenoside Rg3	$C_{43}H_{74}O_{13}$	789.5129	PPD
292	20(S) methoxyl-ginsenoside Rg3	$C_{43}H_{74}O_{13}$	789.5129	PPD
293	notoginsenoside SY4	$C_{43}H_{74}O_{14}$	815.5079	PPD
294	notoginsenoside ST-2	$C_{43}H_{74}O_{15}$	830.5028	PPD
295	notoginsenoside ST-3	$C_{43}H_{74}O_{15}$	830.5028	PPD
296	dammar-9(11),24-dien-3β-ol-3α-L-arabinosyl-7α-octanoate	$C_{43}H_{75}O_{7}$	703.5513	Others
297	24(R) yesanchinoside A*	$C_{44}H_{74}O_{16}$	858.4977	OT
298	24(S) yesanchinoside A*	$C_{44}H_{74}O_{16}$	858.4977	OT
299	chikusetsusaponin Ⅳa ethyl ester*	$C_{44}H_{70}O_{14}$	822.4766	OA
300	zingibroside R1 ethyl ester	$C_{44}H_{70}O_{14}$	822.4766	OA
301	ginsenoside Rs_4	$C_{44}H_{72}O_{13}$	808.4973	PPD
302	ginsenoside Rs_5*	$C_{44}H_{72}O_{13}$	808.4973	PPD
303	notoginsenosides NL-D	$C_{44}H_{72}O_{16}$	856.482	PPT
304	notoginsenoside NL-A_4	$C_{44}H_{72}O_{17}$	872.477	PPD
305	acetylated Rg2	$C_{44}H_{74}O_{14}$	826.5079	PPT
306	ginsenoside Rs_3*	$C_{44}H_{74}O_{14}$	826.5079	PPD
307	24(R) vinaginsenoside R1	$C_{44}H_{74}O_{15}$	842.5028	OT
308	24(S) vinaginsenoside R1*	$C_{44}H_{74}O_{15}$	842.5028	OT
309	acetylginsenoside Rf*	$C_{44}H_{74}O_{15}$	842.5028	PPT
310	acetylginsenoside Rg1*	$C_{44}H_{74}O_{15}$	842.5028	PPT
311	vinaginsenoside R1*	$C_{44}H_{74}O_{15}$	842.5028	OT
312	yesanchinoside D*	$C_{44}H_{74}O_{15}$	842.5028	PPT
313	28-desglucosylchikusetsusaponin Ⅳ butyl ester	$C_{45}H_{72}O_{13}$	820.4973	OA
314	malonylginsenoside Rg5	$C_{45}H_{72}O_{15}$	852.4871	PPD
315	malonylginsenoside Rg6	$C_{45}H_{72}O_{15}$	852.4871	PPD
316	malonylginsenoside Rk1	$C_{45}H_{72}O_{15}$	852.4871	C17 side-chain varied
317	malonylginsenoside Rg2	$C_{45}H_{74}O_{16}$	870.4977	PPT
318	malonylginsenoside Rg3	$C_{45}H_{74}O_{16}$	870.4977	PPD
319	malonylginsenoside Rf	$C_{45}H_{74}O_{17}$	886.4926	PPT

320	malonylginsenoside Rg1*	$C_{45}H_{74}O_{17}$	886.4926	PPT
321	floralginsenoside Kb	$C_{45}H_{76}O_{19}$	920.4981	PPD
322	floralginsenoside Kc	$C_{45}H_{76}O_{20}$	936.4930	PPD
323	chikusetsusaponin IVa butyl ester*	$C_{46}H_{74}O_{14}$	850.5079	OA
324	zingibroside R1 butyl ester	$C_{46}H_{74}O_{14}$	850.5079	OA
325	ginsenoside Re_6	$C_{46}H_{76}O_{15}$	868.5184	PPT
326	koryoginenoside R1	$C_{46}H_{76}O_{15}$	868.5184	PPT
327	chikusetsusaponin L_5*	$C_{46}H_{78}O_{17}$	902.5239	PPT
328	chikusetsusaponin LM_2*	$C_{46}H_{78}O_{17}$	902.5239	PPT
329	chikusetsusaponin LM_3*	$C_{46}H_{78}O_{17}$	902.5239	PPT
330	notoginsenoside Rw_1	$C_{46}H_{78}O_{17}$	902.5239	PPT
331	vinaginsenoside R2*	$C_{47}H_{70}O_{13}$	842.4816	OT
332	chikusetsusaponin Ib*	$C_{47}H_{74}O_{18}$	926.4875	OA
333	Chikusetsusaponin IV*	$C_{47}H_{74}O_{18}$	926.4875	OA
334	pjs-2*	$C_{47}H_{74}O_{18}$	926.4875	OA
335	pseudoginsenoside Rt1*	$C_{47}H_{74}O_{18}$	926.4875	OA
336	stipuleanoside R1*	$C_{47}H_{74}O_{18}$	926.4875	OA
337	notoginsenoside SFt10	$C_{47}H_{78}O_{16}$	898.5290	C17 side-chain varied
338	chikusetsusaponin FT_4*	$C_{47}H_{78}O_{17}$	914.5239	Others
339	notoginsenoside LX	$C_{47}H_{78}O_{17}$	914.5239	PPD
340	notoginsenoside Ng_1	$C_{47}H_{78}O_{17}$	914.5239	PPD
341	notoginsenoside SY2	$C_{47}H_{78}O_{17}$	914.5239	PPD
342	notoginsenoside NL-C_1	$C_{47}H_{78}O_{18}$	930.5188	PPD
343	notoginsenoside NL-C_2	$C_{47}H_{78}O_{18}$	930.5188	PPD
344	24(R) yesanchinoside C*	$C_{47}H_{80}O_{19}$	948.5294	OT
345	24(S) yesanchinoside C*	$C_{47}H_{80}O_{19}$	948.5294	OT
346	chikusetsusaponin III*	$C_{47}H_{80}O_{17}$	916.5396	PPD
347	ginsenoside Mb	$C_{47}H_{80}O_{17}$	916.5396	PPD
348	ginsenoside Rd_2	$C_{47}H_{80}O_{17}$	916.5396	PPD
349	ginsenoside Rh_{24}	$C_{47}H_{80}O_{17}$	916.5396	PPT
350	gypenoside IX	$C_{47}H_{80}O_{17}$	916.5396	PPD
351	notoginsenoside Fe*	$C_{47}H_{80}O_{17}$	916.5396	PPD
352	notoginsenoside Ft_1	$C_{47}H_{80}O_{17}$	916.5396	PPD
353	quinquenoside L10	$C_{47}H_{80}O_{17}$	916.5396	PPD
354	quinquenoside L14	$C_{47}H_{80}O_{17}$	916.5396	PPD
355	vinaginsenoside R16*	$C_{47}H_{80}O_{17}$	916.5396	PPD
356	vinaginsenoside R17*	$C_{47}H_{80}O_{17}$	916.5396	PPD
357	vinaginsenoside R18	$C_{47}H_{80}O_{17}$	916.5396	PPD
358	notoginsenoside NL-H_1	$C_{47}H_{80}O_{18}$	932.5345	PPD
359	20(*S*) sanchirhinoside A5	$C_{47}H_{80}O_{18}$	932.5345	PPT
360	ginsenoside Re_4*	$C_{47}H_{80}O_{18}$	932.5345	PPT
361	notoginsenoside Fp_1*	$C_{47}H_{80}O_{18}$	932.5345	PPT
362	notoginsenoside Ft_3	$C_{47}H_{80}O_{18}$	932.5345	PPD
363	notoginsenoside LK_8	$C_{47}H_{80}O_{18}$	932.5345	PPD
364	notoginsenoside NL-B_1	$C_{47}H_{80}O_{18}$	932.5345	PPD
365	notoginsenoside NL-G_1	$C_{47}H_{80}O_{18}$	932.5345	PPD
366	notoginsenoside NL-H_2	$C_{47}H_{80}O_{18}$	932.5345	PPD
367	notoginsenoside R1*	$C_{47}H_{80}O_{18}$	932.5345	PPT
368	notoginsenoside ST-5	$C_{47}H_{80}O_{18}$	932.5345	PPD
369	quinquenoside F6	$C_{47}H_{80}O_{18}$	932.5345	PPT
370	quinquenoside L17*	$C_{47}H_{80}O_{18}$	932.5345	PPT
371	quinquenoside L3	$C_{47}H_{80}O_{18}$	932.5345	PPD
372	notoginsenoside H	$C_{47}H_{80}O_{19}$	948.5294	PPT

373	notoginsenoside NL-A_1	$C_{47}H_{80}O_{19}$	948.5294	PPD
374	notoginsenoside NL-A_2	$C_{47}H_{80}O_{19}$	948.5294	PPD
375	notoginsenoside SFt9	$C_{47}H_{80}O_{19}$	948.5294	C17 side-chain varied
376	vinaginsenoside R5	$C_{47}H_{80}O_{19}$	948.5294	OT
377	vinaginsenoside R6*	$C_{47}H_{80}O_{19}$	948.5294	OT
378	3β,4α,12β-trihydroxystigmast-5-en-21-yloctadecan-9′,12′-dienoate	$C_{47}H_{80}O_5$	724.6006	Others
379	notoginsenoside Ft_2	$C_{47}H_{82}O_{18}$	934.5501	PPD
380	notoginsenoside NL-E_1	$C_{47}H_{82}O_{19}$	950.545	PPD
381	chikusetsusaponin IV methyl ester*	$C_{48}H_{76}O_{18}$	940.5032	OA
382	araloside A methyl ester	$C_{48}H_{76}O_{18}$	940.5032	OA
383	bipinoside B	$C_{48}H_{76}O_{18}$	940.5032	OA
384	pseudoginsenoside Rt1 methyl ester*	$C_{48}H_{76}O_{18}$	940.5032	OA
385	chikusetsusaponin V*	$C_{48}H_{76}O_{19}$	956.4981	OA
386	hemsgiganoside B*	$C_{48}H_{76}O_{19}$	956.4981	OA
387	spinasaponin A 28-*O*-glucoside	$C_{48}H_{76}O_{19}$	956.4981	OA
388	tuberoside A*	$C_{48}H_{76}O_{19}$	956.4981	OA
389	5,6-didehydroginsenoside Rd	$C_{48}H_{80}O_{18}$	944.5345	Miscellaneous
390	chikusetsusaponin FK_2*	$C_{48}H_{80}O_{18}$	944.5345	Miscellaneous
391	ginsenoside Rh_{18}	$C_{48}H_{80}O_{18}$	944.5345	PPT
392	quinquefoloside Ld	$C_{48}H_{80}O_{18}$	944.5345	PPT
393	quinquefoloside Le	$C_{48}H_{80}O_{18}$	944.5345	PPD
394	quinquenoside L1*	$C_{48}H_{80}O_{18}$	944.5345	PPD
395	ginsenoside III*	$C_{48}H_{80}O_{19}$	960.5294	PPD
396	notoginsenoside G*	$C_{48}H_{80}O_{19}$	960.5294	Miscellaneous
397	vinaginsenoside R20	$C_{48}H_{80}O_{19}$	960.5294	PPT
398	vinaginsenoside R3*	$C_{48}H_{82}O_{17}$	930.5552	Miscellaneous
399	chikusetsusaponin FK_1*	$C_{48}H_{82}O_{18}$	946.5501	PPT
400	chikusetsusaponin FK_7*	$C_{48}H_{82}O_{18}$	946.5501	PPD
401	ginsenoside Rd*	$C_{48}H_{82}O_{18}$	946.5501	PPD
402	ginsenoside Re*	$C_{48}H_{82}O_{18}$	946.5501	PPT
403	notoginsenoside K*	$C_{48}H_{82}O_{18}$	946.5501	PPD
404	ginsenoside Rd_6	$C_{48}H_{82}O_{18}$	946.5501	PPD
405	ginsenoside Rg_{18}	$C_{48}H_{82}O_{18}$	946.5501	PPT
406	7β-hydroxyl ginsenoside Rd	$C_{48}H_{82}O_{19}$	962.5450	PPD
407	majoroside F1	$C_{48}H_{82}O_{19}$	962.5450	PPD
408	20-glc-ginsenoside-Rf*	$C_{48}H_{82}O_{19}$	962.5450	PPT
409	chikusetsusaponin LM4*	$C_{48}H_{82}O_{19}$	962.5450	PPT
410	floralginsenoside La	$C_{48}H_{82}O_{19}$	962.5450	PPT
411	floralginsenoside Lb	$C_{48}H_{82}O_{19}$	962.5450	PPT
412	ginsenoside Re_1*	$C_{48}H_{82}O_{19}$	962.5450	PPT
413	ginsenoside Re_2*	$C_{48}H_{82}O_{19}$	962.5450	PPT
414	ginsenoside Re_3*	$C_{48}H_{82}O_{19}$	962.5450	PPT
415	ginsenoside Re_7	$C_{48}H_{82}O_{19}$	962.5450	PPT
416	majoroside F5	$C_{48}H_{82}O_{19}$	962.5450	PPT
417	majoroside F6	$C_{48}H_{82}O_{19}$	962.5450	PPT
418	notoginsenoside M*	$C_{48}H_{82}O_{19}$	962.5450	PPT
419	notoginsenoside N*	$C_{48}H_{82}O_{19}$	962.5450	PPT
420	notoginsenoside R3*	$C_{48}H_{82}O_{19}$	962.5450	PPT
421	notoginsenoside R6*	$C_{48}H_{82}O_{19}$	962.5450	PPT
422	quinquenoside L2	$C_{48}H_{82}O_{19}$	962.5450	PPD
423	vinaginsenoside R24	$C_{48}H_{82}O_{19}$	962.5450	PPD
424	vinaginsenoside R4	$C_{48}H_{82}O_{19}$	962.5450	PPT

425	vinaginsenoside R8	$C_{48}H_{82}O_{19}$	962.5450	PPD
426	vinaginsenoside R9	$C_{48}H_{82}O_{19}$	962.5450	PPD
427	bipinnatifidusoside F2	$C_{48}H_{82}O_{20}$	978.5399	PPT
428	floralginsenoside I	$C_{48}H_{82}O_{20}$	978.5399	PPT
429	floralginsenoside J	$C_{48}H_{82}O_{20}$	978.5399	PPT
430	floralginsenoside K	$C_{48}H_{82}O_{20}$	978.5399	PPT
431	ginsenoside I	$C_{48}H_{82}O_{20}$	978.5399	PPD
432	ginsenoside II	$C_{48}H_{82}O_{20}$	978.5399	PPD
433	ginsenoside S_4	$C_{48}H_{82}O_{20}$	978.5399	PPT
434	notoginsenoside E	$C_{48}H_{82}O_{20}$	978.5399	PPD
435	yesanchinoside B*	$C_{48}H_{82}O_{20}$	978.5399	OT
436	ginsenoside S_3	$C_{48}H_{84}O_{20}$	980.5556	PPT
437	vinaginsenoside R13	$C_{48}H_{84}O_{20}$	980.5556	PPD
438	taibaienoside II*	$C_{49}H_{78}O_{18}$	954.5188	OA
439	pseudoginsenoside Rt1 ethyl ester	$C_{49}H_{78}O_{18}$	954.5188	OA
440	chikusetsusaponinV methyl ester*	$C_{49}H_{78}O_{19}$	970.5137	OA
441	chikusetsusaponinV ethyl ester*	$C_{50}H_{80}O_{19}$	984.5294	OA
442	malonylginsenoside Re4	$C_{50}H_{82}O_{21}$	1018.5349	PPT
443	acetylginsenoside Re*	$C_{50}H_{84}O_{19}$	988.5607	PPT
444	pseudoginsenoside Rc1	$C_{50}H_{84}O_{19}$	988.5607	PPD
445	pseudoginsenoside Rs1*	$C_{50}H_{84}O_{19}$	988.5607	PPT
446	quinquenoside III	$C_{50}H_{84}O_{19}$	988.5607	PPD
447	acetylginsenoside Rg3	$C_{50}H_{84}O_{20}$	1004.5556	PPT
448	floralginsenoside G	$C_{50}H_{84}O_{21}$	1020.5505	PPD
449	floralginsenoside H	$C_{50}H_{84}O_{21}$	1020.5505	PPT
450	pseudoginsenoside Rt1 butyl ester*	$C_{51}H_{82}O_{18}$	982.5501	OA
451	taibaienoside I*	$C_{51}H_{82}O_{18}$	982.5501	OA
452	epoxynotoginsenoside A	$C_{51}H_{84}O_{21}$	1032.5505	Others
453	malonylfloralginsenoside Re1*	$C_{51}H_{84}O_{21}$	1032.5505	PPT
454	malonylginsenoside Rd*	$C_{51}H_{84}O_{21}$	1032.5505	PPD
455	malonylfloralginsenoside Rd1	$C_{51}H_{84}O_{21}$	1032.5505	PPD
456	malonylfloralginsenoside Rd2	$C_{51}H_{84}O_{21}$	1032.5505	PPD
457	malonylfloralginsenoside Rd3	$C_{51}H_{84}O_{21}$	1032.5505	PPD
458	malonylfloralginsenoside Rd4	$C_{51}H_{84}O_{21}$	1032.5505	PPD
459	malonylfloralginsenoside Rd5	$C_{51}H_{84}O_{21}$	1032.5505	PPD
460	malonylfloralginsenoside Re2	$C_{51}H_{84}O_{21}$	1032.5505	PPT
461	malonylfloralginsenoside Re3	$C_{51}H_{84}O_{21}$	1032.5505	PPT
462	malonylginsenoside Rd6	$C_{51}H_{84}O_{21}$	1032.5505	PPD
463	malonylginsenoside Re	$C_{51}H_{84}O_{21}$	1032.5505	PPT
464	malonylginsenoside Re2	$C_{51}H_{84}O_{22}$	1048.5454	PPT
465	malonylginsenoside Re3	$C_{51}H_{84}O_{22}$	1048.5454	PPT
466	chikusetsusaponin Ⅴ butyl ester*	$C_{52}H_{84}O_{19}$	1012.5607	OA
467	3-O-{β-D-xylopyranosyl(1→3)-[α-l-arabinofuranosyl(1→4)]-β-D-glucopyranosyl}oleanolic acid 28-O-β-D-glucopyranosyl ester	$C_{52}H_{84}O_{21}$	1044.5505	Miscellaneous
468	ginsenoside Rh_{26}	$C_{52}H_{86}O_{19}$	1014.5763	PPD
469	quinquenoside I	$C_{52}H_{86}O_{19}$	1014.5763	PPD
470	ginsenoside Rh_{25}	$C_{52}H_{88}O_{21}$	1048.5818	PPD
471	gypenoside XV	$C_{52}H_{88}O_{21}$	1048.5818	PPD
472	notoginsenoside O	$C_{52}H_{88}O_{21}$	1048.5818	PPD
473	notoginsenoside P	$C_{52}H_{88}O_{21}$	1048.5818	PPD
474	chikusetsusaponin VII*	$C_{53}H_{90}O_{22}$	1078.5924	PPD
475	bipinoside C	$C_{53}H_{84}O_{22}$	1072.5454	OA

476	Stipuleanoside R2*	$C_{53}H_{84}O_{23}$	1088.5403	OA
477	chikusetsusaponin FK3*	$C_{53}H_{88}O_{22}$	1076.5767	Miscellaneous
478	chikusetsusaponin FK6*	$C_{53}H_{88}O_{22}$	1076.5767	PPD
479	quinquefoloside Lb	$C_{53}H_{88}O_{22}$	1076.5767	PPD
480	notoginsenoside LK1	$C_{53}H_{88}O_{23}$	1092.5716	PPD
481	notoginsenoside Ng2	$C_{53}H_{88}O_{23}$	1092.5716	PPD
482	notoginsenoside NL-C3	$C_{53}H_{88}O_{23}$	1092.5716	PPD
483	yesanchinoside G*	$C_{53}H_{88}O_{23}$	1092.5716	Miscellaneous
484	floralginsenoside M*	$C_{53}H_{90}O_{22}$	1078.5924	PPT
485	floralginsenoside N*	$C_{53}H_{90}O_{22}$	1078.5924	PPT
486	floralquinquenoside E	$C_{53}H_{90}O_{22}$	1078.5924	PPT
487	ginsenoside Rb2*	$C_{53}H_{90}O_{22}$	1078.5924	PPD
488	ginsenoside Rb3*	$C_{53}H_{90}O_{22}$	1078.5924	PPD
489	ginsenoside Rc*	$C_{53}H_{90}O_{22}$	1078.5924	PPD
490	notoginsenoside L	$C_{53}H_{90}O_{22}$	1078.5924	PPD
491	vinaginsenoside R7*	$C_{53}H_{90}O_{22}$	1078.5924	PPD
492	20(*S*) sanchirhinoside A6	$C_{53}H_{90}O_{23}$	1094.5873	PPT
493	chikusetsusaponin LM5*	$C_{53}H_{90}O_{23}$	1094.5873	PPT
494	floralginsenoside P*	$C_{53}H_{90}O_{23}$	1094.5873	PPT
495	floranotoginsenoside A	$C_{53}H_{90}O_{23}$	1094.5873	PPD
496	floranotoginsenoside D	$C_{53}H_{90}O_{23}$	1094.5873	PPD
497	gypenoside LXXI	$C_{53}H_{90}O_{23}$	1094.5873	PPD
498	notoginsenoside LK6	$C_{53}H_{90}O_{23}$	1094.5873	PPD
499	notoginsenoside LK7	$C_{53}H_{90}O_{23}$	1094.5873	PPD
500	yesanchinoside H*	$C_{53}H_{90}O_{23}$	1094.5873	PPD
501	floralginsenoside O	$C_{53}H_{90}O_{24}$	1110.5822	PPD
502	floralginsenoside Tc	$C_{53}H_{90}O_{24}$	1110.5822	PPD
503	floralginsenoside Td	$C_{53}H_{90}O_{24}$	1110.5822	PPD
504	floranotoginsenoside B	$C_{53}H_{90}O_{24}$	1110.5822	PPD
505	floranotoginsenoside C	$C_{53}H_{90}O_{24}$	1110.5822	PPD
506	chikusetsusaponin FM1*	$C_{53}H_{92}O_{24}$	1112.5979	PPD
507	notoginsenosides NL-E2	$C_{53}H_{92}O_{24}$	1112.5979	PPD
508	notoginsenosides NL-F1	$C_{53}H_{92}O_{24}$	1112.5979	PPD
509	stipuleanoside R2 methyl ester	$C_{54}H_{86}O_{23}$	1102.5560	OA
510	malonylfloralginsenoside Rd6*	$C_{54}H_{86}O_{24}$	1118.5509	PPD
511	5,6-didehydroginsenoside Rb1	$C_{54}H_{90}O_{23}$	1106.5873	Miscellaneous
512	notoginsenoside B*	$C_{54}H_{90}O_{24}$	1122.5822	PPD
513	quinquenoside IV	$C_{54}H_{90}O_{24}$	1122.5822	Miscellaneous
514	notoginsenoside I	$C_{54}H_{92}O_{22}$	1092.608	Miscellaneous
515	ginsenoside B1*	$C_{54}H_{92}O_{23}$	1108.6029	PPD
516	ginsenoside Rb1*	$C_{54}H_{92}O_{23}$	1108.6029	PPD
517	ginsenoside Re8*	$C_{54}H_{92}O_{23}$	1108.6029	PPT
518	quinquefoloside La	$C_{54}H_{92}O_{23}$	1108.6029	PPD
519	quinquefoloside Lc	$C_{54}H_{92}O_{23}$	1108.6029	PPD
520	quinquenoside Ja	$C_{54}H_{92}O_{23}$	1108.6029	PPT
521	yesanchinoside E*	$C_{54}H_{92}O_{23}$	1108.6029	PPT
522	ginsenoside Rb4	$C_{54}H_{92}O_{23}$	1108.6029	PPD
523	6-O-[β-D-glucopyranosyl-(1→2)-β-D-glucopyranosyl]-20-O-[β-D-glucopyranosyl-(1→4)-β-D-glucopyranosyl]-20(S) protopanaxatriol*	$C_{54}H_{92}O_{24}$	1124.5979	PPT
524	ginsenoside V	$C_{54}H_{92}O_{24}$	1124.5979	PPD
525	koryoginenoside R2	$C_{54}H_{92}O_{24}$	1124.5979	PPD
526	notoginsenoside A	$C_{54}H_{92}O_{24}$	1124.5979	PPD
527	notoginsenoside C	$C_{54}H_{92}O_{25}$	1140.5928	PPD

528	ursan-3α,11β-diol-3-O-α-D-glucopyranosyl-(6′→1″)-α-D-glucopyranosyl-(6″→1‴)-α-D-glucopyranosyl-(6‴→1⁗)-α-D-glucopyranoside	$C_{54}H_{93}O_{22}$	1093.6158	Miscellaneous
529	quinquenoside L16	$C_{54}H_{94}O_{25}$	1142.6084	PPD
530	ginsenoside Rs_1*	$C_{55}H_{92}O_{23}$	1120.6029	PPD
531	ginsenoside Rs_{11}	$C_{55}H_{92}O_{23}$	1120.6029	PPD
532	ginsenoside Rs_2*	$C_{55}H_{92}O_{23}$	1120.6029	PPD
533	pseudoginsenoside F8	$C_{55}H_{92}O_{23}$	1120.6029	PPD
534	malonylfloralginsenoside Rc1	$C_{56}H_{92}O_{25}$	1164.5928	PPD
535	malonylfloralginsenoside Rc2	$C_{56}H_{92}O_{25}$	1164.5928	PPD
536	malonylfloralginsenoside Rc3	$C_{56}H_{92}O_{25}$	1164.5928	PPD
537	malonylfloralginsenoside Rc4	$C_{56}H_{92}O_{25}$	1164.5928	PPD
538	malonylginsenoside Rb2	$C_{56}H_{92}O_{25}$	1164.5928	PPD
539	malonylginsenoside Rb3*	$C_{56}H_{92}O_{25}$	1164.5928	PPD
540	malonylginsenoside Rc	$C_{56}H_{92}O_{25}$	1164.5928	PPD
541	6″-O-acetylginsenoside Rb1	$C_{56}H_{94}O_{24}$	1150.6135	PPD
542	quinquenoside R1*	$C_{56}H_{94}O_{24}$	1150.6135	PPD
543	yesanchinoside F*	$C_{56}H_{94}O_{24}$	1150.6135	PPT
544	ginsenoside Ra_7	$C_{57}H_{94}O_{23}$	1146.6186	PPD
545	ginsenoside Ra_8	$C_{57}H_{94}O_{23}$	1146.6186	PPD
546	ginsenoside Ra_9	$C_{57}H_{94}O_{23}$	1146.6186	PPD
547	malonylfloralginsenoside Rb1	$C_{57}H_{94}O_{26}$	1194.6033	PPD
548	malonylfloralginsenoside Rb2	$C_{57}H_{94}O_{26}$	1194.6033	PPD
549	malonylginsenoside Rb1*	$C_{57}H_{94}O_{26}$	1194.6033	PPD
550	malonylginsenoside Re8	$C_{57}H_{94}O_{26}$	1194.6033	PPT
551	notoginsenoside NL-J	$C_{57}H_{94}O_{27}$	1210.5982	PPD
552	notoginsenoside Fc*	$C_{58}H_{98}O_{26}$	1210.6346	PPD
553	notoginsenosides NL-E_3	$C_{58}H_{100}O_{28}$	1244.6401	PPD
554	notoginsenosides NL-E_4	$C_{58}H_{100}O_{28}$	1244.6401	PPD
555	notoginsenosides NL-F_2	$C_{58}H_{100}O_{28}$	1244.6401	PPD
556	ginsenoside IV	$C_{58}H_{96}O_{24}$	1210.6346	PPD
557	ginsenoside Ra_6*	$C_{58}H_{96}O_{24}$	1210.6346	PPD
558	chikusetsusaponin FH_2*	$C_{58}H_{96}O_{26}$	1208.6190	Miscellaneous
559	chikusetsusaponin FT_3*	$C_{58}H_{96}O_{26}$	1208.6190	Others
560	notoginsenoside LK_2	$C_{58}H_{96}O_{26}$	1208.6190	PPD
561	notoginsenoside LK_4	$C_{58}H_{96}O_{27}$	1224.6139	PPD
562	notoginsenoside LK_5	$C_{58}H_{96}O_{27}$	1224.6139	PPD
563	ginsenoside Ra_1*	$C_{58}H_{98}O_{26}$	1210.6346	PPD
564	ginsenoside Ra_2*	$C_{58}H_{98}O_{26}$	1210.6346	PPD
565	notoginsenoside FP_2	$C_{58}H_{98}O_{26}$	1210.6346	PPD
566	notoginsenoside Fz	$C_{58}H_{98}O_{26}$	1210.6346	PPD
567	notoginsenoside NL-G_2	$C_{58}H_{98}O_{27}$	1226.6295	PPD
568	chikusetsusaponin LM_6*	$C_{58}H_{98}O_{27}$	1226.6295	PPT
569	notoginsenoside NL-B_2	$C_{58}H_{98}O_{27}$	1226.6295	PPD
570	notoginsenoside NL-B_3	$C_{58}H_{98}O_{27}$	1226.6295	PPD
571	notoginsenoside NL-H_3	$C_{58}H_{98}O_{27}$	1226.6295	PPD
572	notoginsenoside NL-A_3	$C_{58}H_{98}O_{28}$	1242.6245	PPD
573	chikusetsusaponin VI*	$C_{59}H_{100}O_{27}$	1240.6452	PPD
574	baisanqisaponin A*	$C_{59}H_{90}O_{16}$	1054.6229	OA
575	baisanqisaponin C*	$C_{59}H_{90}O_{16}$	1054.6229	OA
576	yesanchinoside I*	$C_{59}H_{100}O_{26}$	1224.6503	DD- II
577	ginsenoside Ra_3	$C_{59}H_{100}O_{27}$	1240.6452	PPD
578	notoginsenoside Fa*	$C_{59}H_{100}O_{27}$	1240.6452	PPD
579	notoginsenoside R4*	$C_{59}H_{100}O_{27}$	1240.6452	PPD

580	quinquenoside Jb	$C_{59}H_{100}O_{27}$	1240.6452	PPD
581	chikusetsusaponin FT_2*	$C_{59}H_{98}O_{27}$	1238.6295	Miscellaneous
582	notoginsenoside LK_3	$C_{59}H_{98}O_{27}$	1238.6295	PPD
583	ginsenoside Ra_5	$C_{60}H_{100}O_{27}$	1252.6452	PPD
584	ginsenoside Ra0	$C_{60}H_{102}O_{28}$	1270.6558	PPD
585	ginsenoside Rb_6	$C_{60}H_{102}O_{28}$	1270.6558	PPD
586	quinquenoside V	$C_{60}H_{102}O_{28}$	1270.6558	PPD
587	ginsenoside Rb_5	$C_{60}H_{102}O_{28}$	1270.6558	PPD
588	yesanchinoside J*	$C_{61}H_{102}O_{28}$	1282.6558	PPD
589	malonylginsenoside Ra1*	$C_{61}H_{100}O_{29}$	1296.6350	PPD
590	malonylginsenoside Ra2*	$C_{61}H_{100}O_{29}$	1296.6350	PPD
591	malonylginsenoside Ra6	$C_{61}H_{98}O_{27}$	1262.6295	PPD
592	malonylnotoginsenoside R4	$C_{62}H_{101}O_{30}$	1325.6378	PPD
593	ginsenoside Ra_4	$C_{62}H_{102}O_{27}$	1278.6608	PPD
594	malonylginsenoside Ra3*	$C_{62}H_{102}O_{30}$	1326.6456	PPD
595	quinquenoside II	$C_{62}H_{104}O_{24}$	1232.6918	PPD
596	notoginsenoside Q*	$C_{63}H_{106}O_{30}$	1342.6769	PPD
597	notoginsenoside S	$C_{63}H_{106}O_{30}$	1342.6769	PPD
598	notoginsenoside D	$C_{64}H_{108}O_{31}$	1372.6875	PPD
599	notoginsenoside T	$C_{64}H_{108}O_{31}$	1372.6875	PPD
600	baisanqisaponin B*	$C_{64}H_{98}O_{20}$	1186.6651	OA
601	polyacetyleneginsenoside Ro*	$C_{65}H_{100}O_{21}$	1216.6757	OA
602	(cis)-7β,11α,19,21-tetra-O-decanoy-18,22β-dihydroxy-dammar-1-en-3-one	$C_{70}H_{122}O_{11}$	1138.8987	Others
603	chikusetsusaponin FK4*	$C_{58}H_{98}O_{26}$	1210.6346	PPD
604	chikusetsusaponin LT8*	$C_{42}H_{70}O_{13}$	782.4816	Miscellaneous
605	chikusetsusaponin LT5*	$C_{48}H_{80}O_{18}$	944.5345	Miscellaneous
606	chikusetsusaponin LN4*	$C_{52}H_{86}O_{21}$	1046.5662	Miscellaneous
607	chikusetsusaponin FK5*	$C_{58}H_{98}O_{26}$	1210.6346	PPD
608	gypenoside LVI*	$C_{53}H_{90}O_{23}$	1094.5873	PPD
609	gypenoside LXVII*	$C_{53}H_{90}O_{23}$	1094.5873	PPD
610	gypenoside XVII*	$C_{48}H_{82}O_{18}$	946.5501	PPD
611	chikusetsusaponin $L9_{bc}$*	$C_{36}H_{62}O_{10}$	654.4343	PPT
612	ginsenoside F6*	$C_{47}H_{80}O_{18}$	932.5345	PPT

* The compound reported in *Panacis Japonici.*

Table S7. 228 predicted saponin metabolites of *Panacis Japonici*.

No.	Compound	Modofied groups	Molecular formula	Exact mass
1	ginsenoside Rh1	Mal	$C_{39}H_{64}O_{12}$	724.4398
2	20-glc-ginsenoside-Rf	Mal	$C_{45}H_{74}O_{17}$	886.4926
3	6-O-[β-D-glucopyranosyl-(1→2)-β-D-glucopyranosyl]-20-O-[β-D-glucopyranosyl-(1→4)-β-D-glucopyranosyl]-20(s)-protopanaxatriol	Ac	$C_{56}H_{94}O_{25}$	1166.6084
4		Mal	$C_{57}H_{94}O_{27}$	1210.5982
5	baisanqisaponin A	Me	$C_{60}H_{92}O_{16}$	1068.6385
6		Et	$C_{61}H_{94}O_{16}$	1082.6542
7		Bu	$C_{63}H_{98}O_{16}$	1110.6855
8	baisanqisaponin B	Me	$C_{65}H_{100}O_{20}$	1200.6808
9		Et	$C_{66}H_{102}O_{20}$	1214.6964
10		Bu	$C_{68}H_{106}O_{20}$	1242.7277
11	baisanqisaponin C	Me	$C_{60}H_{92}O_{16}$	1068.6385
12		Et	$C_{61}H_{94}O_{16}$	1082.6542
13		Bu	$C_{63}H_{98}O_{16}$	1110.6855
14	chikusetsusaponin FH1	Ac	$C_{43}H_{70}O_{14}$	810.4766
15		Mal	$C_{44}H_{70}O_{16}$	854.4664
16	chikusetsusaponin FH2	Ac	$C_{60}H_{98}O_{27}$	1250.6295
17		Mal	$C_{61}H_{98}O_{29}$	1294.6194
18	chikusetsusaponin FK1	Ac	$C_{50}H_{84}O_{19}$	988.5607
19		Mal	$C_{51}H_{84}O_{21}$	1032.5505
20	chikusetsusaponin FK2	Ac	$C_{50}H_{82}O_{19}$	986.5450
21		Mal	$C_{51}H_{82}O_{21}$	1030.5349
22	chikusetsusaponin FK3	Ac	$C_{55}H_{90}O_{23}$	1118.5873
23		Mal	$C_{56}H_{90}O_{25}$	1162.5771
24	chikusetsusaponin FK4	Ac	$C_{60}H_{100}O_{27}$	1252.6452
25		Mal	$C_{61}H_{100}O_{29}$	1296.6350
26	chikusetsusaponin FK5	Ac	$C_{60}H_{100}O_{27}$	1252.6452
27		Mal	$C_{61}H_{100}O_{29}$	1296.6350
28	chikusetsusaponin FK6	Ac	$C_{55}H_{90}O_{23}$	1118.5873
29		Mal	$C_{56}H_{90}O_{25}$	1162.5771
30	chikusetsusaponin FK7	Ac	$C_{50}H_{84}O_{19}$	988.5607
31		Mal	$C_{51}H_{84}O_{21}$	1032.5505
32	chikusetsusaponin FM1	Ac	$C_{55}H_{94}O_{25}$	1154.6084
33		Mal	$C_{56}H_{94}O_{27}$	1198.5982
34	chikusetsusaponin FT1	Ac	$C_{43}H_{70}O_{14}$	810.4766
35		Mal	$C_{44}H_{70}O_{16}$	854.4664
36	chikusetsusaponin FT2	Ac	$C_{61}H_{100}O_{28}$	1280.6401
37		Mal	$C_{62}H_{100}O_{30}$	1324.6299
38	chikusetsusaponin FT3	Ac	$C_{60}H_{98}O_{27}$	1250.6295
39		Mal	$C_{61}H_{96}O_{29}$	1292.6037
40	chikusetsusaponin FT4	Ac	$C_{49}H_{80}O_{18}$	956.5345
41		Mal	$C_{50}H_{80}O_{20}$	1000.5243
42	chikusetsusaponin Ia	Ac	$C_{43}H_{72}O_{13}$	796.4973
43		Mal	$C_{44}H_{72}O_{15}$	840.4871
44	chikusetsusaponin Ib	Mt	$C_{48}H_{76}O_{18}$	940.5032
45		Et	$C_{49}H_{78}O_{18}$	954.5188
46		Bu	$C_{51}H_{82}O_{18}$	982.5501
47	chikusetsusaponin II	Mt	$C_{43}H_{68}O_{14}$	808.4609
48		Et	$C_{44}H_{70}O_{14}$	822.4766

49		Bu	$C_{46}H_{74}O_{14}$	850.5079
50	chikusetsusaponin III	Ac	$C_{49}H_{82}O_{18}$	958.5501
51		Mal	$C_{50}H_{82}O_{20}$	1002.5399
52	chikusetsusaponin L10	Ac	$C_{38}H_{64}O_{10}$	680.4499
53		Mal	$C_{39}H_{64}O_{12}$	724.4398
54	chikusetsusaponin L_5	Ac	$C_{48}H_{80}O_{18}$	944.5345
55		Mal	$C_{49}H_{80}O_{20}$	988.5243
56	chikusetsusaponin $L9_a$	Ac	$C_{38}H_{64}O_{11}$	696.4449
57		Mal	$C_{39}H_{64}O_{13}$	740.4347
58	chikusetsusaponin $L9_{bc}$	Ac	$C_{38}H_{64}O_{11}$	696.4449
59		Mal	$C_{39}H_{64}O_{13}$	740.4347
60	chikusetsusaponin LM1	Ac	$C_{43}H_{72}O_{14}$	812.4922
61		Mal	$C_{44}H_{72}O_{16}$	856.4820
62	chikusetsusaponin LM2	Ac	$C_{48}H_{80}O_{18}$	944.5345
63		Mal	$C_{49}H_{80}O_{20}$	988.5243
64	chikusetsusaponin LM3	Ac	$C_{48}H_{80}O_{18}$	944.5345
65		Mal	$C_{49}H_{80}O_{20}$	988.5243
66	chikusetsusaponin LM4	Ac	$C_{50}H_{84}O_{20}$	1004.5556
67		Mal	$C_{51}H_{84}O_{22}$	1048.5454
68	chikusetsusaponin LM5	Ac	$C_{55}H_{92}O_{24}$	1136.5979
69		Mal	$C_{56}H_{92}O_{26}$	1180.5877
70	chikusetsusaponin LM6	Ac	$C_{60}H_{100}O_{28}$	1268.6401
71		Mal	$C_{61}H_{100}O_{30}$	1312.6299
72	chikusetsusaponin LN4	Ac	$C_{54}H_{88}O_{22}$	1088.5767
73		Mal	$C_{55}H_{88}O_{24}$	1132.5666
74	chikusetsusaponin LT5	Ac	$C_{50}H_{82}O_{19}$	986.5450
75		Mal	$C_{51}H_{82}O_{21}$	1030.5349
76	chikusetsusaponin LT8	Ac	$C_{44}H_{72}O_{14}$	824.4922
77		Mal	$C_{45}H_{72}O_{16}$	868.4820
78	chikusetsusaponin VI	Ac	$C_{61}H_{102}O_{28}$	1282.6558
79		Mal	$C_{62}H_{102}O_{30}$	1326.6456
80	chikusetsusaponin VII	Ac	$C_{55}H_{92}O_{23}$	1120.6029
81		Mal	$C_{56}H_{92}O_{25}$	1164.5928
82	ginsenoside K	Ac	$C_{38}H_{64}O_{9}$	664.4550
83		Mal	$C_{39}H_{64}O_{11}$	708.4449
84	ginsenoside B1	Ac	$C_{56}H_{94}O_{24}$	1150.6135
85		Mal	$C_{57}H_{94}O_{26}$	1194.6033
86	ginsenoside F2	Ac	$C_{44}H_{74}O_{14}$	826.5079
87		Mal	$C_{45}H_{74}O_{16}$	870.4977
88	ginsenoside F3	Ac	$C_{43}H_{72}O_{14}$	812.4922
89		Mal	$C_{44}H_{72}O_{16}$	856.4820
90	ginsenoside F4	Ac	$C_{44}H_{72}O_{13}$	808.4973
91		Mal	$C_{45}H_{72}O_{15}$	852.4871
92	ginsenoside F5	Ac	$C_{43}H_{72}O_{14}$	812.4922
93		Mal	$C_{44}H_{72}O_{16}$	856.4820
94	ginsenoside F6	Ac	$C_{49}H_{82}O_{19}$	974.5450
95		Mal	$C_{50}H_{82}O_{21}$	1018.5349
96	ginsenoside III	Ac	$C_{50}H_{82}O_{20}$	1002.5399
97		Mal	$C_{51}H_{82}O_{22}$	1046.5298
98	ginsenoside Ra6	Ac	$C_{60}H_{98}O_{25}$	1218.6397

99	ginsenoside Rb2	Ac	$C_{55}H_{92}O_{23}$	1120.6029
100	ginsenoside Rc	Ac	$C_{55}H_{92}O_{24}$	1136.5979
101	ginsenoside Re2	Ac	$C_{50}H_{84}O_{20}$	1004.5556
102	ginsenoside Re3	Ac	$C_{50}H_{84}O_{20}$	1004.5556
103	ginsenoside Re4	Ac	$C_{49}H_{82}O_{19}$	974.5450
104	ginsenoside Re8	Ac	$C_{56}H_{94}O_{24}$	1150.6135
105	ginsenoside Rg2	Ac	$C_{44}H_{74}O_{14}$	826.5079
106	ginsenoside Rg5	Ac	$C_{44}H_{72}O_{13}$	808.4973
107	ginsenoside Rg6	Ac	$C_{44}H_{72}O_{13}$	808.4973
108	ginsenoside Rg9	Ac	$C_{44}H_{72}O_{14}$	824.4922
109		Mal	$C_{45}H_{72}O_{16}$	868.4820
110	ginsenoside Rh2	Ac	$C_{38}H_{64}O_{9}$	664.4550
111		Mal	$C_{39}H_{64}O_{11}$	708.4449
112	ginsenoside Rh4	Ac	$C_{38}H_{62}O_{9}$	662.4394
113		Mal	$C_{39}H_{62}O_{11}$	706.4292
114	ginsenoside Rk1	Ac	$C_{44}H_{72}O_{13}$	808.4973
115	ginsenoside Rk2	Ac	$C_{38}H_{62}O_{8}$	646.4445
116	ginsenoside Rk3	Ac	$C_{38}H_{62}O_{9}$	662.4394
117	ginsenoside Rs1	Mal	$C_{58}H_{94}O_{26}$	1206.6033
118	ginsenoside Rs2	Mal	$C_{58}H_{94}O_{26}$	1206.6033
119	ginsenoside Rs3	Mal	$C_{47}H_{76}O_{17}$	912.5083
120	ginsenoside Rs5	Mal	$C_{47}H_{74}O_{16}$	894.4977
121	gypenoside LVI	Ac	$C_{55}H_{92}O_{24}$	1136.5979
122		Mal	$C_{56}H_{92}O_{26}$	1180.5877
123	gypenoside LXVII	Ac	$C_{55}H_{92}O_{24}$	1136.5979
124		Mal	$C_{56}H_{92}O_{26}$	1180.5877
125	gypenoside XVII	Ac	$C_{50}H_{94}O_{19}$	998.6389
126		Mal	$C_{51}H_{84}O_{21}$	1032.5505
127	hemsgiganoside B	Me	$C_{49}H_{78}O_{19}$	970.5137
128		Et	$C_{50}H_{80}O_{19}$	984.5294
129		Bu	$C_{52}H_{84}O_{19}$	1012.5607
130	majoroside F2	Ac	$C_{44}H_{74}O_{15}$	842.5028
131		Mal	$C_{45}H_{74}O_{17}$	886.4926
132	majoroside F3	Ac	$C_{44}H_{74}O_{15}$	842.5028
133		Mal	$C_{45}H_{74}O_{17}$	886.4926
134	majoroside F4	Ac	$C_{44}H_{74}O_{15}$	842.5028
135		Mal	$C_{45}H_{74}O_{17}$	886.4926
136	maslinic Acid 28-O-β-d-glucopyranoside	Me	$C_{37}H_{60}O_{9}$	648.4237
137		Et	$C_{38}H_{62}O_{9}$	662.4394
138		Bu	$C_{40}H_{66}O_{9}$	690.4707
139	notoginsenoside B	Ac	$C_{56}H_{92}O_{25}$	1164.5928
140	notoginsenoside Fa	Ac	$C_{61}H_{102}O_{28}$	1282.6558
141		Mal	$C_{59}H_{100}O_{30}$	1288.6299
142	notoginsenoside Fc	Ac	$C_{60}H_{100}O_{27}$	1252.6452
143		Mal	$C_{61}H_{100}O_{29}$	1296.6350
144	notoginsenoside Fe	Ac	$C_{49}H_{82}O_{18}$	958.5501
145		Mal	$C_{50}H_{82}O_{20}$	1002.5399
146	notoginsenoside Fp1	Ac	$C_{49}H_{82}O_{19}$	974.5450
147		Mal	$C_{50}H_{82}O_{21}$	1018.5349
148	notoginsenoside G	Ac	$C_{50}H_{82}O_{20}$	1002.5399
149		Mal	$C_{51}H_{82}O_{22}$	1046.5298

150	notoginsenoside J	Ac	$C_{44}H_{76}O_{17}$	876.5083
151		Mal	$C_{45}H_{76}O_{19}$	920.4981
152	notoginsenoside K	Ac	$C_{50}H_{84}O_{19}$	988.5607
153		Mal	$C_{51}H_{84}O_{21}$	1032.5505
154	notoginsenoside M	Ac	$C_{50}H_{84}O_{20}$	1004.5556
155		Mal	$C_{51}H_{84}O_{22}$	1048.5454
156	notoginsenoside N	Ac	$C_{50}H_{84}O_{20}$	1004.5556
157		Mal	$C_{51}H_{84}O_{22}$	1048.5454
158	notoginsenoside Q	Ac	$C_{65}H_{108}O_{31}$	1384.6875
159		Mal	$C_{66}H_{108}O_{33}$	1428.6773
160	notoginsenoside R1	Ac	$C_{49}H_{82}O_{19}$	974.5450
161		Mal	$C_{50}H_{82}O_{21}$	1018.5349
162	notoginsenoside R2	Ac	$C_{43}H_{72}O_{14}$	812.4922
163		Mal	$C_{44}H_{72}O_{16}$	856.4820
164	notoginsenoside R3	Ac	$C_{50}H_{84}O_{20}$	1004.5556
165		Mal	$C_{51}H_{84}O_{22}$	1048.5454
166	notoginsenoside R4	Ac	$C_{61}H_{102}O_{28}$	1282.6558
167	notoginsenoside R6	Ac	$C_{50}H_{84}O_{20}$	1004.5556
168		Mal	$C_{51}H_{84}O_{23}$	1064.5403
169	notoginsenoside T5	Ac	$C_{43}H_{70}O_{13}$	794.4816
170		Mal	$C_{44}H_{70}O_{16}$	854.4664
171	oleanolic Acid 28-O-β-D-glucopyranose	Mt	$C_{37}H_{60}O_{8}$	632.4288
172		Et	$C_{38}H_{62}O_{8}$	646.4445
173		Bu	$C_{40}H_{66}O_{8}$	674.4758
174	pjs-2	Mt	$C_{48}H_{76}O_{18}$	940.5032
175		Et	$C_{49}H_{78}O_{18}$	954.5188
176		Bu	$C_{51}H_{82}O_{18}$	982.5501
177	pjs-4	Mt	$C_{42}H_{68}O_{12}$	764.4711
178		Et	$C_{43}H_{70}O_{12}$	778.4867
179		Bu	$C_{45}H_{74}O_{12}$	806.5180
180	prosaikogenin H	Ac	$C_{38}H_{60}O_{9}$	660.4237
181		Mal	$C_{39}H_{60}O_{11}$	704.4136
182	pseudoginsenoside Rp1	Mt	$C_{42}H_{66}O_{13}$	778.4503
183		Et	$C_{43}H_{68}O_{13}$	792.4660
184		Bu	$C_{45}H_{72}O_{13}$	820.4973
185	pseudoginsenoside Rs1	Ac	$C_{52}H_{86}O_{20}$	1030.5712
186		Mal	$C_{53}H_{86}O_{22}$	1074.5611
187	quinquenoside L1	Ac	$C_{50}H_{82}O_{19}$	986.5450
188		Mal	$C_{51}H_{82}O_{21}$	1030.5349
189	quinquenoside L17	Ac	$C_{49}H_{82}O_{19}$	974.5450
190		Mal	$C_{50}H_{82}O_{21}$	1018.5349
191	quinquenoside R1	Ac	$C_{58}H_{96}O_{25}$	1192.6241
192		Mal	$C_{59}H_{96}O_{27}$	1236.6139
193	sanchirhinoside A3	Ac	$C_{43}H_{72}O_{14}$	812.4922
194		Mal	$C_{44}H_{72}O_{16}$	856.4820
195	sanchirhinoside A4	Ac	$C_{43}H_{72}O_{14}$	812.4922
196		Mal	$C_{44}H_{72}O_{16}$	856.4820
197	stipuleanoside R1	Me	$C_{48}H_{76}O_{18}$	940.5032
198		Et	$C_{49}H_{78}O_{18}$	954.5188
199		Bu	$C_{51}H_{82}O_{18}$	982.5501
200	stipuleanoside R2	Et	$C_{55}H_{88}O_{18}$	1036.5971

201		Bu	$C_{57}H_{92}O_{23}$	1144.6029
202		Me	$C_{52}H_{84}O_{18}$	996.5658
203	taibaienoside I	Et	$C_{53}H_{86}O_{18}$	1010.5814
204		Bu	$C_{55}H_{90}O_{18}$	1038.6127
205		Me	$C_{50}H_{80}O_{18}$	968.5345
206	taibaienoside II	Et	$C_{51}H_{82}O_{18}$	982.5501
207		Bu	$C_{53}H_{86}O_{18}$	1010.5814
208		Me	$C_{49}H_{78}O_{19}$	970.5137
209	tuberoside A	Et	$C_{50}H_{80}O_{19}$	984.5294
210		Bu	$C_{52}H_{84}O_{19}$	1012.5607
211	yesanchinoside D	Ac	$C_{46}H_{76}O_{16}$	884.5133
212		Mal	$C_{47}H_{76}O_{18}$	928.5032
213	yesanchinoside E	Ac	$C_{56}H_{94}O_{24}$	1150.6135
214		Mal	$C_{57}H_{94}O_{26}$	1194.6033
215	yesanchinoside f	Ac	$C_{58}H_{96}O_{25}$	1192.6241
216		Mal	$C_{59}H_{96}O_{27}$	1236.6139
217	yesanchinoside G	Ac	$C_{55}H_{90}O_{24}$	1134.5822
218		Mal	$C_{56}H_{90}O_{26}$	1178.5720
219	yesanchinoside H	Ac	$C_{55}H_{92}O_{24}$	1136.5979
220		Mal	$C_{56}H_{92}O_{26}$	1180.5877
221	yesanchinoside J	Ac	$C_{63}H_{104}O_{29}$	1324.6663
222		Mal	$C_{64}H_{104}O_{31}$	1368.6562
223	yesanchinoside R1	Ac	$C_{38}H_{64}O_{11}$	696.4449
224		Mal	$C_{39}H_{64}O_{13}$	740.4347
225	yesanchinoside R2	Ac	$C_{43}H_{72}O_{15}$	828.4871
226		Mal	$C_{44}H_{72}O_{17}$	872.477
227	yesanchinoside R3	Ac	$C_{38}H_{64}O_{11}$	696.4449
228		Mal	$C_{39}H_{64}O_{13}$	740.4347

Mal: malonyl; Ac: acetyl; Me: methyl; Et: ethyl; Bu: buthyl

Table S8. Detailed structural information of the 307 saponins characterized by 2D LC-MS.

No.	t_R (min)	Adduct	Observed *m/z*	Theoretical *m/z*	Formula	Error (ppm)	Identification	Type	ESI-MS^2	Origin
1	2.87	$[M + HCOO]^-$	1025.5518	979.5478	$C_{48}H_{84}O_{20}$	5.5	C4-Glc-Rha-Glc	C4	979.5596, 799.4845,781.4490, 653.4387, 509.3967	F14
2	3.09	$[M + HCOO]^-$	1025.5588	979.5478	$C_{48}H_{84}O_{20}$	5.5	C3-Glc-Rha-Glc	C3	979.5596, 799.4845,709.9676, 653.4387, 509.3869, 491.3747	F14
3	3.91	$[M + HCOO]^-$	993.5362	947.5216	$C_{47}H_{80}O_{19}$	-5.7	D1-Glc-Rha-Glc	D1	947.5162, 767.4493, 621.4021, 477.3530	F13
4	4.11	$[M + HCOO]^-$	879.4962	833.4899	$C_{42}H_{74}O_{16}$	0.4	notoginsenoside J or isomer	C4	833.4902, 671.4362, 509.3837	F13
5	4.21	$[M + HCOO]^-$	1007.5425	961.5372	$C_{48}H_{82}O_{19}$	-0.2	(PPT+O)-Glc-Rha-Glc	PPT+O	961.5474,799.4845,781.4734,653.4164,635 .4139,565.3687,491.3651	F13
6	4.28	$[M + HCOO]^-$	1009.5534	963.5529	$C_{48}H_{84}O_{19}$	-4.9	(PPT+H_2O)-Glc-Rha-Glc	PPT+H_2O	963.5662, 801.4984, 783.5012, 655.4607, 637.4300, 493.3903, 475.3801	F10
7	4.54	$[M + HCOO]^-$	879.4957	833.4899	$C_{42}H_{74}O_{16}$	-0.2	notoginsenoside J or isomer	C4	833.4893, 671.4369, 653.4259, 509.3843, 391.2848	F13
8	4.66	$[M + HCOO]^-$	1023.5321	977.5321	$C_{48}H_{82}O_{20}$	-5.4	yesanchinoside B	OT	977.5378, 815.4796, 653.4276, 491.3747,415.3195	F14
9	4.97	$[M + HCOO]^-$	993.5225	947.5216	$C_{47}H_{80}O_{19}$	-4.5	C1-Glc-Glc-Xyl	C1	947.5162, 815.4671, 653.4276, 491.3747,391.2049	F13
10	5.27	$[M + HCOO]^-$	849.4843	803.4793	$C_{41}H_{72}O_{15}$	-0.6	C4-Glc-Xyl	C4	803.4777, 671.4326, 653.4276, 509.3869,491.1908,391.2929	F10
11	5.32	$[M + HCOO]^-$	993.5225	947.5216	$C_{47}H_{80}O_{19}$	-4.5	C1-Glc-Glc-Xyl	C1	947.5162, 815.4671, 653.4276, 491.3747,391.2740	F13
12	5.35	$[M + HCOO]^-$	1007.5414	961.5372	$C_{48}H_{82}O_{19}$	-1.9	C3-Glc-Rha-Glc	C3	961.5339, 799.4722, 653.4387, 509.3869,491.1908	F10
13	5.35	$[M + HCOO]^-$	993.5225	947.5216	$C_{47}H_{80}O_{19}$	-4.5	OT-Glc-Glc-Xyl	OT	947.5162, 815.4671, 635.4113, 491.3747,415.9409	F13
14	5.39	$[M + HCOO]^-$	863.5040	817.4949	$C_{42}H_{74}O_{15}$	4.2	C4-Glc-Rha	C4	817.4885,671.4326, 653.4276,509.3869, 391.2843	F9
15	5.47	$[M + HCOO]^-$	1007.5425	961.5372	$C_{48}H_{82}O_{19}$	-0.2	OT-Glc-Glc-Rha	OT	961.5380, 815.4795, 797.4684, 653.4269, 635.4156, 491.3732,415.3229	F12
16	5.97	$[M + HCOO]^-$	861.4862	815.4793	$C_{42}H_{72}O_{15}$	1.0	vinaginsenoside R15 or isomer	C3	815.4796, 653.4276, 491.3747, 391.2843	F10
17	6.13	$[M - H]^-$	961.5474	961.5372	$C_{48}H_{82}O_{19}$	5.2	C1-Glc-Rha-Glc	C1	961.5204, 799.4722, 735.0389, 653.4164, 491.3651, 391.2843	F8
18	6.25	$[M + HCOO]^-$	861.4839	815.4793	$C_{42}H_{72}O_{15}$	-1.7	vinaginsenoside R15 or isomer	C3	815.4796, 653.4276, 491.3747, 391.2843	F10
19	6.32	$[M + HCOO]^-$	831.4694	785.4687	$C_{41}H_{70}O_{14}$	-5.8	yesanchinoside R2	C1	785.4704, 553.3367,491.3651, 391.2843	F9

20	6.38	$[M + HCOO]^-$	1153.5614	1107.5587	$C_{53}H_{88}O_{24}$	2.5	(PPT+H_2O)-Glc-Glc-Rha-Rha	PPT+H_2O	1107.5470,961.5204,817.5010,655.4607,493.4903	F14
21	6.65	$[M + HCOO]^-$	831.4694	785.4687	$C_{41}H_{70}O_{14}$	-5.8	notoginsenoside Rw2 or isomer	C3	785.4704,653.4276, 491.3742, 403.3220, 391.2843	F8
22	6.80	$[M + HCOO]^-$	831.4743	785.4687	$C_{41}H_{70}O_{14}$	-0.6	notoginsenoside Rw2 or isomer	C3	785.4704, 653.4276, 491.3747, 391.2843	F8
23	7.11	$[M + HCOO]^-$	847.5053	801.5000	$C_{42}H_{74}O_{14}$	-0.7	ginsenoside Rf2	D3	801.5107, 655.4607, 493.3903	F17
24	7.16	$[M + HCOO]^-$	847.5079	801.5000	$C_{42}H_{74}O_{14}$	2.2	D3-Glc-Rha	D3	801.5107, 655.4607, 493.3903	F10
25	7.29	$[M + HCOO]^-$	1169.5906	1123.59	$C_{54}H_{92}O_{24}$	-4.2	(PPT+O)-Rha-Glc-Glc-Glc	PPT+O	1123.5908, 961.5339, 943.5012, 799.4845, 637.4300, 491.3747,391.3015	F14
26	7.39	$[M + HCOO]^-$	845.4926	799.4844	$C_{42}H_{72}O_{14}$	3.2	(PPT+O)-Glc-Rha	PPT+O	799.4845, 653.4276, 635.4250, 491.3747, 391.2843	F8
27	7.50	$[M + HCOO]^-$	1007.5415	961.5372	$C_{48}H_{82}O_{19}$	-1.7	notoginsenoside R6	PPT	961.5339, 799.4845, 637.4300, 475.3801	F13
28	7.54	$[M + HCOO]^-$	847.5079	801.5000	$C_{42}H_{74}O_{14}$	2.2	D3-Glc-Rha	D3	801.5231, 655.4495, 493.3903	F10
29	7.58	$[M + HCOO]^-$	845.4926	799.4844	$C_{42}H_{72}O_{14}$	3.2	(PPT+O)-Glc-Rha	PPT+O	799.4845, 653.4276, 635.4250, 491.3747, 391.2843	F8
30	7.66	$[M - H]^-$	973.5006	973.5008	$C_{48}H_{78}O_{20}$	-0.2	PPT-GlcA-Glc-Glc	PPT	973.5006, 811.4442, 749.4343, 631.3915, 475.3706	F11
31	7.78	$[M + HCOO]^-$	861.4861	815.4793	$C_{42}H_{72}O_{15}$	-1.2	floralquinquenoside B	D4	815.4783,669.4212,507.3681,491.3747	F8
32*	7.87	$[M + HCOO]^-$	977.5336	931.5266	$C_{47}H_{80}O_{18}$	0.9	notoginsenoside R1	PPT	931.5477, 799.5092, 637.4300, 619.4333, 475.3801	F10
33	8.01	$[M + HCOO]^-$	905.5154	905.5110	$C_{45}H_{78}O_{18}$	4.9	C4-Glc-Rha-Ac	C4	859.5049, 817.4885, 671.4439,653.4276, 509.3770,491.3747,391.2843	F7
34	8.08	$[M + HCOO]^-$	861.4841	815.4793	$C_{42}H_{72}O_{15}$	-1.4	majonoside R1	OT	815.4796, 653.4276, 491.3747, 415.3195	F10
35	8.09	$[M + HCOO]^-$	977.5335	931.5266	$C_{47}H_{80}O_{18}$	0.8	ginsenoside Re4	PPT	931.5477, 799.5092, 769.4710, 637.4300, 619.4333, 475.3801	F11
36	8.21	$[M + HCOO]^-$	861.4841	815.4793	$C_{42}H_{72}O_{15}$	-1.4	OT-Glc-Glc	OT	815.4796, 653.4276, 491.3747, 415.3195	F10
37	8.40	$[M + HCOO]^-$	989.5347	943.5266	$C_{48}H_{80}O_{18}$	2.6	B2-Glc-Glc-Rha	B2	943.5281, 763.4624, 635.4250,617.4135,473.3637	F14
38	8.45	$[M + HCOO]^-$	977.5320	931.5266	$C_{47}H_{80}O_{18}$	-0.7	notoginsenoside Fp1	PPT	931.5210, 799.4969, 637.4300, 619.4116, 475.3801	F10
39	8.46	$[M + HCOO]^-$	1007.543	961.5372	$C_{48}H_{82}O_{19}$	-0.2	notoginsenoside N or notoginsenoside M	PPT	961.5339, 799.4845, 637.4189, 475.3801	F12
40	8.54	$[M + HCOO]^-$	1005.5263	959.5216	$C_{48}H_{80}O_{19}$	-1.3	notoginsenoside G or isomer	B2	959.5217, 797.4672, 635.4145, 617.4135,473.3614	F13

41	8.83	[M + HCOO]⁻	989.5347	943.5266	$C_{48}H_{80}O_{18}$	2.6	B2-Glc-Glc-Rha	B2	943.5281, 797.4855, 635.4360,617.4135,473.3637	F14
42	8.99	[M + HCOO]⁻	1007.5425	961.5372	$C_{48}H_{82}O_{19}$	-0.2	PPT-Glc-Glc-Glc	PPT	961.5339, 799.4722, 637.4300,475.3801	F12
43*	9.01	[M + HCOO]⁻	845.4891	799.4844	$C_{42}H_{72}O_{14}$	-1.5	ginsenoside Rg1	PPT	799.4845, 637.4300, 475.3801, 391.2843	F8
44*	9.17	[M + HCOO]⁻	991.5477	945.5423	$C_{48}H_{82}O_{18}$	-0.6	ginsenoside Re	PPT	945.5406, 783.4866, 765.4765, 637.4296, 619.4193475.3769, 391.2842	F10
45	9.27	[M + HCOO]⁻	991.5510	945.5423	$C_{48}H_{82}O_{18}$	3.2	PPD-Glc-Rha-Glc	PPD	945.5373, 783.4734, 621.4456, 459.3879	F4
46	9.44	[M + HCOO]⁻	991.5481	945.5423	$C_{48}H_{82}O_{18}$	-0.2	PPT-Glc-Rha-Glc	PPT	945.5412,799.4835, 783.4890,765.4776, 637.4306,619.4201, 475.3775,391.2842	F11
47	9.58	[M + HCOO]⁻	991.5481	945.5423	$C_{48}H_{82}O_{18}$	-0.2	PPT-Glc-Rha-Glc	PPT	945.5412, 783.4890, 637.4300, 475.3801	F11
48	9.71	[M − H]⁻	943.4879	943.4903	$C_{47}H_{76}O_{19}$	-2.5	B2-GlcA-Glc-Xyl	B2	943.4879, 631.3806, 587.3945,473.3542	F9
49	9.76	[M + HCOO]⁻	831.474	785.4687	$C_{41}H_{70}O_{14}$	-0.9	majonoside R2 or isomer	OT	785.4704, 653.4276, 491.3747, 415.3195	F8
50	9.84	[M + HCOO]⁻	1025.5588	979.5478	$C_{48}H_{84}O_{20}$	5.5	(PPT+H_2O)-Glc-Glc-Glc	PPT+H_2O	979.5596, 799.4845, 655.4384, 637.4410, 493.3903	F14
51	10.06	[M + HCOO]⁻	977.5323	931.5266	$C_{47}H_{80}O_{18}$	-0.4	PPT-Xyl-Glc-Glc	PPT	931.5477, 769.4710, 607.4280, 475.3801	F11
52	10.12	[M + HCOO]⁻	845.4926	799.4844	$C_{42}H_{72}O_{14}$	3.2	OT-Glc-Rha	OT	799.4845, 653.4276, 491.3747, 415.3195	F8
53	10.39	[M + HCOO]⁻	845.4926	799.4844	$C_{42}H_{72}O_{14}$	3.2	OT-Glc-Rha	OT	799.4830, 653.4251,635.4147, 491.3729,415.3196	F8
54	10.49	[M + HCOO]⁻	861.4829	815.4793	$C_{42}H_{72}O_{15}$	-2.8	OT-Glc-Glc	OT	815.4671, 653.4276, 491.3844, 415.3106	F10
55	10.54	[M + HCOO]⁻	989.5347	943.5266	$C_{48}H_{80}O_{18}$	2.6	PPT-Rha-Glc-Glc	PPT	943.5281, 781.4734, 619.4116,475.3705	F10
56	10.76	[M + HCOO]⁻	1025.5518	979.5478	$C_{48}H_{84}O_{20}$	-1.9	(PPT+H_2O)-Glc-Glc-Glc	PPT+H_2O	979.5479,817.4851, 799.4845, 655.4272, 637.4410, 493.3903	F14
57	10.84	[M − H]⁻	973.5006	973.5008	$C_{48}H_{78}O_{20}$	-0.2	B2-GlcA-Glc-Glc	B2	973.5142, 811.4442, 749.4343, 631.3806, 541.3834,473.3542	F11
58	10.88	[M + HCOO]⁻	1049.5519	1003.5478	$C_{50}H_{84}O_{20}$	-1.8	PPT-Glc-Glc-Glc-Ac	PPT	1003.5544,961.5339, 799.4845, 637.4300, 475.3801	F11
59	11.03	[M + HCOO]⁻	1169.5906	1123.5900	$C_{54}H_{92}O_{24}$	-4.2	PPT-Rha-Glc-Glc-Glc	PPT	1123.5908, 943.5146, 781.4734, 619.4008, 475.5515	F14
60	11.07	[M + HCOO]⁻	1005.5260	959.5216	$C_{48}H_{80}O_{19}$	-1.6	B2-Glc-Glc-Glc	B2	959.5173, 797.4731, 779.4482, 635.4030, 473.3542	F13
61	11.14	[M + HCOO]⁻	993.5288	947.5216	$C_{47}H_{80}O_{19}$	-0.6	PPT-Glc-Glc-Xyl	PPT	947.5296, 815.4796, 799.4845, 765.4304, 653.4498,475.3705	F13
62	11.18	[M − H]⁻	887.4979	841.4949	$C_{44}H_{74}O_{15}$	-2.8	(PPT+O)-Glc-Rha-mal	PPT+O	799.4845, 653.4276, 635.4139, 491.3747, 391.2843	F7
63	11.19	[M + HCOO]⁻	1019.5403	973.5372	$C_{49}H_{82}O_{19}$	-2.4	D2-Glc-Rha-Glc	D2	973.5415,811.4940,793.4670,665.4262,647 .4220,503.3729,475.3781	F10

64	11.20	[M − H]⁻	941.4769	941.4746	$C_{47}H_{74}O_{19}$	2.4	(OA+O)-GlcA-Xyl-Glc	OA+O	941.4769, 779.4239, 717.4222, 629.3741, 471.3516	F10
65	11.20	[M − H]⁻	943.4879	943.4903	$C_{47}H_{76}O_{19}$	-2.5	B2-GlcA-Glc-Xyl	B2	943.4879, 781.4368, 719.4354, 631.3806, 587.3945,473.3637	F9
66	11.23	[M + HCOO]⁻	1005.5268	959.5216	$C_{48}H_{80}O_{19}$	-0.8	notoginsenoside G or isomer	B2	959.5173, 797.4731, 635.4030, 473.3542	F13
67	11.26	[M + HCOO]⁻	1169.5959	1123.5900	$C_{54}H_{92}O_{24}$	-0.1	6-O-[β-D-glucopyranosyl-(1→2)-β-D-glucopyranosyl]-20-O-[β-D-glucopyranosyl-(1→4)-β-D-glucopyranosyl]-20(S)-protopanaxatriol	PPT	1123.5907, 961.5367, 799.4810, 781.4718,637.4304,475.3771,391.2861	F14
68	11.28	[M + HCOO]⁻	1019.5403	973.5372	$C_{49}H_{82}O_{19}$	-2.4	D2-Glc-Rha-Glc	D2	973.5415,811.4940,665.4262,503.3729	F11
69	11.31	[M − H]⁻	815.4787	815.4793	$C_{42}H_{72}O_{15}$	-1.5	(PPT+O)-Glc-Glc	PPT+O	653.4276, 491.3747, 391.2756	F7
70	11.35	[M − H]⁻	973.5006	973.5008	$C_{48}H_{78}O_{20}$	-0.2	B2-GlcA-Glc-Glc	B2	973.5142, 811.4442, 761.4246, 749.4343, 631.3915, 541.3936,473.3732	F11
71	11.35	[M + HCOO]⁻	1005.5268	959.5216	$C_{48}H_{80}O_{19}$	-0.8	notoginsenoside G or isomer	B2	959.5173, 797.4731, 779.4482, 635.4030, 473.3542	F13
72	11.35	[M + HCOO]⁻	1005.5268	959.5216	$C_{48}H_{80}O_{19}$	-0.8	B2-Glc-Glc-Glc-Glc	B2	959.5173, 797.4731, 779.4482, 635.4030, 473.3542	F13
73	11.36	[M + HCOO]⁻	1171.6075	1125.6057	$C_{54}H_{94}O_{24}$	-3.2	D1-Glc-Glc-Glc-Glc	D1	1125.6118, 963.5526, 801.4984,783.4890,639.4382,621.4456, 477.3911	F14
74	11.45	[M + HCOO]⁻	1169.5937	1123.5900	$C_{54}H_{92}O_{24}$	-2	6-O-[β-D-glucopyranosyl-(1→2)-β-D-glucopyranosyl]-20-O-[β-D-glucopyranosyl-(1→4)-β-D-glucopyranosyl]-20(S)-protopanaxatriol or isomer	PPT	1123.5908, 961.5474, 799.4845, 637.4300,475.3705	F14
75	11.49	[M + HCOO]⁻	903.4917	857.4899	$C_{44}H_{74}O_{16}$	-4.7	yesanchinoside A	OT	653.4276, 491.3651, 415.3195	F8
76	11.51	[M − H]⁻	943.4879	943.4903	$C_{47}H_{76}O_{19}$	-2.5	B2-GlcA-Glc-Xyl	B2	943.4879,631.3806, 587.3945,473.3637	F9
77	11.52	[M + HCOO]⁻	1023.5373	977.5321	$C_{48}H_{82}O_{20}$	-0.9	C1-Glc-Glc-Glc	C1	977.5378, 815.4547, 653.4164,491.3554,475.3896	F13

78	11.53	$[M-H]^-$	815.4787	815.4793	$C_{42}H_{72}O_{15}$	-1.5	PPT-GlcA-Rha	PPT	815.4796,651.3975, 475.3590	F7
79	11.55	$[M+HCOO]^-$	1273.6453	1227.6374	$C_{58}H_{100}O_{27}$	1.9	D1-Xyl-Glc-Glc-Glc-Xyl	D1	1227.6417, 1095.6045,933.5613,797.4608, 477.407	F14
80	11.61	$[M+HCOO]^-$	863.5040	817.4949	$C_{42}H_{74}O_{15}$	4.2	(PPT+H_2O)-Glc-Glc	PPT+H2O	817.5010,655.4384,493.3903,375.2808	F15
81	11.61	$[M+HCOO]^-$	1167.5791	1121.5744	$C_{54}H_{90}O_{24}$	-1.2	B2-Glc-Glc-Glc-Glc	B2	1121.5864, 959.5173, 797.4608,779.4727,635.4250,473.3542	F14
82	11.66	$[M-H]^-$	809.4302	809.4323	$C_{42}H_{66}O_{15}$	-2.6	(OA+O)-GlcA-Glc	OA+O	809.4302, 647.3802, 585.3853, 471.3516, 439.3293	F8
83	11.67	$[M-H]^-$	811.4442	811.4480	$C_{42}H_{68}O_{15}$	-4.7	(OA+H_2O)-GlcA-Glc	OA+H_2O	811.4442, 649.3929, 587.3945, 473.3637,455.3628	F7
84	11.67	$[M+HCOO]^-$	987.5576	987.5529	$C_{50}H_{84}O_{19}$	-1.1	acetyl-ginsenoside Re	PPT	945.5417,799.4836,783.4878,637.4311,619.4204,475.3780,391.284	F9
85	11.78	$[M+HCOO]^-$	1167.5801	1121.5744	$C_{54}H_{90}O_{24}$	-0.3	B2-Glc-Glc-Glc-Glc	B2	1121.5864, 959.5173, 797.4608,779.4604,635.4139,617.4026,473.3542	F14
86	11.82	$[M+HCOO]^-$	1007.5424	961.5372	$C_{48}H_{82}O_{19}$	-0.9	PPT-Glc-Glc-Glc	PPT	961.5339, 799.4969, 781.4734, 619.4116, 537.3369,475.8467	F13
87	11.85	$[M+HCOO]^-$	863.504	817.4949	$C_{42}H_{74}O_{15}$	4.2	(PPT+H_2O)-Glc-Glc	PPT+H_2O	817.4940, 655.4412,493.3887, 375.2906	F15
88	11.85	$[M-H]^-$	943.5281	943.5266	$C_{48}H_{80}O_{18}$	1.6	B2-Glc-Glc-Rha	B2	943.5281, 797.4855, 763.4382, 635.4360, 473.3447	F10
89	11.93	$[M+HCOO]^-$	1023.5377	977.5321	$C_{48}H_{82}O_{20}$	-0.5	C1-Glc-Glc-Glc	C1	977.5378, 815.4921, 653.4146,637.4300,491.3651,475.3801	F13
90	12.03	$[M-H]^-$	941.4769	941.4746	$C_{47}H_{74}O_{19}$	2.4	(OA-2H)-GlcA-Xyl-Glc	OA-2H	941.4769, 779.4239, 761.4125, 629.3741, 523.3785, 453.3336, 409.3517	F8
91	12.08	$[M+HCOO]^-$	887.4979	841.4949	$C_{44}H_{74}O_{15}$	-2.8	vinaginsenoside R1	OT	841.4848, 799.4722, 653.4387, 491.3747,415.0081	F6
92	12.12	$[M+HCOO]^-$	863.504	817.4949	$C_{42}H_{74}O_{15}$	4.2	(PPT+H_2O)-Glc-Glc	PPT+H_2O	817.5010,655.4384,493.3903,375.2893	F14
93	12.14	$[M+HCOO]^-$	861.4848	815.4793	$C_{42}H_{72}O_{15}$	-0.6	OT-Glc-Glc	OT	815.4796, 653.4276, 491.3747, 415.3195	F9
94	12.26	$[M-H]^-$	941.4769	941.4746	$C_{47}H_{74}O_{19}$	2.4	(OA+O)-GlcA-Xyl-Glc	OA+O	941.4769, 779.4239, 717.4222, 629.3741, 585.3748, 471.3516, 453.3336, 409.3429	F8
95	12.36	$[M+HCOO]^-$	1007.5425	961.5372	$C_{48}H_{82}O_{19}$	-0.2	ginsenoside Re1	PPT	961.5339, 799.4722, 637.4300, 475.3801	F13
96	12.55	$[M+HCOO]^-$	1007.5415	961.5372	$C_{48}H_{82}O_{19}$	-1.7	notoginsenoside R6	PPT	961.5339, 799.4722, 781.4734, 637.4410, 475.3610	F13
97	12.67	$[M-H]^-$	1249.5881	1249.5853	$C_{59}H_{94}O_{28}$	2.2	OA-Glc-Xyl-Glc-Glc-GlcA	OA	1249.5881, 947.4624, 731.4323, 569.3837, 523.3785, 455.3534	F14

98	12.68	[M + HCOO]⁻	887.4987	841.4949	$C_{44}H_{74}O_{15}$	-2.6	OT-Glc-Rha-Ac	OT	841.4975, 799.4845, 653.4276, 491.3651, 415.3195	F6
99	12.73	[M − H]⁻	809.4302	833.4323	$C_{44}H_{66}O_{15}$	-2.6	(OA-2H)-GlcA-Glc	OA-2H	809.4302, 629.3632, 585.3853,567.3744, 453.3429, 409.3429	F6
100	12.99	[M − H]⁻	1087.5328	1087.5325	$C_{53}H_{84}O_{23}$	-0.3	stipuleanoside R2 or isomer	OA	1087.5391, 763.4261, 747.4272, 613.3835, 455.3441	F12
101	13.19	[M − H]⁻	971.4852	971.4852	$C_{48}H_{76}O_{20}$	0	(OA+O)-Glc-Glc-GlcA	OA+O	971.4852, 809.4302, 629.3522, 585.3853,471.3422	F15
102	13.21	[M + HCOO]⁻	831.4727	785.4687	$C_{41}H_{70}O_{14}$	-2.5	majonoside R2 or isomer	OT	785.4704, 653.4276, 491.3747, 415.3195	F7
103	13.21	[M − H]⁻	1087.5323	1087.5325	$C_{53}H_{84}O_{23}$	-0.7	OA-GlcA-Xyl-Glc-Glc	OA	1087.5391, 793.4334, 613.3835, 455.3628	F12
104	13.22	[M − H]⁻	971.4852	971.4852	$C_{48}H_{76}O_{20}$	0	(OA+O)-GlcA-Glc-Glc	OA+O	971.4852, 809.4302, 747.4272, 629.3632, 585.3748, 471.3422	F10
105	13.33	[M − H]⁻	1117.5415	1117.5431	$C_{54}H_{86}O_{24}$	-1.9	(OA-2H)-Rha-Glc-Glc-GlcA	OA-2H	1117.5389, 943.5281, 781.4734, 619.4225,567.3616,453.3131	F11
106	13.33	[M − H]⁻	1117.5389	1117.5431	$C_{54}H_{86}O_{24}$	-3	D2-Xyl-Rha-GlcA-Glc	D2	1117.5389, 955.4866,761.4005, 625.3381, 553.3573, 503.3827	F11
107	13.35	[M − H]⁻	971.4811	971.4852	$C_{48}H_{76}O_{20}$	-4.2	(OA+O)-Glc-Glc-GlcA	OA+O	971.4811,809.4269,629.3605,585.3723,539 .3611,471.3307,439.3184	F4
108	13.36	[M + HCOO]⁻	845.4907	799.4844	$C_{42}H_{72}O_{14}$	0.4	ginsenoside Re2	PPT	799.4845, 637.4300, 475.3801, 391.2843	F8
109	13.40	[M − H]⁻	971.4852	971.4852	$C_{48}H_{76}O_{20}$	0	(OA+O)-GlcA-Glc-Glc	OA+O	971.4852, 809.4302, 629.3632, 585.3748, 471.3422	F2
110	13.40	[M − H]⁻	1117.5389	1117.5431	$C_{54}H_{86}O_{24}$	-3.8	OA-GlcA-Glc-GlcA-Rha	OA	1117.5389, 793.4334, 701.4180, 613.3619, 569.3837, 455.3534	F14
111	13.44	[M − H]⁻	971.4852	971.4852	$C_{48}H_{76}O_{20}$	0	(OA+O)-Glc-Glc-GlcA	OA+O	971.4852, 831.4190, 809.4302, 747.4272, 629.3632, 585.3748, 471.3422	F12
112	13.48	[M − H]⁻	1087.5321	1087.5325	$C_{53}H_{84}O_{23}$	-0.9	OA-GlcA-Xyl-Glc-Glc	OA	1087.5391, 925.4799, 763.4261, 613.3727, 455.3441	F8
113	13.58	[M − H]⁻	971.4852	971.4852	$C_{48}H_{76}O_{20}$	0	(OA+O)-Glc-Glc-GlcA	OA+O	971.4852, 809.4302, 747.4272, 629.3632, 585.3748, 471.3422	F12
114*	13.59	[M + HCOO]⁻	845.4879	799.4844	$C_{42}H_{72}O_{14}$	-3	pseudoginsenoside F11	OT	799.4845, 653.4276, 491.3747, 415.3195	F7
115	13.61	[M − H]⁻	941.4769	941.4746	$C_{47}H_{74}O_{19}$	2.4	(OA+O)-GlcA-Xyl-Glc	OA+O	941.4769, 779.4239, 717.4222, 629.3741, 585.3748, 471.3516	F10
116	13.62	[M − H]⁻	1087.5319	1087.5325	$C_{53}H_{84}O_{23}$	-1	OA-GlcA-Xyl-Glc-Glc	OA	1087.5391, 763.4261,613.3727,455.3534	F8
117	13.69	[M + HCOO]⁻	845.4899	799.4844	$C_{42}H_{72}O_{14}$	-0.7	ginsenoside Rf	PPT	799.4845, 637.4410, 475.3801, 391.2843	F13
118	13.79	[M − H]⁻	1087.5304	1087.5325	$C_{53}H_{84}O_{23}$	-2.5	OA-GlcA-Xyl-Glc-Glc	OA	1087.5391, 763.4261,613.3727,455.3534	F8
119	13.83	[M + HCOO]⁻	863.5040	817.4949	$C_{42}H_{74}O_{15}$	4.2	(PPT+H_2O)-Glc-Glc	PPT+H_2O	817.5010,655.4384,493.3903,475.3801	F14

120	13.85	$[M-H]^-$	1087.5320	1087.5325	$C_{53}H_{84}O_{23}$	-1	OA-GlcA-Xyl-Glc-Glc	OA	1087.5391, 785.4092, 763.4261, 613.3727, 569.3837, 455.3441	F12
121	13.97	$[M+HCOO]^-$	1285.6404	1239.6374	$C_{59}H_{100}O_{27}$	-2.3	OA-GlcA-Glc-Glc-Glc-Xyl	OA	1239.6359, 1107.5906, 945.5546, 783.4767, 621.4674, 569.3837, 455.3441	F14
122	13.99	$[M-H]^-$	1057.5214	1057.5219	$C_{52}H_{82}O_{22}$	-0.5	OA-GlcA-Xyl-Glc-Xyl	OA	1057.5214, 763.4261, 613.3727, 569.3837, 523.3785,455.3628	F9
123	14.03	$[M-H]^-$	1087.5315	1087.5325	$C_{53}H_{84}O_{23}$	-1.5	OA-GlcA-Xyl-Glc-Glc	OA	1087.5391, 785.4092, 763.4261, 613.3727, 569.3837, 455.3441	F12
124	14.05	$[M-H]^-$	955.4903	955.4903	$C_{48}H_{76}O_{19}$	0.2	OA-GlcA-Glc-Glc	OA	955.4905, 653.3717, 631.3806, 569.3837, 455.3534	F9
125	14.18	$[M-H]^-$	1087.5295	1087.5325	$C_{53}H_{84}O_{23}$	-3.3	OA-GlcA-Xyl-Glc-Glc	OA	1087.5391, 763.4261, 713.4323, 569.3837	F12
126	14.20	$[M+HCOO]^-$	815.4780	769.4738	$C_{41}H_{70}O_{13}$	-2.2	notoginsenoside R2	PPT	769.4710, 637.4300, 475.3801, 391.2843	F7
127	14.20	$[M-H]^-$	1087.5343	1087.5325	$C_{53}H_{84}O_{23}$	1.1	OA-GlcA-Xyl-Glc-Glc	OA	1087.5391, 925.4799, 763.4261	F8
128	14.38	$[M-H]^-$	1087.5338	1087.5325	$C_{53}H_{84}O_{23}$	0.7	OA-GlcA-Xyl-Glc-Glc	OA	1087.5391, 763.4261, 729.4259, 569.3837	F12
129	14.41	$[M-H]^-$	941.4769	941.4746	$C_{47}H_{74}O_{19}$	2.4	(OA+O)-GlcA-Xyl-Glc	OA+O	941.4769, 779.4239, 717.4222, 631.3806, 585.3748, 569.3733, 471.3422	F8
130*	14.57	$[M-H]^-$	955.4903	955.4903	$C_{48}H_{76}O_{19}$	0.2	hemsgiganoside B	OA	955.4891, 631.3838, 455.3519	F9
131	14.66	$[M-H]^-$	1087.534	1087.5325	$C_{53}H_{84}O_{23}$	0.9	stipuleanoside R2 or isomer	OA	1087.5391, 785.4092, 763.4261, 613.3727, 569.3837, 497.3557, 455.3441	F11
132	14.68	$[M-H]^-$	955.4905	955.4903	$C_{48}H_{76}O_{19}$	0.2	OA-GlcA-Glc-Glc	OA	955.4905,631.3915,455.3441	F4
133	14.73	$[M-H]^-$	955.4905	955.4903	$C_{48}H_{76}O_{19}$	0.2	OA-GlcA-Glc-Glc	OA	955.4905, 793.4334, 761.4125, 731.4323, 569.3837, 523.3785,455.3534	F9
134	14.85	$[M-H]^-$	941.4769	941.4746	$C_{47}H_{74}O_{19}$	2.4	(OA+O)-GlcA-Xyl-Glc	OA+O	941.4769, 779.4239, 717.4222, 631.3806, 585.3748, 569.3733, 471.3422	F11
135	14.93	$[M-H]^-$	955.4905	955.4903	$C_{48}H_{76}O_{19}$	0.2	OA-GlcA-Glc-Glc	OA	955.4905, 631.3806, 569.3837, 455.3834	F9
136	14.97	$[M-H]^-$	1087.5316	1087.5325	$C_{53}H_{84}O_{23}$	-1.3	stipuleanoside R2 or isomer	OA	1087.5391, 925.4932,731.4441, 613.3727, 569.3837,523.3785,411.3687	F11
137	14.98	$[M+HCOO]^-$	1009.5534	963.5529	$C_{48}H_{84}O_{19}$	-4.9	D1-Glc-Glc-Glc	D1	963.5526, 801.4984, 639.4492, 477.3911	F15
138	15.07	$[M+HCOO]^-$	1285.6423	1239.6374	$C_{59}H_{100}O_{27}$	-0.5	(OA+O)-GlcA-Xyl-Glc-Glc-Xyl	OA+O	1239.6359, 1107.5906, 941.4769, 779.4239, 629.3741, 585.3959, 471.3516, 455.3628	F14
139	15.29	$[M-H]^-$	941.4769	941.4746	$C_{47}H_{74}O_{19}$	2.4	(OA+O)-GlcA-Xyl-Glc	OA+O	941.4769, 779.4239, 717.4222, 629.3632, 585.3748, 471.3422, 455.3534	F8
140	15.41	$[M-H]^-$	1249.5881	1249.5853	$C_{59}H_{94}O_{28}$	2.2	OA-Glc-Xyl-Glc-Glc-GlcA	OA	1249.5881, 955.4905, 731.4441, 653.3717, 631.3806, 569.3837, 523.3785, 455.3534	F14
141	15.43	$[M+HCOO]^-$	829.4938	783.4895	$C_{42}H_{72}O_{13}$	-2.1	ginsenoside Rg2	PPT	783.4890, 637.4300, 475.3801, 391.2843	F6
142*	15.52	$[M+HCOO]^-$	683.4352	637.4316	$C_{36}H_{62}O_9$	-3.5	ginsenoside Rh1	PPT	637.4189, 585.3959, 475.3801, 391.2843	F5

143	15.52	[M + HCOO]⁻	829.4937	783.4895	$C_{42}H_{72}O_{13}$	-1.8	PPT-Glc-Rha	PPT	783.4890,585.3853,475.3801	F18
144	15.60	[M − H]⁻	955.4866	955.4903	$C_{48}H_{76}O_{19}$	3.9	(OA+O)-GlcA-Xyl-Glc-methyl ester	OA+O	955.4866,941.4730,631.3779,471.3497	F4
145	15.61	[M − H]⁻	1057.5214	1056.5141	$C_{52}H_{82}O_{22}$	-0.5	OA-GlcA-Xyl-Glc-Xyl	OA	925.4799, 763.4261, 613.3727, 455.3534	F8
146	15.64	[M − H]⁻	1107.6051	1107.5587	$C_{53}H_{88}O_{24}$	0.2	OA-Xyl-Rha-GlcA-Glc	OA	1107.6051, 955.4636, 731.4441, 569.3733, 523.3785, 455.3628	F1
147	15.66	[M − H]⁻	1087.5311	1087.5325	$C_{53}H_{84}O_{23}$	-1.8	OA-GlcA-Xyl-Glc-Glc	OA	1087.5247, 731.4323, 613.3727, 569.3837, 523.3785, 455.3534	F7
148	15.70	[M + HCOO]⁻	1255.6312	1209.6268	$C_{58}H_{98}O_{26}$	-0.9	OA-GlcA-Xyl-Glc-Xyl-Xyl	OA	1209.6230, 1077.5820, 783.5012, 569.3837, 455.3628	F14
149	15.78	[M + HCOO]⁻	829.4943	783.4895	$C_{42}H_{72}O_{13}$	-1.4	ginsenoside Rg2 or isomer	PPT	783.4890,637.4300,475.3801,391.2843	F11
150	15.88	[M + HCOO]⁻	1153.6058	1153.6006	$C_{55}H_{94}O_{25}$	4.5	PPD-Glc-Glc-Glc-Glc	PPD	1107.6196,945.5546,783.4890,621.7069,459.7811	F4
151	15.99	[M + HCOO]⁻	1009.5534	963.5529	$C_{48}H_{84}O_{19}$	-4.9	D1-Glc-Glc-Glc	D1	963.5526, 801.4984, 639.4492, 477.3911	F15
152	16.00	[M + HCOO]⁻	1249.5881	1249.5853	$C_{59}H_{94}O_{28}$	2.2	PPT-Glc-Rha-GlcA-Glc-Xyl	PPT	1249.5881,1153.5536,955.5040,637.4300,475.3801,391.2929	F14
153	16.02	[M − H]⁻	1087.5311	1087.5325	$C_{53}H_{84}O_{23}$	6.1	OA-GlcA-Glc-Xyl-Rha	OA	1087.5247, 947.4785, 881.4459, 809.4427, 647.3802, 613.3727, 569.3727, 455.3534	F7
154	16.11	[M + HCOO]⁻	829.4947	783.4895	$C_{42}H_{72}O_{13}$	-1	ginsenoside Rg2 or isomer	PPT	783.4890, 637.4300, 475.3801, 391.2843	F10
155	16.12	[M − H]⁻	923.4617	923.4640	$C_{47}H_{72}O_{18}$	-2.5	(OA-2H)-GlcA-Xyl-Glc	OA-2H	923.4617, 761.4125, 699.4070, 611.3520, 567.3640, 453.3336	F9
156	16.13	[M − H]⁻	809.4302	809.4323	$C_{42}H_{66}O_{15}$	-2.6	(OA+O)-Glc-GlcA	OA+O	809.4302, 647.3802, 629.3741, 585.3853,471.3516, 453.3429	F6
157	16.19	[M − H]⁻	809.4302	809.4323	$C_{42}H_{66}O_{15}$	-2.6	(OA+O)-Glc-GlcA	OA+O	809.4178, 647.3802, 471.3422	F16
158	16.26	[M − H]⁻	1087.5295	1087.5325	$C_{53}H_{84}O_{23}$	-3.3	stipuleanoside R2 or isomer	OA	1087.5247, 731.4323, 569.3837, 523.3785,455.3534	F11
159	16.29	[M − H]⁻	1057.5214	1057.5219	$C_{52}H_{82}O_{22}$	-2.5	(OA-2H)-Glc-GlcA-Xyl-Xyl	OA-2H	1057.4617, 923.4617, 791.4297, 613.3727, 567.3640,453.3336	F7
160	16.31	[M − H]⁻	1057.5214	1056.5141	$C_{52}H_{81}O_{22}$	-0.5	OA-GlcA-Xyl-Glc-Xyl	OA	1057.5214, 701.4296, 523.3785,497.3557,455.3534	F9
161*	16.45	[M + HCOO]⁻	683.4349	637.4316	$C_{36}H_{62}O_{9}$	-3.1	20(R)-ginsenoside Rh1	PPT	637.4189,475.7800	F5
162	16.56	[M − H]⁻	1087.5316	1087.5325	$C_{53}H_{84}O_{23}$	-1.4	OA-GlcA-Xyl-Glc-Glc	OA	1087.5247, 731.4323, 569.3837, 523.3785	F12
163*	16.71	[M + HCOO]⁻	1153.6058	1153.6006	$C_{55}H_{94}O_{25}$	4.5	ginsenoside Rb1	PPD	1107.5906, 945.5412, 783.4890, 621.4348,459.3786	F14

164	16.73	[M − H]⁻	1057.5214	1056.5141	$C_{52}H_{82}O_{22}$	-0.5	OA-GlcA-Xyl-Glc-Xyl	OA	1057.5214, 785.4214, 613.3727, 569.3837,497.3655,455.3441	F10
165	16.87	[M − H]⁻	925.4811	925.4797	$C_{47}H_{74}O_{18}$	0.9	stipuleanoside R1	OA	925.4799, 731.4441, 613.3727, 569.3837,455.3534	F7
166	17.04	[M − H]⁻	1087.5323	1087.5325	$C_{53}H_{84}O_{23}$	-0.7	OA-GlcA-Xyl-2Glc	OA	1087.5247, 731.4323, 551.3765,455.3534	F10
167*	17.08	[M − H]⁻	955.4905	955.4903	$C_{48}H_{76}O_{19}$	0.2	Chikusetsusaponin Ⅴ	OA	955.483, 793.4343, 731.4336,569.3819,523.3755,455.3499	F9
168	17.27	[M − H]⁻	1057.5214	1057.5219	$C_{52}H_{82}O_{22}$	-0.5	OA-GlcA-Xyl-Glc-Xyl	OA	1057.5214, 701.4296, 523.3785,455.3534	F9
169	17.42	[M − H]⁻	1087.533	1087.5325	$C_{53}H_{84}O_{23}$	0	OA-GlcA-Xyl-Glc-Glc	OA	1087.5391, 947.4624, 613.3727, 569.3837,455.3534	F7
170	17.53	[M − H]⁻	1087.5314	1087.5325	$C_{53}H_{84}O_{23}$	-1.5	stipuleanoside R2 or isomer	OA	1087.5247, 731.4323, 551.3765,455.3534	F11
171	17.87	[M − H]⁻	1087.5322	1087.5325	$C_{53}H_{84}O_{23}$	-0.8	OA-GlcA-Xyl-Glc-Glc	OA	1087.5247, 925.4799, 785.4092, 613.3727, 569.3837,455.3534	F7
172	17.89	[M − H]⁻	955.4909	955.4903	$C_{48}H_{76}O_{19}$	0.1	OA-Glc-Glc-GlcA	OA	955.4905, 793.4334, 569.3837,523.3785,497.3655,455.3534	F9
173	17.93	[M − H]⁻	793.435	793.4374	$C_{42}H_{66}O_{14}$	-3.7	OA-3-GlcA-28-Glc	OA	793.4334, 631.3806, 569.3942, 455.3534	F16
174	17.97	[M + HCOO]⁻	847.5037	801.5000	$C_{42}H_{74}O_{14}$	-2.8	D1-Glc-Glc	D1	801.4984, 639.4492, 477.3911, 401.3511	F15
175	17.98	[M + HCOO]⁻	1123.5905	1077.5845	$C_{53}H_{90}O_{22}$	-0.1	ginsenoside Rc	PPD	1077.5820, 945.5412, 815.4172, 731.4559, 613.3727,459.3786	F13
176	18.04	[M − H]⁻	1057.5214	1057.5219	$C_{52}H_{82}O_{22}$	-0.5	OA-GlcA-Xyl-Glc-Xyl	OA	925.4799, 763.4261, 613.3727, 569.3837,455.3534	F7
177	18.19	[M − H]⁻	925.4752	925.4797	$C_{47}H_{74}O_{18}$	-5.5	stipuleanoside R1	OA	925.4799, 763.4261, 613.3727,569.3837,523.3785,455.3441	F8
178	18.19	[M + HCOO]⁻	1195.6088	1149.6057	$C_{56}H_{94}O_{24}$	-2.4	quinquenoside R1	PPD	1149.6047, 1107.5934,945.5414,927.5230,783.4874,765.4755,621.4351,603.4391,459.3824	F13
179	18.31	[M + HCOO]⁻	1195.615	1149.6057	$C_{56}H_{94}O_{24}$	3.2	acetyl-ginsenoside Rb1 or isomer	PPD	1149.6047, 1107.5934,945.5414,927.5230,783.4874,765.4755,621.4351,603.4391,459.3824	F13
180	18.56	[M + HCOO]⁻	1255.6313	1209.6268	$C_{58}H_{98}O_{26}$	-1.2	ginsenoside Ra1	PPD	1209.6230, 1077.5820, 945.5278, 783.5012,765.4788, 701.4180,621.4456,459.3786	F14
181	18.63	[M − H]⁻	923.4617	923.4640	$C_{47}H_{72}O_{18}$	-2.5	OA-GlcA-Glc-Xyl	OA	923.4617, 793.4334, 631.3806, 613.3727, 569.3837, 523.3785,455.3441	F7
182	18.63	[M − H]⁻	1087.534	1087.5325	$C_{53}H_{84}O_{23}$	0.9	OA-GlcA-Xyl-Glc-Glc	OA	1087.5247, 925.4799, 523.3785	F11

183	18.71	[M − H]⁻	1071.5411	1071.5376	$C_{53}H_{84}O_{22}$	3.3	OA-Rha-Xyl-Xyl-GlcA-ethyl ester	OA	1071.5411, 1043.5515, 849.4971, 717.4222, 569.3837, 523.3785, 455.3534	F9
184	18.73	[M + HCOO]⁻	847.5037	801.5000	$C_{42}H_{74}O_{14}$	-2.8	D1-Glc-Glc	D1	801.4984, 639.4492, 477.3911	F15
185	19.01	[M + HCOO]⁻	847.5037	801.5000	$C_{42}H_{74}O_{14}$	-2.8	D1-Glc-Glc	D1	801.4984, 639.4492, 477.3911	F14
186	19.04	[M − H]⁻	955.4957	955.4903	$C_{48}H_{76}O_{19}$	5.1	OA-GlcA-Glc-Glc	OA	955.4905, 793.4334, 731.4441, 569.3837, 523.3785,455.3534	F10
187	19.06	[M − H]⁻	925.4810	925.4797	$C_{47}H_{74}O_{18}$	0.8	stipuleanoside R1	OA	925.4799, 763.4261, 613.3727, 569.3837,523.3785,455.3534	F9
188	19.10	[M − H]⁻	925.4808	925.4797	$C_{47}H_{74}O_{18}$	0.6	pseudoginsenoside Rt1 or isomer	OA	925.4799, 763.4261, 701.4180, 613.3835, 569.3837, 455.3534	F1
189	19.12	[M + HCOO]⁻	887.4998	841.4949	$C_{44}H_{74}O_{15}$	-1.4	OT-Glc-Rha-Ac	OT	841.4975,799.4845,653.4276,491.3651,415 .3284	F6
190	19.32	[M − H]⁻	925.4803	925.4797	$C_{47}H_{74}O_{18}$	0	pseudoginsenoside Rt1 or isomer	OA	925.4799, 763.4261, 701.4180, 613.3835,569.3837,523.3785,455.3534	F15
191	19.32	[M − H]⁻	1057.5214	1056.5141	$C_{52}H_{82}O_{22}$	-0.5	OA-Xyl-Xyl-GlcA-Glc	OA	1057.5214, 895.4645,763.4261,745.4229,701.4296,683 .4235,613.3727,595.3698,551.3765,455.35 34	F10
192*	19.43	[M − H]⁻	925.4788	925.4797	$C_{47}H_{74}O_{18}$	-1.5	chikusetsusaponin Ⅳ	OA	925.4799, 763.4261, 701.4296, 613.3727, 569.3837,455.3441	F7
193	19.64	[M − H]⁻	925.4797	925.4797	$C_{47}H_{74}O_{18}$	-0.6	OA-GlcA-Xyl-Glc	OA	925.4799, 613.3727, 569.3837	F15
194	19.69	[M − H]⁻	925.4775	925.4797	$C_{47}H_{74}O_{18}$	-3	OA-GlcA-Xyl-Glc	OA	925.4799, 613.3727, 569.3837,455.3534,437.3362	F6
195	19.71	[M − H]⁻	925.4769	925.4797	$C_{47}H_{74}O_{18}$	-3.6	OA-GlcA-Xyl-Glc	OA	925.4666, 763.4261, 701.4180, 613.3727,569.3837,523.3785,455.3441	F8
196	19.86	[M + HCOO]⁻	847.5037	801.5000	$C_{42}H_{74}O_{14}$	-2.8	D1-Glc-Glc	D1	801.4984, 639.4492, 477.3911	F14
197	19.86	[M − H]⁻	925.4804	925.4797	$C_{47}H_{74}O_{18}$	0.1	pseudoginsenoside Rt1 or isomer	OA	925.4799, 763.4261, 701.4296, 613.3727, 569.3837, 455.3534	F2
198	19.97	[M − H]⁻	925.477	925.4797	$C_{47}H_{74}O_{18}$	-3.5	OA-GlcA-Xyl-Glc	OA	925.4666, 763.4141,613.3619,569.3733,455.3441	F7
199	20.21	[M − H]⁻	925.4793	925.4797	$C_{47}H_{74}O_{18}$	-1	OA-GlcA-Xyl-Glc	OA	925.4799, 613.3727, 569.3837	F2
200*	20.34	[M − H]⁻	925.4797	925.4797	$C_{47}H_{74}O_{18}$	-0.5	chikusetsusaponin Ib	OA	925.4799, 613.3727, 569.3837	F11
201	21.04	[M − H]⁻	939.4944	939.4953	$C_{48}H_{76}O_{18}$	-1.6	pseudoginsenoside Rt1 methyl ester	OA	939.4963,777.4427,613.3745,569.3848,523 .3790,455.3525	F8
202	21.29	[M − H]⁻	793.4374	793.4374	$C_{42}H_{66}O_{14}$	-0.7	OA-3-GlcA-28-Glc	OA	793.4334, 631.3806, 569.3837, 455.3534	F1

203	21.32	[M + HCOO]⁻	1195.6105	1149.6057	$C_{56}H_{94}O_{24}$	-1	acetyl-ginsenoside Rb1 or isomer	PPD	1149.6047, 1107.5934,945.5414,927.5230,783.4874,765.4755,621.4351,459.3860	F13
204	21.38	[M − H]⁻	793.4362	793.4374	$C_{42}H_{66}O_{14}$	-2.3	OA-3-GlcA-28-Glc	OA	793.4334, 631.3806,613.3727,455.3534	F16
205	21.54	[M − H]⁻	793.4373	793.4374	$C_{42}H_{66}O_{14}$	-0.8	OA-3-GlcA-28-Glc	OA	793.4334, 631.3806, 569.3837, 455.3534	F15
206	21.60	[M − H]⁻	793.4334	793.4374	$C_{42}H_{66}O_{14}$	-5	OA-3-GlcA-28-Glc	OA	793.4334, 631.3806, 569.3837, 455.3534	F4
207	21.69	[M − H]⁻	793.4366	793.4374	$C_{42}H_{66}O_{14}$	-1.7	OA-3-GlcA-28-Glc	OA	793.4334, 631.3806, 569.3837, 455.3534	F16
208*	21.72	[M − H]⁻	793.4367	793.4374	$C_{42}H_{66}O_{14}$	-1.7	Chikusetsusaponin IVa	OA	793.4334, 631.3806, 569.3837,455.3534	F6
209	21.81	[M + HCOO]⁻	975.5539	929.5110	$C_{47}H_{78}O_{18}$	0.5	OA-Xyl-Glc-Glc	OA	929.5495, 701.4296, 605.4493, 455.3628	F9
210	21.92	[M + HCOO]⁻	1255.6308	1209.6268	$C_{58}H_{98}O_{26}$	-1.6	PPT-Glc-Glc-Rha-Xyl-Xyl	PPT	1209.6230, 1077.5820, 945.5278,799.4845,637.4300, 475.3801	F14
211	21.98	[M − H]⁻	925.4818	925.4797	$C_{47}H_{74}O_{18}$	1.6	pjs-2	OA	925.4799, 763.4261, 701.4296, 613.3835, 569.3837, 455.3534	F8
212*	22.15	[M − H]⁻	793.4334	793.4374	$C_{42}H_{66}O_{14}$	-5	Cynarasaponin C	UA	793.4334, 631.3806, 569.3837, 497.3655, 455.3534	F2
213	22.34	[M − H]⁻	793.4369	793.4374	$C_{42}H_{66}O_{14}$	-1.3	OA-3-GlcA-28-Glc	OA	793.4334, 631.3806, 569.3837, 455.3534	F11
214	22.87	[M − H]⁻	793.4373	793.4374	$C_{42}H_{66}O_{14}$	-0.8	OA-3-GlcA-28-Glc	OA	793.4334, 631.3806, 569.3837, 455.3534	F14
215*	23.36	[M + HCOO]⁻	991.5481	945.5423	$C_{48}H_{82}O_{18}$	-0.3	ginsenoside Rd	PPD	945.5412, 783.4890, 621.4456, 459.3879	F10
216	23.58	[M − H]⁻	1297.6431	1209.6268	$C_{58}H_{98}O_{26}$	0.2	PPD-Glc-Glc-Glc-Xyl-Xyl-mal	PPD	1209.6332,1077.5776,945.5641,783.4857,765.4634,621.4434,459.3766	F14
217	23.72	[M + HCOO]⁻	985.5002	939.4953	$C_{48}H_{76}O_{18}$	-0.4	OA-GlcA-Glc-Xyl	OA	939.4949, 789.4407, 771.4468, 609.3777, 455.3534	F9
218	24.05	[M + HCOO]⁻	991.5479	945.5423	$C_{48}H_{82}O_{18}$	-0.5	ginsenoside Rd6	PPD	945.5412, 783.4890, 765.4788,621.4456, 603.4202, 459.3879	F11
219	24.76	[M + HCOO]⁻	985.5021	939.4953	$C_{48}H_{76}O_{18}$	0.8	pseudoginsenoside Rt1 methyl ester	OA	939.4944,789.4414,609.3785,455.3515	F11
220	25.18	[M + HCOO]⁻	985.5021	939.4953	$C_{48}H_{76}O_{18}$	0.8	pseudoginsenoside Rt1 methyl ester	OA	939.4944,789.4414,609.3785,455.3515	F12
221	25.29	[M + HCOO]⁻	843.4750	797.4687	$C_{42}H_{70}O_{14}$	0.3	B2-Glc-Glc	B2	797.4731, 635.4139, 473.3637	F14
222	25.29	[M + HCOO]⁻	991.5488	945.5423	$C_{48}H_{82}O_{18}$	0.5	gypenoside XVII or isomer	PPD	945.5412, 783.4890, 621.4456, 459.3879	F10
223	25.33	[M − H]⁻	923.4617	923.4640	$C_{47}H_{72}O_{18}$	-2.5	(OA-2H)-GlcA-Xyl-Glc	OA-2H	761.4125, 611.3628, 567.3744, 453.3336	F8
224	25.38	[M + HCOO]⁻	991.5467	945.5423	$C_{48}H_{82}O_{18}$	-1.7	gypenoside XVII or isomer	PPD	945.5412, 783.4890, 621.4456, 459.3879	F15

225	25.38	[M + HCOO]⁻	1195.6105	1149.6057	$C_{56}H_{94}O_{24}$	-1	acetyl-ginsenoside B1	PPD	1149.6047,1107.5934,945.5414,783.4874,765.4755,621.4351,459.3860	F13
226	25.50	[M + HCOO]⁻	1117.5536	1117.5067	$C_{53}H_{82}O_{25}$	2.8	achyranthoside D	OA	1041.5070,955.5040,793.4334,731.4441,613.3727, 455.3534	F9
227	25.52	[M + HCOO]⁻	889.5155	843.5106	$C_{44}H_{76}O_{15}$	-0.7	D1-Glc-Glc-Ac	D1	843.5128,801.4984,639.4382, 477.4007	F14
228	25.62	[M + HCOO]⁻	1033.5597	987.5529	$C_{50}H_{84}O_{19}$	1.4	PPD-Glc-Glc-Ac	PPD	987.5576,945.5412,783.4890,621.4348,459.3879	F8
229	25.69	[M + HCOO]⁻	1123.5908	1077.5845	$C_{53}H_{90}O_{22}$	0.7	OA-GlcA-Xyl-Glc-Xyl	OA	1077.5820, 945.5412, 759.4256, 613.3727, 455.3534	F13
230	25.72	[M + HCOO]⁻	889.5155	843.5106	$C_{44}H_{76}O_{15}$	-0.7	D1-Glc-Glc-Ac	D1	843.5255,801.4984,639.4382, 477.4007	F14
231	25.74	[M − H]⁻	1107.5906	1107.5951	$C_{54}H_{92}O_{23}$	0.2	ginsenoside B1	PPD	1107.5906, 945.5546, 783.4767,621.4239,459.3879	F13
232	25.75	[M + HCOO]⁻	1221.6252	1175.6213	$C_{58}H_{96}O_{24}$	-1.7	ginsenoside Ra6	PPD	1175.6168,1107.5906,1089.5850, 945.5546, 783.4767,621.4239,459.3879	F13
233	25.78	[M + HCOO]⁻	991.5485	945.5423	$C_{48}H_{82}O_{18}$	0.2	gypenoside XVII or isomer	PPD	945.5412, 783.4890, 621.4456, 459.3879	F12
234	25.81	[M − H]⁻	939.4929	939.4953	$C_{48}H_{76}O_{18}$	-3.2	pseudoginsenoside Rt1 methyl ester	OA	939.4963,807.4527,627.3892,609.3783,569.3848,523.3790,455.3525	F9
235	25.83	[M + HCOO]⁻	985.501	939.4953	$C_{48}H_{76}O_{18}$	-0.4	pseudoginsenoside Rt1 methyl ester or isomer	OA	807.4561, 627.3928, 537.3571, 455.3534	F7
236	25.84	[M + HCOO]⁻	991.5413	945.5423	$C_{48}H_{82}O_{18}$	-6.6	PPD-Glc-Glc-Glc	PPD	945.5412, 783.4890, 621.4456,459.3879	F15
237	25.86	[M + HCOO]⁻	853.4603	807.4531	$C_{43}H_{68}O_{14}$	1.4	chikusetsusaponin IVa methyl ester or isomer	OA	807.4561, 609.3777, 537.3571, 455.3534	F8
238	25.89	[M + HCOO]⁻	853.4583	807.4531	$C_{43}H_{68}O_{14}$	-0.9	chikusetsusaponin IVa methyl ester or isomer	OA	807.4436,627.3928,609.3777,537.3571,455.3534	F19
239	25.92	[M + HCOO]⁻	853.4565	807.4531	$C_{43}H_{68}O_{14}$	1.4	spinasaponinA methyl ester or isomer	OA	807.4527,645.4013,627.3901,609.3751,455.3515	F4
240	25.94	[M + HCOO]⁻	1093.5796	1047.574	$C_{52}H_{88}O_{21}$	1.7	OA-Rha-Rha-Xyl-Xyl	OA	1047.5758, 915.5298, 621.4456, 569.3942, 455.3441	F13
241	25.97	[M + HCOO]⁻	843.4735	797.4687	$C_{42}H_{70}O_{14}$	-1.5	B2-Glc-Glc	B2	797.4731, 635.4139, 473.3637	F14
242	26.01	[M + HCOO]⁻	975.5539	929.5474	$C_{48}H_{82}O_{17}$	0.5	OA-Xyl-Glc-Glc	OA	929.5495, 767.4977, 605.4493, 455.3534	F9
243	26.02	[M + HCOO]⁻	827.4825	781.4738	$C_{42}H_{70}O_{13}$	3.9	ginsenoside Rg8 orginsenoside Rh15	B1	781.4734, 619.4333, 457.3591	F13
244	26.09	[M + HCOO]⁻	1033.5597	1003.5583	$C_{51}H_{86}O_{21}$	1.4	pseudoginsenoside Rc1	PPD	987.5576,945.5412, 783.4890, 765.4788, 621.4348, 523.3785, 459.3879	F9

245	26.15	$[M-H]^-$	937.4748	937.4797	$C_{48}H_{74}O_{18}$	-5.2	OA-GlcA-Glc-Rha	OA	937.4748, 793.4334, 631.3696, 613.3835, 569.3837, 455.3534	F5
246	26.32	$[M-H]^-$	953.4803	953.4746	$C_{48}H_{74}O_{19}$	6	OA-Rha-Glc-GlcA	OA	777.4379, 597.3746, 537.3571.455.3534	F7
247	26.32	$[M+HCOO]^-$	985.5000	939.4953	$C_{48}H_{76}O_{18}$	-1.4	pseudoginsenoside Rt1 methyl ester	OA	777.4379, 597.3746, 537.3571, 455.3534	F7
248	26.34	$[M+HCOO]^-$	823.4470	777.4425	$C_{42}H_{66}O_{13}$	-6	OA-GlcA-Rha	OA	777.4014, 631.3806, 569.3837, 455.3534	F6
249	26.37	$[M+HCOO]^-$	823.4468	777.4425	$C_{42}H_{66}O_{13}$	-2.2	28-desglucosylchikusetsusaponin Ⅳ methyl ester or isomer	OA	777.4405,739.4195,455.3510	F17
250	26.43	$[M+HCOO]^-$	675.3762	631.3846	$C_{36}H_{56}O_{9}$	2.7	OA-GlcA	OA	631.3727,613.3727, 455.3534	F9
251	26.44	$[M+HCOO]^-$	999.5194	953.511	$C_{49}H_{78}O_{18}$	2.4	pseudoginsenoside Rt1 ethyl ester or isomer	OA	794.4547, 641.4053, 497.3655, 455.3628	F1
252	26.45	$[M+HCOO]^-$	823.4425	777.4425	$C_{42}H_{66}O_{13}$	-6.7	bipinoside A	OA	777.4347,731.4647,609.3859,537.3651,455.3422	F11
253	26.56	$[M+HCOO]^-$	829.4936	829.4949	$C_{43}H_{74}O_{15}$	-1.6	ginsenoside Rg3	PPD	783.4890, 621.4348, 459.3786	F14
254	26.57	$[M+HCOO]^-$	867.4760	821.4687	$C_{44}H_{70}O_{14}$	1.4	OA-GlcA-Glc-ethyl ester	OA	821.4638,659.4132,537.3571,455.3534	F1
255	26.63	$[M-H]^-$	835.4532	835.448	$C_{44}H_{68}O_{15}$	6.2	OA-GlcA-methy-Rha-Ac	OA	835.4532, 793.4457,645.4040,631.3915, 569.3837, 455.3441	F5
256	26.65	$[M+HCOO]^-$	867.4737	821.4687	$C_{44}H_{70}O_{14}$	-1.2	chikusetsusaponin Ⅳ a ethyl ester or isomer	OA	821.4638, 793.4334, 613.3835,455.3534	F8
257	26.65	$[M-H]^-$	645.4004	645.4003	$C_{37}H_{58}O_{9}$	-0.7	28-desglucosylchikusetsusaponin Ⅳa methyl ester	OA	645.3995,613.3746,455.3521	F7
258	26.72	$[M+HCOO]^-$	853.4568	807.4531	$C_{43}H_{68}O_{14}$	-2.7	spinasaponinA methyl ester or isomer	OA	645.4040, 455.3441	F5
259	26.73	$[M+HCOO]^-$	811.4815	765.4789	$C_{42}H_{70}O_{12}$	-3.6	ginsenoside F4 or isomer	C2	765.4788, 619.4225, 601.4159, 457.3685	F10
260	26.85	$[M+HCOO]^-$	867.4760	821.4687	$C_{44}H_{70}O_{14}$	1.4	chikusetsusaponin Ⅳ a ethyl ester or isomer	OA	821.4638,793.4379,613.3958,455.3534	F12
261	26.88	$[M-H]^-$	645.3993	645.4003	$C_{37}H_{58}O_{9}$	-2.3	28-desglucosylchikusetsusaponin Ⅳa methyl ester or isomer	OA	645.4040, 455.3534	F13
262	27.03	$[M-H]^-$	763.4161	762.4190	$C_{41}H_{64}O_{13}$	-1	28-desglucosylchikusetsusaponin Ⅳ	OA	763.4261,613.3727,569.3942,455.3441	F14

263	27.04	$[M + HCOO]^-$	811.4815	765.4789	$C_{42}H_{70}O_{12}$	-3.6	ginsenoside F4 or C2-Glc-Rha	C2	765.4788, 619.4225, 601.4159, 457.3685	F10
264	27.11	$[M + HCOO]^-$	837.4644	791.4582	$C_{43}H_{68}O_{13}$	0.3	OA-GlcA-Rha-methyl ester	OA	791.4567,777.4073,631.3806,613.3727,569 .3837,455.3518	F9
265	27.17	$[M + HCOO]^-$	837.4613	791.4582	$C_{43}H_{68}O_{13}$	-6	OA-GlcA-Rha-methyl ester	OA	791.4542, 777.4014, 631.3696, 537.3571, 455.3534	F16
266	27.19	$[M - H]^-$	777.4014	777.4061	$C_{41}H_{62}O_{14}$	-6	OA-GlcA-Xyl	OA	777.4014, 645.3596, 537.3571, 455.3534	F5
267	27.20	$[M + HCOO]^-$	679.4047	633.4003	$C_{36}H_{58}O_9$	-2.4	maslinic acid 28-O-β-d-glucopyranoside	A1	633.6429,471.3516,455.3628,411.3244	F3
268	27.23	$[M + HCOO]^-$	811.4856	765.4789	$C_{42}H_{70}O_{12}$	0.8	B3-Glc-Rha	B3	765.4788, 619.4225,457.3685	F14
269	27.31	$[M - H]^-$	1215.6667	1215.6679	$C_{65}H_{100}O_{21}$	-1.5	polyacetyleneginsenoside Ro	OA	1107.5760, 925.4799, 763.4382, 613.3619, 455.3534	F12
270	27.36	$[M + HCOO]^-$	837.5613	791.4582	$C_{43}H_{68}O_{13}$	-2.7	28-desglucosylchikusetsusaponin Ⅳ ethyl ester	OA	791.4542,763.4241,731.4323,631.3696,613 .3727,569.3837,537.3672,455.3534	F12
271	27.38	$[M - H]^-$	793.4360	793.4374	$C_{42}H_{66}O_{14}$	-2.5	OA-3-GlcA-Glc	OA	793.4334, 701.4296, 613.3727, 569.3837, 455.3534	F9
272	27.41	$[M + HCOO]^-$	825.4612	779.4582	$C_{42}H_{68}O_{13}$	-2.9	(OA+O)-GlcA-Rha	OA+O	779.4117,631.3915,471.3422, 455.3534	F12
273	27.45	$[M - H]^-$	793.4357	793.4374	$C_{42}H_{66}O_{14}$	-2.9	chikusetsusaponin II or OA-3-GlcA-Glc	OA	793.4334, 613.3727, 569.3837, 455.3534	F16
274	27.52	$[M - H]^-$	895.4645	895.4691	$C_{46}H_{72}O_{17}$	-5.1	OA-Xyl-Xyl-GlcA	OA	895.4645, 701.4296, 569.3837, 523.3785, 455.3534	F9
275*	27.53	$[M - H]^-$	793.4359	793.4374	$C_{42}H_{66}O_{14}$	-2.6	zingibroside R1	OA	793.4334, 613.3727, 569.3837, 455.3534	F11
276	27.54	$[M + HCOO]^-$	991.5472	945.5423	$C_{48}H_{82}O_{18}$	-1.2	gypenoside XVII or isomer	PPD	945.5412, 783.4890, 621.4456, 459.3879	F14
277	27.57	$[M - H]^-$	659.4146	659.4159	$C_{38}H_{60}O_9$	-2.8	28-desglucosylchikusetsusaponin Ⅳa ethyl ester	OA	659.4244, 631.4025,613.3727,569.3942, 455.3534	F16
278	27.63	$[M - H]^-$	793.4359	793.4374	$C_{42}H_{66}O_{14}$	-2.6	chikusetsusaponin II or OA-3-GlcA-Glc	OA	793.4334, 613.3727, 569.3837, 455.3534	F12
279	27.73	$[M - H]^-$	961.5353	961.5372	$C_{48}H_{82}O_{19}$	-2.3	chikusetsusaponin LM4	PPD	783.4871, 621.4345, 459.3827	F3
280	27.74	$[M + HCOO]^-$	961.5339	915.5317	$C_{47}H_{80}O_{17}$	-3.4	PPD-Glc-Glc-Xyl	PPD	915.5298, 783.4890, 621.4348, 459.3879	F14
281	27.87	$[M + HCOO]^-$	961.5339	915.5317	$C_{47}H_{80}O_{17}$	-3.4	PPD-Glc-Glc-Xyl	PPD	915.5298, 783.4890, 621.4348, 459.3879	F14
282	27.88	$[M - H]^-$	763.4248	763.4269	$C_{41}H_{64}O_{13}$	-3.5	OA-GlcA-Xyl	OA	763.4261, 613.3727, 569.3837, 455.3534	F8
283	27.93	$[M - H]^-$	777.4136	777.4061	$C_{41}H_{62}O_{14}$	9.6	28-desglucosylchikusetsusa	OA	777.4014, 763.4261,613.3727, 569.3837, 455.3534	F5

							ponin Ⅳ methyl ester or isomer			
284	28.04	$[M + HCOO]^-$	827.4825	781.4738	$C_{42}H_{70}O_{13}$	3.9	B3-GlcGlc	B3	781.4734, 619.4333, 457.3685,373.2753	F14
285	28.05	$[M - H]^-$	777.4014	777.4061	$C_{41}H_{62}O_{14}$	-6	28-desglucosylchikusetsusaponin Ⅳ methyl ester or isomer	OA	777.4014,763.4261,631.3806,613.3835,569.3837, 455.3534	F4
286	28.08	$[M - H]^-$	763.4267	763.4269	$C_{41}H_{64}O_{13}$	-1	OA-GlcA-Xyl	OA	763.4261, 613.3727, 569.3837, 455.3534	F6
287	28.16	$[M + HCOO]^-$	829.4948	783.4895	$C_{42}H_{72}O_{13}$	-0.8	ginsenoside F2	PPD	783.4877, 621.4361, 459.3828,375.2901	F15
288	28.27	$[M - H]^-$	763.4262	763.4269	$C_{41}H_{64}O_{13}$	-1.6	OA-GlcA-Xyl	OA	763.4261, 613.3727, 569.3837, 455.3534	F12
289	28.30	$[M + HCOO]^-$	829.4953	783.4895	$C_{42}H_{72}O_{13}$	-0.3	PPD-Glc-Glc	PPD	783.4890, 621.4456, 459.3786	F11
290	28.38	$[M - H]^-$	779.4239	779.4218	$C_{41}H_{64}O_{14}$	2.7	OA-GlcA-Xyl	OA	779.4239, 647.3802, 629.3741, 585.3748, 523.3785,455.3628	F5
291	28.39	$[M + HCOO]^-$	829.4945	783.4895	$C_{42}H_{72}O_{13}$	-1.2	ginsenoside F2 or isomer	PPD	783.4877, 621.4361, 459.3828,375.2901	F15
292	28.43	$[M - H]^-$	763.4263	763.4269	$C_{41}H_{64}O_{13}$	-1.5	OA-GlcA-Xyl	OA	763.4261, 613.3727, 569.3837, 497.3655, 455.3534	F7
293	28.44	$[M + HCOO]^-$	829.4951	783.4895	$C_{42}H_{72}O_{13}$	-0.5	ginsenoside Rg3 or isomer	PPD	783.4878, 621.4361, 459.3828,375.2902	F14
294	28.62	$[M + HCOO]^-$	853.4962	807.4895	$C_{44}H_{72}O_{13}$	0.9	acetyl-ginsenoside F4	C2	807.4933,765.4908, 619.4116, 457.3778	F9
295	28.67	$[M + HCOO]^-$	829.4951	783.4895	$C_{42}H_{72}O_{13}$	-0.5	ginsenoside Rg3 or isomer	PPD	783.4878, 621.4361, 459.3828,375.2902	F14
296	28.67	$[M + HCOO]^-$	693.3915	647.3795	$C_{36}H_{56}O_{10}$	1.1	(OA+O)-GlcA	OA+O	647.3802,471.3611,455.3441	F8
297	28.68	$[M - H]^-$	749.4457	749.4476	$C_{41}H_{66}O_{12}$	-3.3	pjs-4	OA	749.4462, 647.3802, 599.3936, 439.3568	F16
298	28.87	$[M - H]^-$	781.4734	781.4738	$C_{42}H_{70}O_{13}$	-0.5	OA-GlcA-Rha	OA	617.2941,569.3733,439.9428	F5
299	29.01	$[M - H]^-$	631.3848	631.3846	$C_{36}H_{56}O_{9}$	-0.6	OA-GlcA	OA	631.3806, 555.3622, 455.3534	F4
300*	29.09	$[M - H]^-$	631.3841	631.3846	$C_{36}H_{56}O_{9}$	-6.3	calenduloside E	OA	631.3806,455.3534	F16
301	29.57	$[M + HCOO]^-$	663.409	617.4053	$C_{36}H_{58}O_{8}$	-3.6	oleanolic acid 28-O-β-D-glucopyranose	OA	617.4135,581.2194,455.3534,407.3310	F9
302	29.65	$[M - H]^-$	631.3806	617.4053	$C_{36}H_{58}O_{8}$	0.9	OA-GlcA	OA	631.3806,613.3619,455.3534	F5
303	29.65	$[M + HCOO]^-$	871.5041	825.5000	$C_{44}H_{74}O_{14}$	-1.3	acetyl-ginsenoside Rg3	PPD	825.4989,783.4890,621.4348,603.4310,459.3879	F14
304	30.16	$[M - H]^-$	823.4494	777.4425	$C_{42}H_{66}O_{13}$	1.1	28-desglucosylchikusetsusaponin Ⅳ methyl ester or isomer	OA	777.4501,763.4305,537.3607,455.3628	F6

305	30.96	[M + HCOO]⁻	811.4856	765.4789	$C_{42}H_{70}O_{12}$	-3.6	ginsenoside Rg5 or isomer	C2	765.4788, 603.4202,457.0043,376.9831	F14
306	31.21	[M + HCOO]⁻	811.4856	765.4789	$C_{42}H_{70}O_{12}$	-3.6	ginsenoside Rg5 or isomer	C2	765.4788, 603.4202,457.0043,376.9916	F14
307*	34.77	[M − H]⁻	455.3529	455.3525	$C_{30}H_{48}O_3$	-0.4	Oleanolic acid	OA	455.3534	F8

* Characterized by compared with reference compounds.

The structures of sapogenins in the table are as follows:

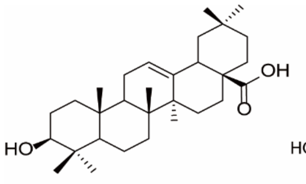

OA $C_{30}H_{48}O_3$ 456.3603

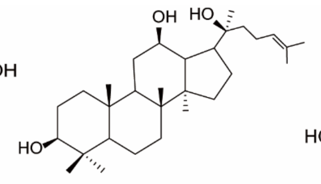

PPD $C_{30}H_{52}O_3$ 460.3916

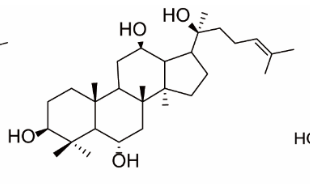

PPT $C_{30}H_{52}O_4$ 476.3866

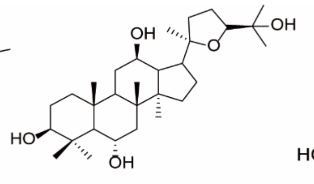

OT $C_{30}H_{52}O_5$ 492.3815

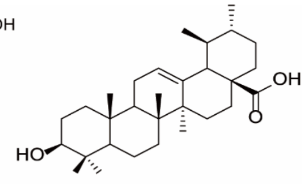

UA $C_{30}H_{48}O_3$ 456.3603

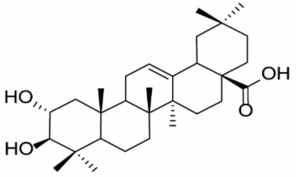

A1 $C_{30}H_{48}O_4$ 472.3553

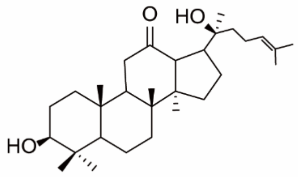

B1 $C_{30}H_{50}O_3$ 458.3760

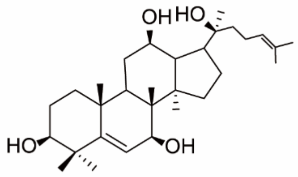

B2 $C_{30}H_{50}O_4$ 474.3709

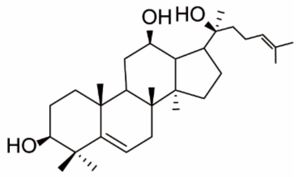

B3 $C_{30}H_{50}O_3$ 458.3760

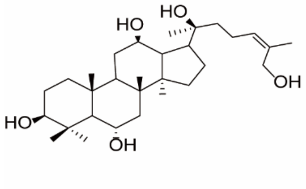

C1 $C_{30}H_{52}O_5$ 492.3815

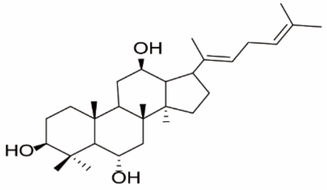

C2 $C_{30}H_{50}O_3$ 458.3760

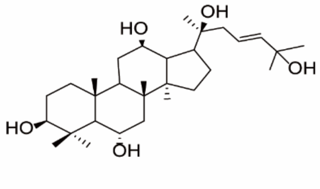

C3 $C_{30}H_{52}O_5$ 492.3815

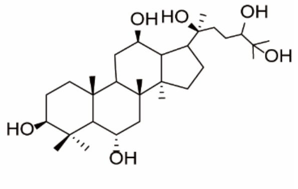

C4 $C_{30}H_{54}O_6$ 510.3920

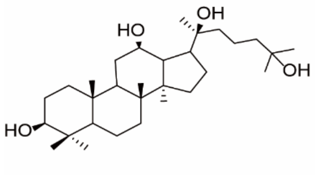

D1 $C_{30}H_{54}O_4$ 478.4022

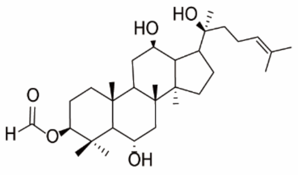

D2 $C_{31}H_{52}O_5$ 504.3815

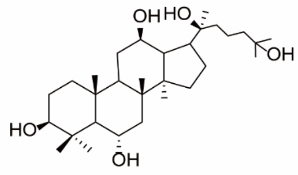

D3 $C_{30}H_{54}O_5$ 494.3971

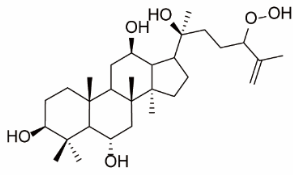

D4 $C_{30}H_{52}O_6$ 508.3764
